# Divergent ynamide reactivity in the presence of azides – an experimental and computational study†
†Electronic supplementary information (ESI) available. See DOI: 10.1039/c6sc01945e


**DOI:** 10.1039/c6sc01945e

**Published:** 2016-06-10

**Authors:** Veronica Tona, Stefan A. Ruider, Martin Berger, Saad Shaaban, Mohan Padmanaban, Lan-Gui Xie, Leticia González, Nuno Maulide

**Affiliations:** a Institute of Organic ChemistryFaculty of Chemistry , University of Vienna , Währinger Straße 38 , 1090 Vienna , Austria . Email: nuno.maulide@univie.ac.at; b Institute of Theoretical Chemistry , Faculty of Chemistry , University of Vienna , Währinger Straße 17 , 1090 Vienna , Austria

## Abstract

An unusually divergent reactivity of ynamides in the presence of azides is reported. This new keteniminium-based methodology, which only requires triflic acid as promoter, facilitates access to β-enaminoamides and biologically important oxazolidine-2,4-diones in a highly selective, divergent manner that is fully controllable by the present azide. A mechanistic rationale for these divergent reaction pathways is delineated and supported by extensive density functional theory analyses, as well as selected mechanistic experiments.

## Abstract 2

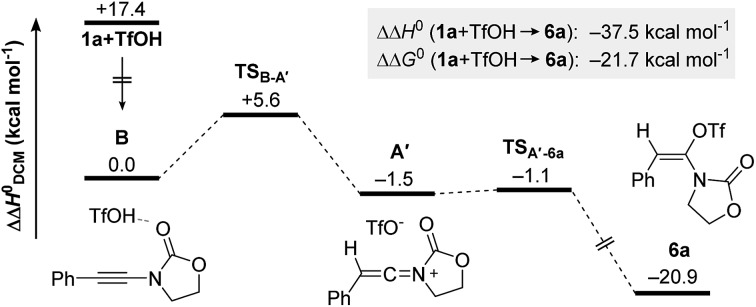
An unusually divergent reactivity of ynamides in the presence of azides is reported.

## Introduction

**Scheme 1:**
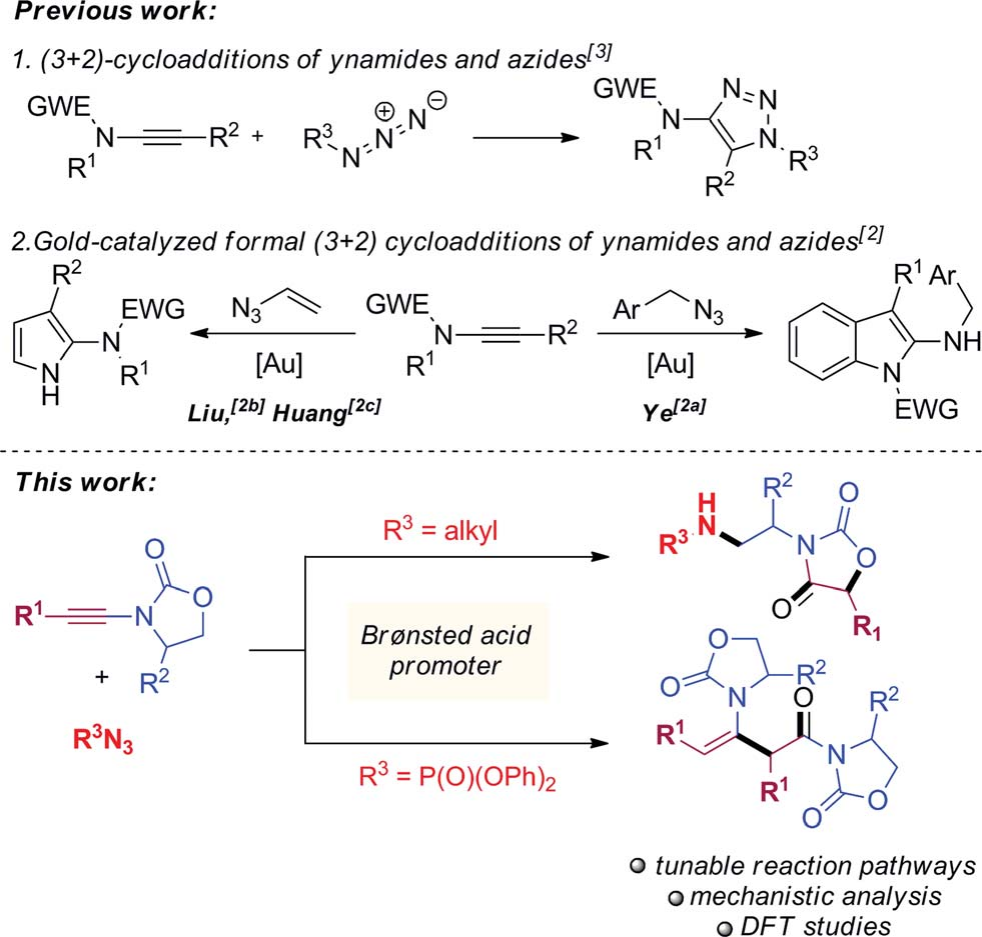
Prior work on the reactivity of ynamides and azides (top) and divergent reactivity under Brønsted acid catalysis (bottom).

Ynamides are well established as versatile reagents in organic synthesis, as the unique polarization of their triple bond continues to enable broad synthetic applications.^1^ In this context, ynamides thrive as building blocks for the synthesis of complex organic frameworks (which would be difficult to achieve otherwise) and biologically relevant heterocycles. Somewhat surprisingly, most of the contemporary efforts on ynamide chemistry appear to focus on their activation by carbophilic transition metals. Herein, we report the unusually divergent reactivity of ynamides in the presence of azides under promotion by a simple Brønsted acid (Scheme 1, bottom).

With the notable exceptions of Ye's, Liu's and Huang's recent elegant works on the gold-catalyzed syntheses of indole- and pyrrole-derivatives,^2^ the activation of ynamides with azides has hitherto been comprehensively utilized for the synthesis of triazoles through (3 + 2) cycloaddition (Scheme 1, top).^3^ Our work documents a novel reactivity pattern of azide/ynamide systems leading to the divergent and controlled formation of either oxazolidine-2,4-diones or β-enaminoamides (Scheme 1, bottom). In more detail, the first part of our work describes the formation of oxazolidine-2,4-diones *via* a complex azide-triggered rearrangement. These scaffolds have rich biological activity^4^ and can be employed as monomers for the synthesis of side-chain modified (SCM) polypeptides.^5^

In the second part, a change of azide partner strikingly promoted formation of radically different, quasi-dimerization products. The dimerization of alkynes in general is rare in the absence of metal promoters,^6^ and the only previously reported dimerization of ynamides, by Skrydstrup,^7^ involves the use of a gold catalyst. Mechanistic studies and extensive density functional theory (DFT) calculations shed light on the details of these unique examples of divergent reaction pathways.

## Results and discussion

**Scheme 2:**
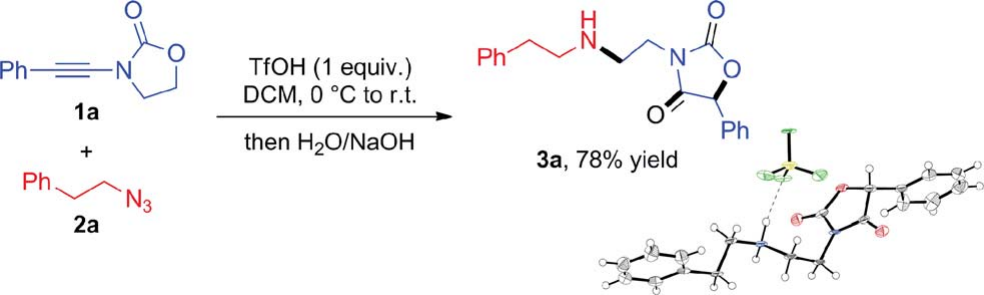
Unexpected coupling of **1a** with **2a** under acidic conditions to generate the oxazolidine-2,4-dione **3a** (X-ray structure shown; newly formed bonds highlighted in bold).

From the outset, attempts at combining phenyl-substituted ynamide **1a** and the azide **2a** under acidic conditions unexpectedly led to the rearranged product **3a**, the structure of which was elucidated by X-ray analysis (Scheme 2). Additional screening revealed dichloromethane (DCM) to be the best solvent and TfOH to be necessary to promote the reaction (details of optimization experiments are compiled in the ESI†). This enabled the preparation of **3a** in 78% isolated yield^8^ (X-ray structure in Scheme 2).

Given the unusual skeletal reorganization involved in the coupling of **1a** and **2a** into **3a** (newly formed bonds highlighted in bold typeset in Scheme 2), along with the known biological relevance of oxazolidine-2,4-diones,^9^ we were eager to probe the generality of this transformation and decided to investigate the use of diverse ynamide and azide partners.

**Scheme 3:**
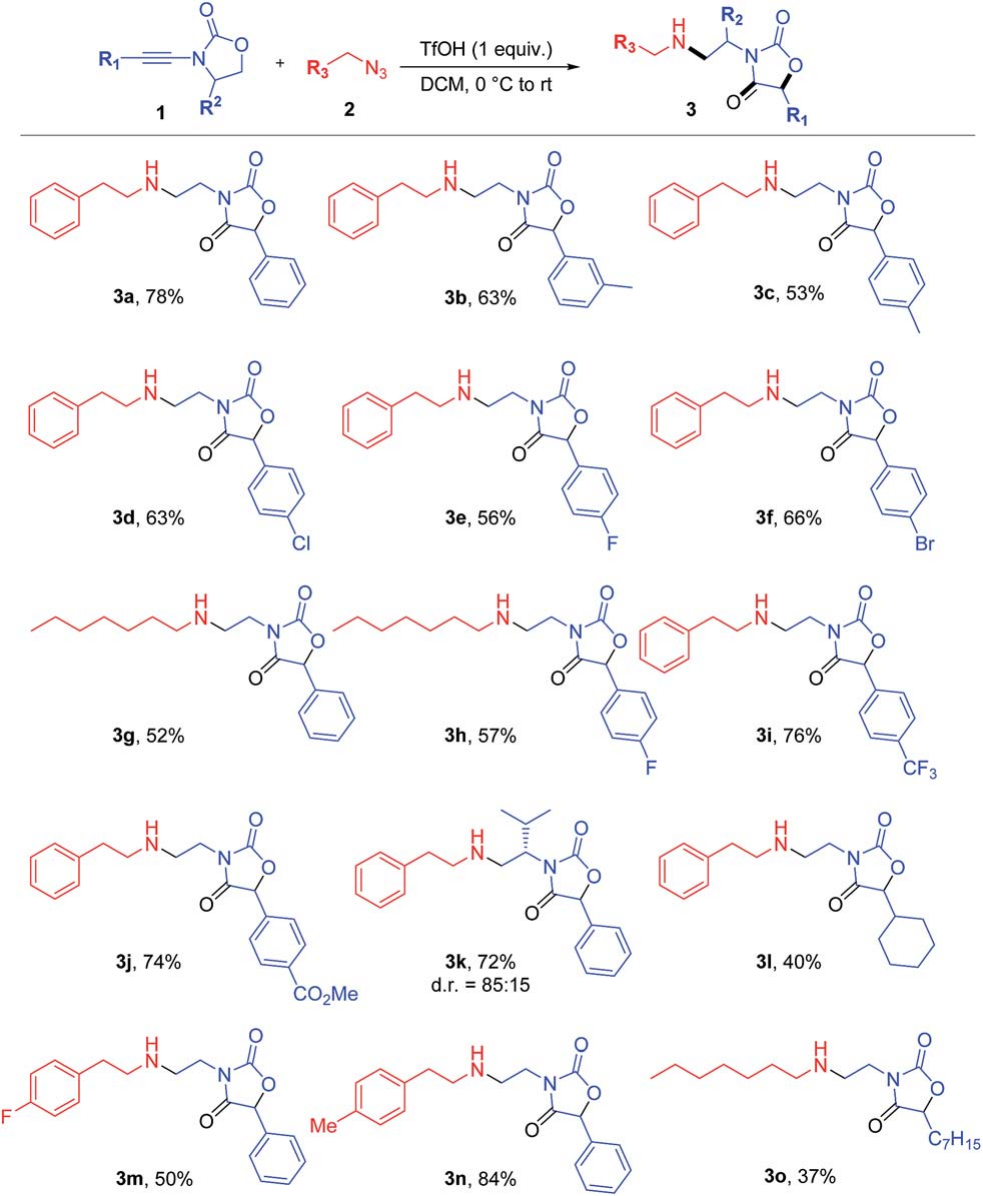
Scope of azides and ynamides in the coupling towards oxazolidine-2,4-diones. *Reagents and conditions*: **1** (0.6 mmol), **2** (0.3 mmol) and TfOH (0.3 mmol) in DCM (1.5 mL). Yields refer to isolated products.^12^ For details, see the ESI.†

The results are depicted in Scheme 3. Aryl ynamides containing halogen substituents or electron-donating/withdrawing moieties on the aromatic ring led to the skeletal rearrangement products in generally good yields. The reaction was also performed with ynamides carrying a non-aromatic substituent with moderate yields (*cf.***3l**, Scheme 3). A diastereoselective variant was then investigated and the use of (*S*)-4-isopropyl-2-oxazolidinone as a chiral auxiliary led to product **3k**, formed with a d.r. of 85 : 15. Azides bearing fluorine and methyl substitutions also delivered the expected products in good yield (**3m** and **3n**). The use of an aliphatic azide led to the rearranged product in lower yield (**3o**).

During these investigations, we decided to look more in detail at the fate of the azide reaction partner. Considering that the efficiency of the reactions in Scheme 3 may be compromised by degradation of the azide under acidic conditions,^10^ we turned to the use of diphenylphosphoryl azide (dppa) (**4**) as a potentially more stable species.^11^

**Scheme 4:**
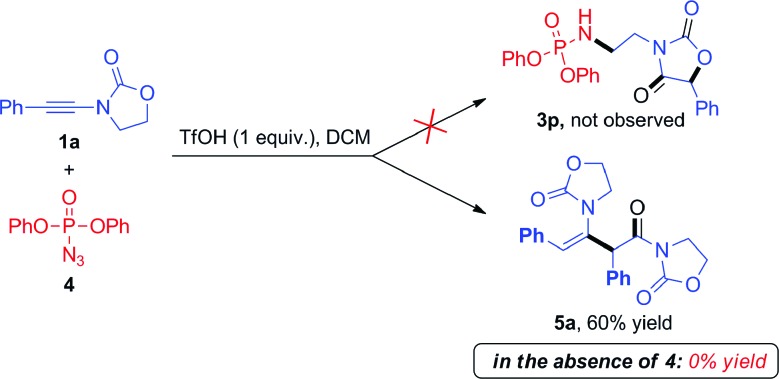
Unexpected dimerization of ynamide **1a** in the presence of dppa (**4**).

To our surprise, the use of dppa (**4**) under essentially the same conditions as those portrayed in Scheme 3 resulted in the exclusive formation of a new product, which was not the expected **3p**. Structural elucidation revealed this to be a dimer **5a** resulting from the union of two ynamide reactant molecules (Scheme 4). As mentioned previously, the direct dimerization of alkynes is almost exclusively the domain of transition-metal chemistry and the formation of a dimerization product under simple acidic conditions piqued our interest. Strikingly, the presence of dppa (**4**) was absolutely crucial for the formation of **5a**: in its absence, only the simple hydrolysis product of ynamide **1a** was detected, with no traces of any dimer.^13^

**Scheme 5:**
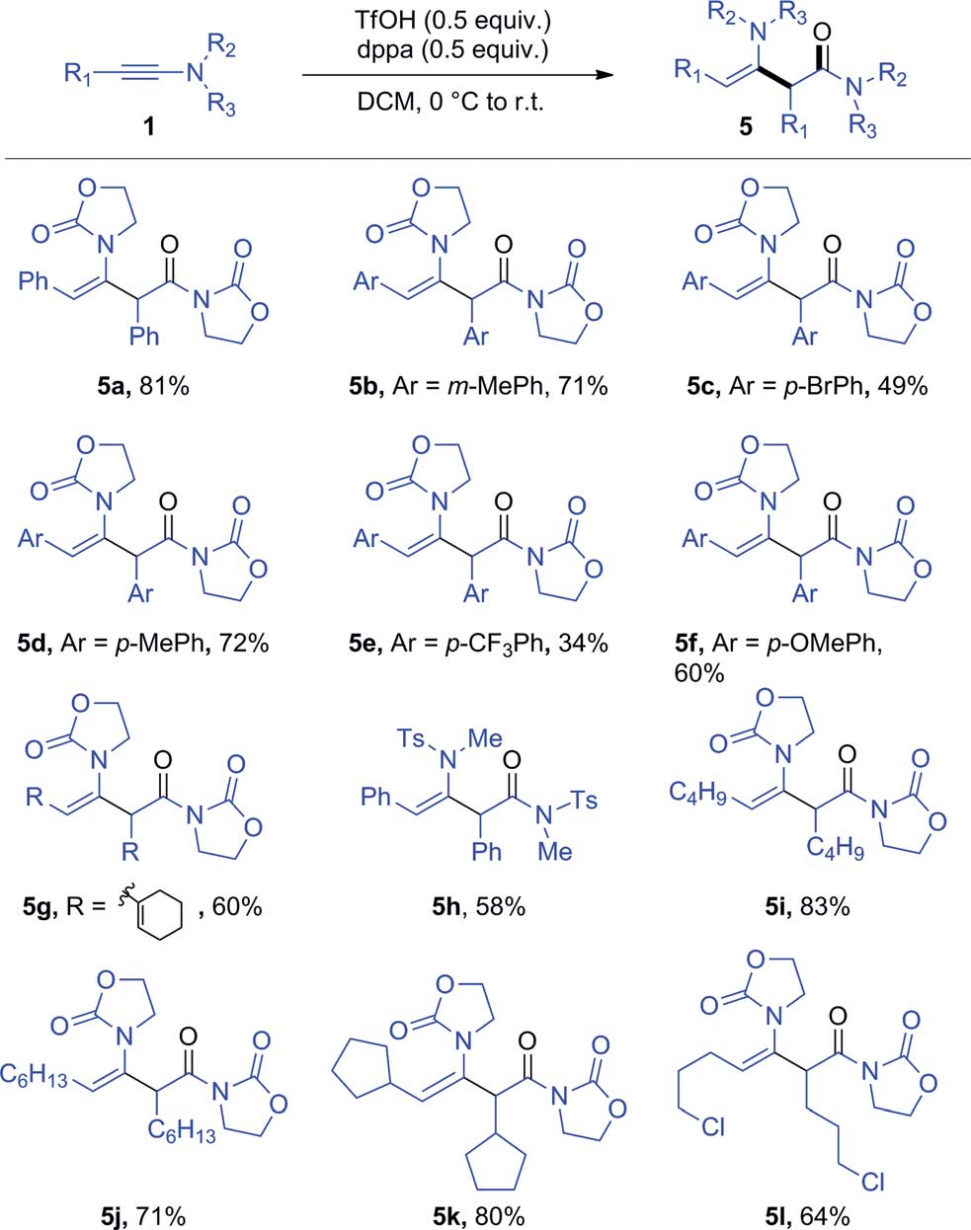
Scope of the dppa-promoted ynamide dimerization. *Reagents and conditions*: **1** (0.6 mmol), **4** (0.3 mmol) and TfOH (0.3 mmol) in DCM (1.5 mL). Yields refer to isolated products.

Further optimization enables the generation of this ynamide dimer in 81% yield in the presence of only substoichiometric amounts of both TfOH and dppa (**4**) (Scheme 5).^14^ With these new conditions in hand, we have been able to obtain the products of hydrative dimerization of differently substituted aryl ynamides. As shown in Scheme 5, several ynamides carrying either an aryl or alkyl side chain afforded good to excellent yields of the dimerization product. The use of an aryl-ynamide carrying a strongly electron-withdrawing CF_3_ moiety led, however, to a lower yield (*cf.***5e**). Importantly, sulfonylynamides also enable dimerization to take place (*cf.***5h**).

## Mechanistic studies

In order to understand the factors determining the different product outcomes observed experimentally and to gain insight into the mechanisms and energetics of these two divergent pathways, we turned to DFT calculations. Oxazolidinone **1a** served as the model substrate for all investigated processes. (2-Azidoethyl)benzene (**2a**) or dppa (**4**) were taken as the respective reaction partners.

### Computational methods

All DFT calculations were performed using the B3LYP hybrid functional^15^ with the D3 dispersion correction of Grimme^16^ and the polarized double-ζ basis set 6-31G*,^17^ as implemented in the Gaussian09 program package.^18^ The effect of solvation was taken into account implicitly by adding a polarizable continuum model, with dichloromethane as solvent (dielectric constant 8.93).^19^ Geometry optimizations were carried out without any constraints. Ground state minima and transition states were confirmed by frequency calculations, yielding none and one imaginary frequency, respectively. The connectivity of the transition state structures was verified by intrinsic reaction coordinate calculations.^20^ Electronic energies obtained were converted to relative enthalpies Δ*H*^0^ and free energies Δ*G*^0^ at 298.15 K and 1 atm by using zero-point energy and thermal energy unscaled corrections obtained in the frequency calculation. All energies are reported in kcal mol^–1^ and all distances are reported in Ångstrom (Å).

### Preliminary DFT studies

**Scheme 6:**
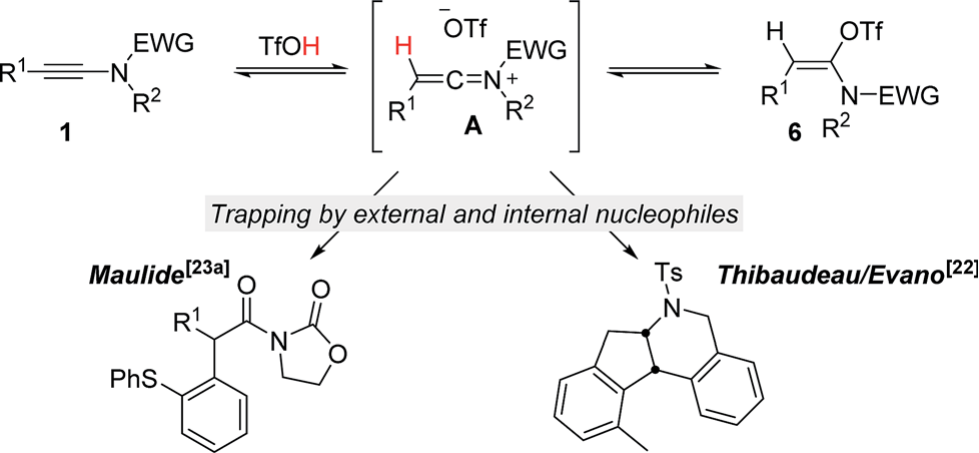
Established characteristics on the reaction of ynamides with TfOH.

It is well recognized that the treatment of ynamides with TfOH leads to the formation of transient keteniminium triflates **A** (Scheme 6).^21^ These reactive species rapidly collapse – in the absence of any nucleophilic reaction partner – to the energetically more stable *N*,*O*-ketene aminals **6**.^22^ Although any attempt to characterize these reactive intermediates using spectroscopic methods was met with failure,^22^,^23^ we and others have disclosed chemical transformations involving the discrete intermediacy of keteniminium triflates.^22^,23a,^24^ This, however, raises the question as to the true nature of the stability and reactivity of these transient keteniminium species **A** – in particular in the presence of additional reagents.

**Fig. 1:**
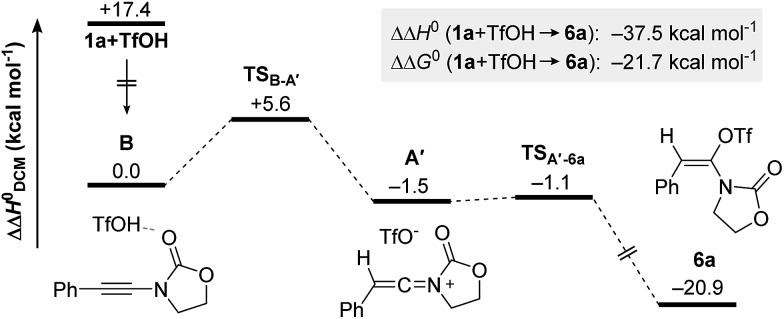
Computed reaction profile for the formation of *N*,*O*-ketene acetal **6a** from ynamide **1a** and TfOH. Relative enthalpy values ΔΔ*H*^0^ are given in relation to association complex **B**.

Intrigued by the fundamental question on the stability of keteniminiums **A**, and in order to better understand the exact nature of the interaction between reagents/additives and that species, we decided to begin our computational studies by simply modelling the generation of aminal **6a** (R^1^ = Ph, N(R^2^)EWG = oxazolidinone) from ynamide **1a** in the absence of any additional reaction partners, such as (2-azidoethyl)benzene (**2a**) or dppa (**4**). As shown in Fig. 1, after initial association of TfOH and ynamide **1a**, which is highly exothermic in energy (ΔΔ*H*^0^ = –17.4 kcal mol^–1^), protonation of the ynamide in complex **B** occurs at the β-carbon providing keteniminium triflate **A′** (**TS_B–A′_**). Iminium **A′** was found to collapse without any significant activation barrier (ΔΔ*H*^0^ ≪ 1.0 kcal mol^–1^) to *N*,*O*-ketene aminal **6a** (**TS_A′–6a_**). This result is in accordance with the spectroscopic studies carried out by Thibaudeau and Evano.^22^ Moreover, the low barrier of activation of the triflate-addition to keteniminium **A′** explains why the experimental characterization of any intermediate keteniminium is highly challenging. Overall, the conversion of ynamide **1a** to aminal **6a** is both highly exothermic (ΔΔ*H*^0^ = –21.7 kcal mol^–1^) and exergonic (ΔΔ*G*^0^ = –37.5 kcal mol^–1^) in energy.

### DFT results for oxazolidine-2,4-dione formation and experimental support

**Scheme 7:**
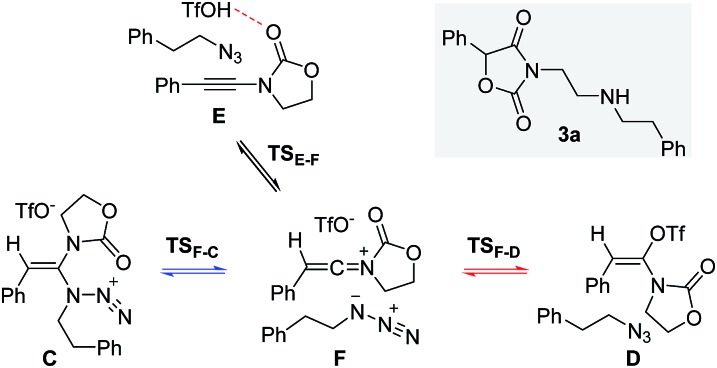
Proposed initial steps in the formation of oxazolidine-2,4-dione **3a**.

Next, we embarked on DFT calculations for the reaction of ynamide **1a**, TfOH and (2-azidoethyl)benzene (**2a**). At the outset of the mechanistic theoretical investigations on the formation of oxazolidine-2,4-dione **3a**, we evaluated the competitive formation of *N*,*N*-ketene acetal **C***vs. N*,*O*-ketene acetal **D** (Scheme 7).

**Fig. 2:**
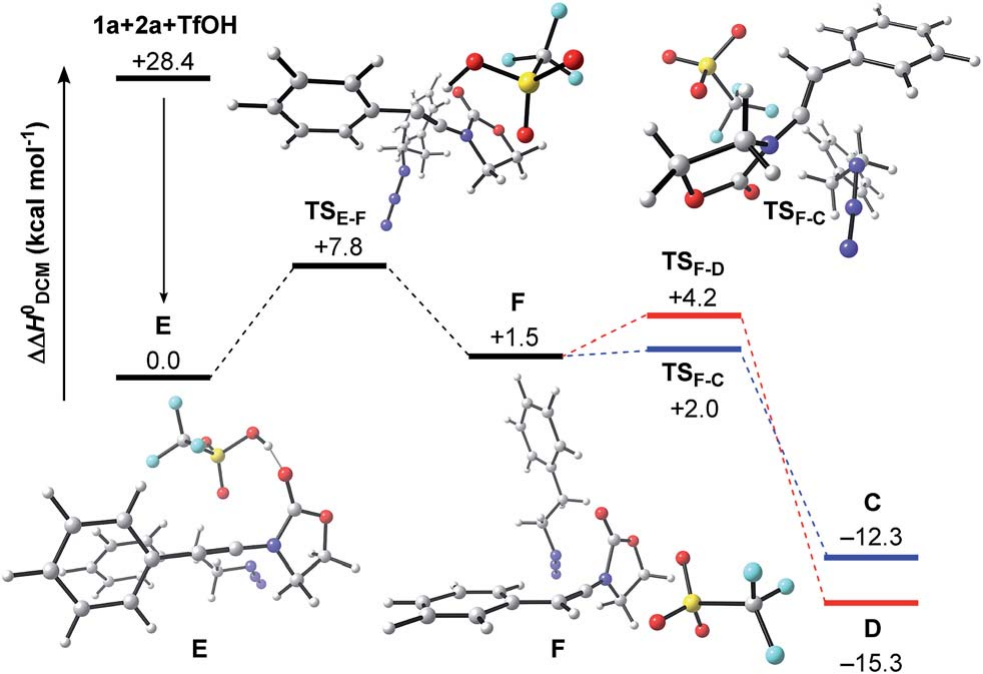
Computed reaction profile for the transformation of ynamide **1a** and azide **2a** into ketene acetals **C** or **D**. Relative enthalpy values ΔΔ*H*^0^ are given in relation to association complex **E**.

The related enthalpy profile for the proposed underlying mechanism is presented in Fig. 2. In accordance with the preliminary studies, pre-association of all three reagents involved (ynamide **1a** + TfOH + azide **2a** = complex **E**) is accompanied by a large gain in energy (ΔΔ*H*^0^ = –28.4 kcal mol^–1^). Following migration of TfOH to the electron-rich alkynyl moiety, protonation at the β-carbon ensues, leading to keteniminium triflate **F** (**TS_E–F_**) in a slightly endothermic process (ΔΔ*H*^0^ = +1.5 kcal mol^–1^). The obtained iminium **F** can then follow two competitive pathways: (1) triflate addition generating the thermodynamically more stable *N*,*O*-ketene acetal **D** (**TS_F–D_**) or (2) azide addition forming *N*,*N*-ketene acetal **C** (**TS_F–C_**), the kinetic product. Three points are worth mentioning: first, in light of the low barriers of activation for both processes (ΔΔ*H*^0^ ≤ 2.7 kcal mol^–1^), keteniminium **F** can be considered to be a rather short-lived intermediate. Second, in comparison with the isolated system (Scheme 6, Fig. 1), the activation barrier for triflate addition is energetically more demanding, albeit the difference is small ΔΔ*H*^0^ = +2.3 kcal mol^–1^.^25^ Third, the low difference in activation energies (**TS_F–D_***vs.***TS_F–C_**) would suggest that both ketene acetals **C** and **D** are present as discrete intermediates towards the generation of oxazolidine-2,4-dione **3a**.

**Fig. 3:**
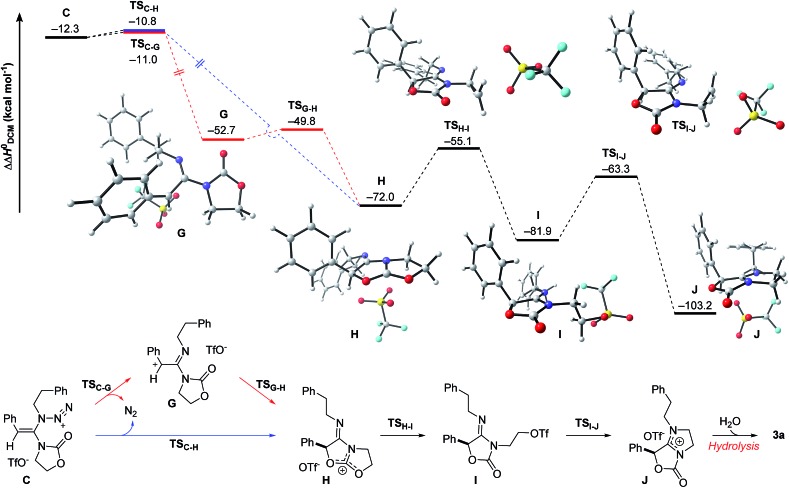
Computed reaction profile for the conversion of *N*,*N*-ketene acetal **C** into amidinium triflate **J**. Relative enthalpy values ΔΔ*H*^0^ are given in relation to association complex **E**.

**Fig. 4:**
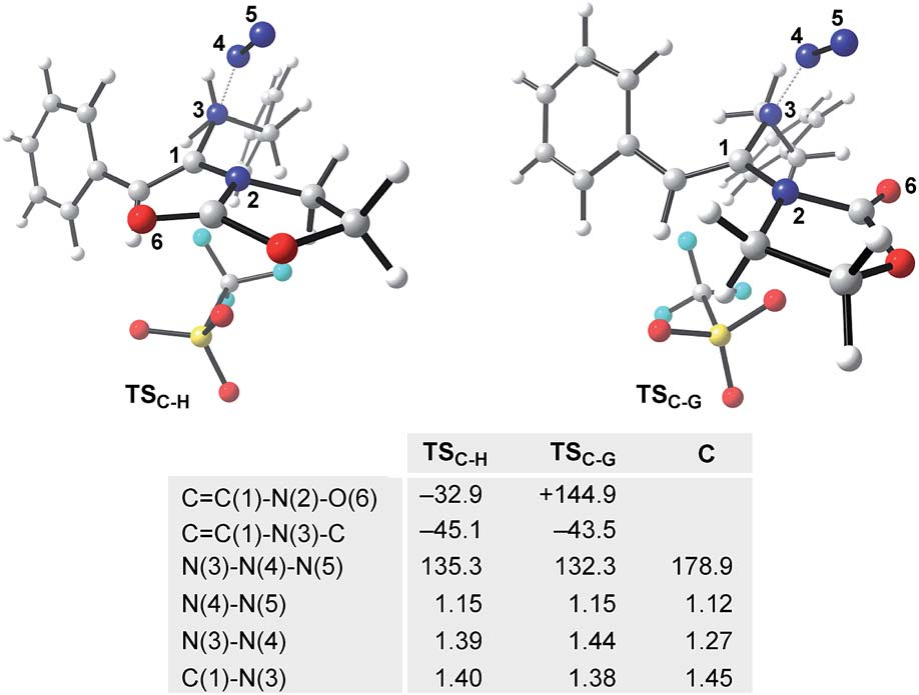
DFT-optimized transition states **TS_C–H_** and **TS_C–G_** for the proposed nitrogen extrusion step. Selected dihedral angles and angles are given in °; key bond distances are stated in Å and set in comparison to the optimized geometry of azide **C**.

The final steps *en route* to **3a** are depicted in Fig. 3. Although calculations have indicated that *N*,*O*-ketene acetal **D** is favored over *N*,*N*-ketene acetal **C** by ΔΔ*G*^0^ = 6.8 kcal mol^–1^, DFT-studies have attributed further viable reaction pathways only to acetal product **C**.^26^ In light of the energetically accessible transition state **TS_F–D_** in the reverse direction (**D** → **F**), it appears reasonable to assume that upon consumption of available **C**, successive displacement of the equilibrium **D** ⇌ **F** ⇌ **C** would ensure full conversion. As shown in Fig. 3, once *N*,*N*-ketene acetal **C** is formed, it can undergo spontaneous loss of dinitrogen. Interestingly, computations have revealed that the initial orientation of the oxazolidinone moiety concurrent to the dinitrogen expulsion dictates the presence (**TS_C–G_**) or the absence (**TS_C–H_**) of a stable cationic intermediate **G***en route* to oxazolium triflate **H** (Fig. 4). Thus, if the carbonyl oxygen, O(6), points towards the emerging benzylic cationic system (**TS_C–H_**) the resulting transient cation **G′** (not shown) collapses in a second, yet barrierless step into bicycle **H**.^27^ Conversely, if the carbonyl oxygen and the benzylic cation adopt a quasi-*anti*-periplanar conformation (*Φ*(C=C(1)–N(2)–O(6)) = +145, Fig. 4) during the nitrogen-extrusion, imine **G** is formed as a stable intermediate. The identified torsional transition state **TS_G–H_** connecting imine **G** and oxazolium **H** is characterized by the counter-clockwise rotation of the oxazolidinone moiety with respect to the axis as defined by the N(2)–C(1) bond.^28^ Notably, since the energetics of these two described nitrogen-extruding transition states are very close, we anticipate that both pathways are operative for aryl-bearing ynamides, such as **1a**. Further characteristic features of these key transition states are (Fig. 4): (1) the rather long incipient N(4)–N(5) and C(1)–N(3) bond lengths, as well as the rather short incipient N(3)–N(4) bond length, indicative of early transition states. (2) The assistance of the double bond (π(C=C(1))) in the rupture of the N(3)–N(4) bond, as judged by both NBO and MO analyses. Key to this observation is the existence of a distinct orbital in the highest occupied molecular orbital (HOMO) of both transition states (**TS_C–H_** and **TS_C–G_**) connecting all three bonding atoms of the enamine-like system (C=C(1)–N(3)).^29^ Associated to this finding is (3) the stereodefined nitrogen extrusion, servicing the stereoselective formation of (*E*)-imines **G** and **G′**, a necessary prerequisite for the further pathway (*vide infra*). In accordance with the above statements made, systems hypothetically leading to (*Z*)-imines lack the crucial orbital overlap between π(C=C(1)) and σ*(N(3)–N(4)), thus rendering these pathways highly unlikely.^30^

As shown in Fig. 3, the proposed onward reaction of oxazolium triflate **H** to **3a** involves two successive S_N_2 reactions (**H** → **I** and **I** → **J**). The first of these involves nucleophilic opening of the oxazolium moiety (**TS_H–I_**) giving rise to primary triflate **I**. A structurally different bicycle is then reformed by intramolecular ring closure *via* substitution of the primary triflate by the amidine functionality in **I** (**TS_I–J_**) generating the final intermediate, amidinium triflate **J**. Both transition-state geometries (**TS_H–I_** and **TS_I–J_**) nicely reflect the second-order character of the nucleophilic substitutions typical for primary alkyl reaction centers.

The triflate-mediated rearrangement of oxazolium **H** into amidinium **J** represents a rather unusual, yet intriguing process. In light of the feasible (ΔΔ*G*^0^(**TS_H–I_**) = +17.6 kcal mol^–1^) and favorable (ΔΔ*G*^0^(**H** → **I**) = –10.5 kcal mol^–1^) computed energetics of the ring-opening reaction of oxazolium **H**, our proposed mechanism marks one of the few cases of nucleophilic assistance of triflates in chemical transformations. Straightforward aqueous basic hydrolysis of amidinium triflate **J** during work-up gives rise to oxazolidine-2,4-dione **3a**.

**Scheme 8:**
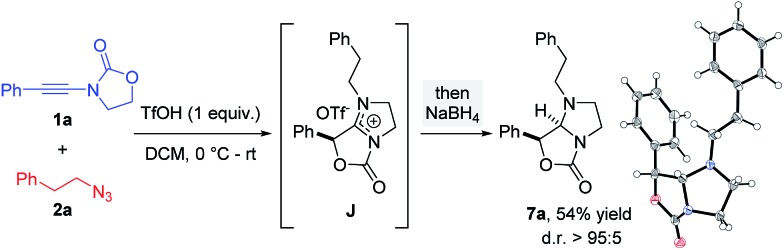
Interception of amidinium **J** by reduction (X-ray structure of **7a** depicted).^31^

The overall free energy of the conversion of association complex **E** (=**1a** + **2a** + TfOH) into imidazolium triflate **J**, at –109.9 kcal mol^–1^, is evidently high, with the loss of dinitrogen (**C** → **G**/**G′** → **H**: ΔΔ*G*^0^ = –75.6 kcal mol^–1^) representing the main driving force of the reaction. Since all of the individual barriers towards the formation of oxazolium **H** are fairly small, the reaction is believed to proceed smoothly up to this point; the subsequent rearrangement of the bicycle, however, contains rather high barriers of activation (ΔΔ*G*^0^ = +17.6 and +19.4 kcal mol^–1^, respectively). The triflate-mediated formation of amidinium **J** therefore represents the rate-limiting process. Since the amidinium hydrolysis only occurs upon addition of water, we anticipated that the final intermediate **J** could also serve as a valuable platform for other synthetic transformations. In the event (Scheme 8), treatment of the reaction mixture obtained by combining **1a** and **2a** in the conditions previously described after 24 hours with NaBH_4_, afforded a new compound. Pleasingly, X-ray crystallographic analysis showed this to be the expected *N*,*N*-acetal **7a**,^31^ formed as a single *trans*-diastereoisomer, thus lending strong support to the intermediacy of **J** prior to hydrolysis.

### DFT results for hydrative dimerization and experimental validation

**Fig. 5:**
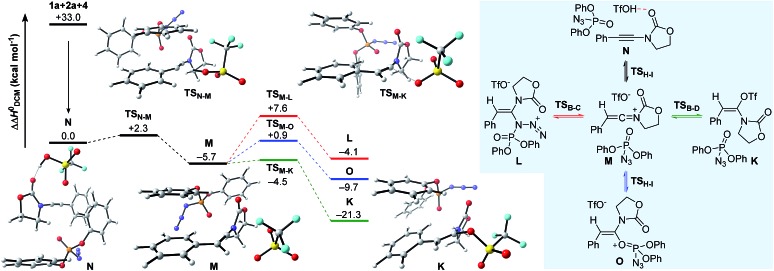
Computed reaction profile for the conversion of **1a** into *N*,*O*-ketene acetal **K** in the presence of dppa (**4**). Relative enthalpy values ΔΔ*H*^0^ are stated with respect to association complex **N**.

Having established plausible mechanisms for the conversion of ynamides **1** into *N*,*O*-ketene aminals **6**, as well as for the reaction of alkyl azides **2** with ynamides **1**, we next investigated the effect of dppa (**4**) in leading to the spontaneous dimerization of ynamides, such as **1a**. In alignment with the studies presented above, we initially considered the formation of *N*,*O*- and *N*,*N*-ketene acetals **K** and **L** expecting to gain valuable insights into the exact nature of the interaction between dppa (**4**) and the transient keteniminium triflate **M**. The computational results on the proposed mechanistic pathway, along with the schematic representation of the initial competitive pathways are delineated in Fig. 5. As shown before, pre-association of the reaction partners, ynamide **1a**, dppa (**4**) and TfOH, proceeds with a large gain in energy (ΔΔ*H*^0^ = –33.0 kcal mol^–1^) forming complex **N**. Subsequent protonation at the ynamide β-carbon (**TS_N–M_**) then affords keteniminium **M**. Interestingly, both the activation barrier for the protonation, as well as the energy of the transient keteniminium are markedly reduced compared to the isolated system (Fig. 1) and the reaction of ynamide **1a** with azide **2a** (Fig. 2). This can be ascribed to favorable π–π stacking interactions between the pendant phenolic arm of dppa (**4**) and the styrene-like system in the transition state geometry **TS_N–M_**, as well as the intermediate keteniminium **M**. The same holds true for the subsequent triflate addition step (**TS_M–K_**) leading to *N*,*O*-ketene acetal **K**. As a result, the calculated enthalpic profile for the conversion of keteniminium triflate **M** into dppa-associated acetal **K** closely resembles the energetic footprint of the isolated system. While this observation might appear – at first glance – inconsistent with the experimental observations, the exceptional ability of dppa (**4**) to interact with the styrene-motif in an energetically favorable mode, as observed in **TS_N–M_** → **K**, also proves to be vital for the occurring dimerization process in the case of aryl-bearing ynamides, such as **1a** (*vide infra*). It should be mentioned that we also investigated other processes including the addition of dppa (**4**) to the keteniminium species **M** in two ways: (1) azide-addition of dppa (**TS_M–L_**) affording oxazolidinone **L** and (2) addition of the phosphoryl oxygen in dppa (**4**) (**TS_M–O_**) leading to oxaphosphonium **O**. However, computations have indicated that both pathways are kinetically and thermodynamically disfavored with respect to the formation of *N*,*O*-ketene acetal **K**, and are therefore considered to be non-vital to the global picture.

**Fig. 6:**
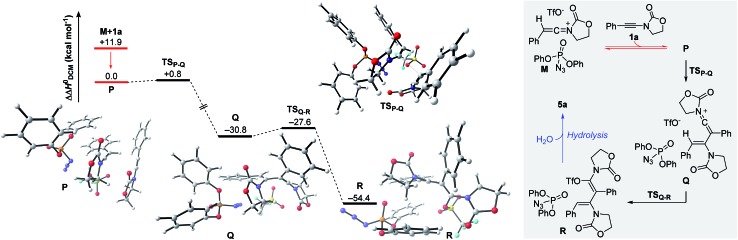
Computed reaction profile for the formation of *N*,*O*-ketene acetal **R**, hydrolysis-precursor to quasi-dimerization product **5a**. Relative enthalpy values ΔΔ*H*^0^ are stated with respect to association complex **P**.

The remaining steps in forming the dimer **5a** mandate the introduction of a second molecule of ynamide **1a**. As shown in Fig. 6, the combination of keteniminium **M** and an additional ynamide **1a** results in the exothermic formation (ΔΔ*H*^0^ = –11.9 kcal mol^–1^) of association complex **P**. Computations point out that the energetically most favorable spatial arrangement of all four components (dppa, keteniminium, triflate and ynamide) prefers the pseudo-substitution of the triflate counterion in **M** by ynamide **1a**. As a result, association complex **P** is decisively limited in its reactivity, as once an unreacted ynamide coordinates to a generated keteniminium species, any reaction pathway building up on the initial triflate-addition (as shown in Fig. 5) is shut down.

The generation of experimentally observed product **5a** hinges on the synthetic combination of an unreacted ynamide with a keteniminium species. Computations reveal that, in the presence of dppa (**4**), the barrier of activation for such a decisive transformative step (**TS_P–Q_**) is remarkably low (ΔΔ*H*^0^ = +0.8 kcal mol^–1^; ΔΔ*G*^0^ = +2.8 kcal mol^–1^). In detail, following migration and rotation of ynamide **1a** in complex **P** towards alignment of the β-carbon of **1a** and the positively charged keteniminium carbon (NBO charge = +0.52), a new C–C bond can arise forming keteniminium triflate **Q**. The addition **P** → **Q** is highly exothermic (ΔΔ*H*^0^ = –30.8 kcal mol^–1^) and exergonic (ΔΔ*G*^0^ = –26.9 kcal mol^–1^) in energy. As stated earlier, we consider the favorable π-π stacking interaction between the pendant phenol-arm of dppa (**4**) and the styrene-like motif in **TS_P–Q_** to be decisive for the occurrence of the hydrative dimerization of aryl-bearing ynamides, such as **1a**.^32^ This hypothesis is also corroborated by the experimental observation that both triphenylphosphate (P(O)(OPh)_3_) and ethoxyacetylene, both of which entail a Lewis-basic site as well as electron-rich π-systems, can also promote formation of the dimeric product **5a**.^16^

**Scheme 9:**
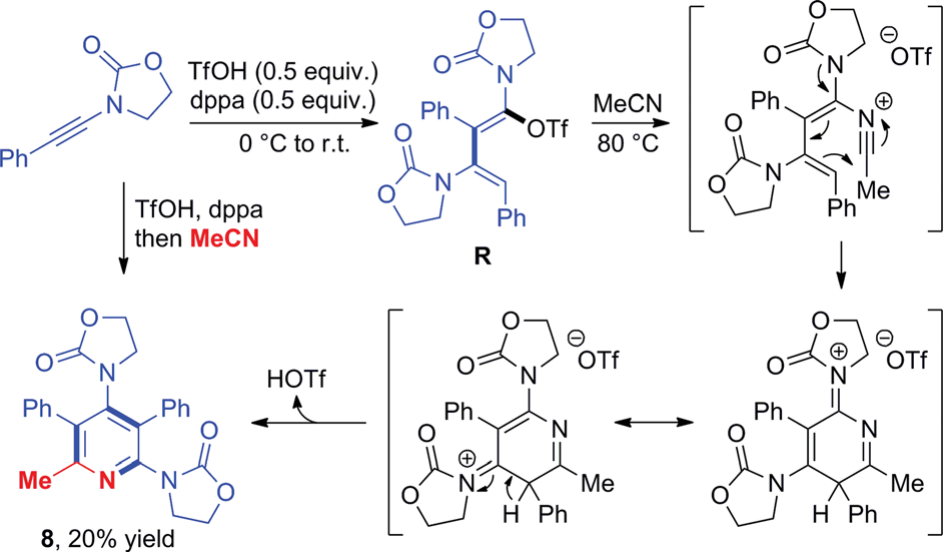
Formation of a pentasubstituted pyridine **8** by capture of intermediate **R** with acetonitrile and mechanistic proposal.

Triflate-addition (**TS_Q–R_**) generates the final intermediate, *N*,*O*-ketene acetal **R**. Once more, straightforward aqueous work-up furnishes the hydrative dimer **5a**. Importantly, we once more sought experimental evidence of the presence of **R** prior to hydrolysis. In the event, we found that adding acetonitrile to the reaction mixture of dimerization leads to the interesting formation of the congested, fully-substituted pyridine **8** in modest unoptimized yield (Scheme 9). This remarkable process likely involves capture of **R** by the nitrile nitrogen followed by cyclisation and aromatization, forming two new C–C bonds and one C–N bond overall.

In summary, the hydrative dimerization of ynamides is represented by the combination of the energy profiles in Fig. 5 and 6. The formation of *N*,*O*-ketene acetal **R** requires the overcoming of a maximum free energy barrier of only +4.2 kcal mol^–1^ (corresponding to the final transition state **TS_Q–R_**). Along with a free energy of reaction (**N** + **1a** → **R**) of –53.1 kcal mol^–1^ the process should occur rapidly under the reaction conditions employed.

**Scheme 10:**
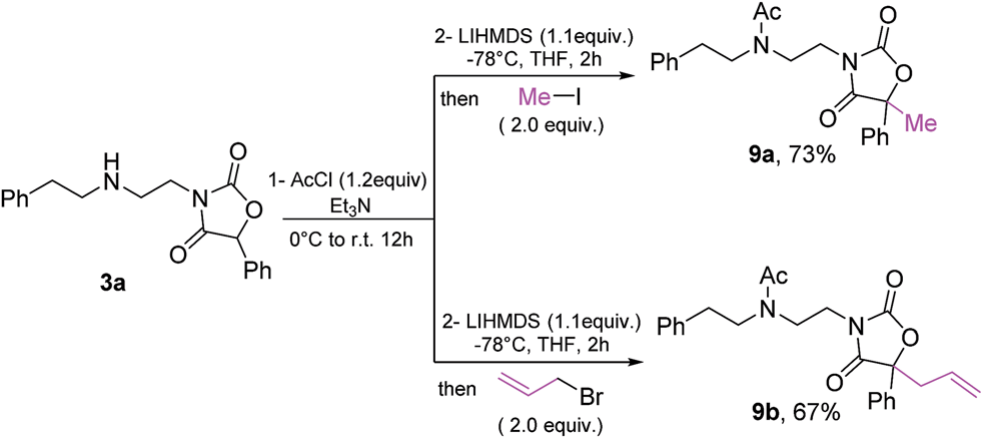
Derivatization of oxazolidine-dione **3a**.

Finally, the oxazolidine-dione products allow further manipulation, as exemplified by formation of fully substituted derivatives **9a**/**9b**^33^ (Scheme 10).

## Conclusions

In conclusion, we have documented an unusually divergent reactivity mode of ynamides in the presence of azides to provide either β-enaminoamides or biologically important oxazolidine-2,4-diones in a concise and highly selective manner. The formation of either product is fully controlled by the azide partner under the influence of a simple Brønsted acid promoter. The presented work therewith displays a rare example of a Brønsted acid-mediated, divergent reaction that leads to radically different products. Extensive DFT calculations combined with designed mechanistic experiments provided detailed insights into the underlying mechanistic pathways. The mechanistic investigations also highlighted the crucial factors key to each pathway and presented important information about the general stability and reactivity of transient keteniminium species in the presence or absence of additional reagents. Given the fundamental importance of ynamides in organic synthesis, our mechanistic insights may well evoke the development of novel, conceptually intriguing methods involving keteniminium species.^34^

## Supplementary Material

Supplementary information
