# Supplementary material for: Divergent ynamide reactivity in the presence of azides – an experimental and computational study
Source: Chem Sci. 2016 Jun 10;7(9):6032–40. doi: 10.1039/c6sc01945e (PMC6022194; doi:10.1039/c6sc01945e)
Supplement: Supplementary file 1 [file SC-007-C6SC01945E-s001.pdf]

Electronic Supplementary Material (ESI) for Chemical Science.
This journal is © The Royal Society of Chemistry 2016

Supporting Information

Divergent Ynamide Reactivity in the Presence of Azides – An Experimental and Computational Study

Veronica Tona,[†] Stefan A. Ruider,[†] Martin Berger, Saad Shaaban,[‖] Mohan Padmanaban,[‖] Lan-Gui Xie, Leticia González and Nuno Maulide **

Faculty of Chemistry, Institute of Organic Chemistry, University of Vienna

Währingerstraße 38, 1090 Vienna, Austria

nuno.maulide@univie.ac.at

Table of Contents

1- GENERAL INFORMATION S3

2- OPTIMIZATION CONDITIONS FOR THE OXAZOLIDINONE REARRANGEMENT ...S3

3- GENERAL PROCEDURE FOR THE SYNTHESIS OF OXAZOLIDINE-2,4-ONES (3A-L) S4

4- OPTIMIZATION REACTION CONDITIONS FOR YNAMIDE DIMERIZATION S10

5- GENERAL PROCEDURE FOR THE SYNTHESIS OF BIS(OXAZOLIDIN-2-ONE) (5A-H) S11

6- PROCEDURE FOR THE SYNTHESIS OF 1-PHENETHYL-7-PHENYLTETRAHYDRO-1H,5H-IMIDAZO[1,2-C]OXAZOL-5-ONE (7A) S15

7- PROCEDURE FOR THE SYNTHESIS OF FULLY-SUBSTITUTED PYRIDINE:...........S16

9- SPECTRA: S18

10- X-RAX ANALYSIS S51

11- REFERENCES: S55

1- General Information

All glassware was oven dried at 100 ºC before use. All solvents were distilled from appropriate drying agents prior to use. All reagents were used as received from commercial suppliers unless otherwise stated. Neat infra-red spectra were recorded using a Perkin-Elmer Spectrum 100 FT-IR spectrometer. Wavenumbers (υ 1/λ) are reported in cm^{-1}. Mass spectra were obtained using a Finnigan MAT 8200 or (70 eV) or an Agilent 5973 (70 eV) spectrometer, using electrospray ionization (ESI) All ^{1}H-NMR and ^{13}C-NMR experiments were recorded using Bruker AV-400, spectrometers at 300 K. Chemical shifts (δ) are quoted in ppm and coupling constants (J) are quoted in Hz. The 7.27 ppm resonance of residual $CHCl_3$ for proton spectra and 77.16 ppm resonance for carbon spectra were used as internal references. ^{1}H NMR splitting patterns were designated as singlet (s), doublet (d), triplet (t), quartet (q) or combinations thereof, splitting patterns that could not be interpreted were designated as multiplet (m). Reaction progress was monitored by thin layer chromatography (TLC) performed on aluminum plates coated with kieselgel F254 with 0.2 mm thickness. Visualization was achieved by a combination of ultraviolet light (254 nm) and acidic potassium permanganate or phosphomolybdic acid. Flash column chromatography was performed using silica gel 60 (230-400 mesh, Merck and co.).

All the Ynamide were prepared according the general procedures in the literatures [1].

2- Optimization conditions for the oxazolidinone rearrangement

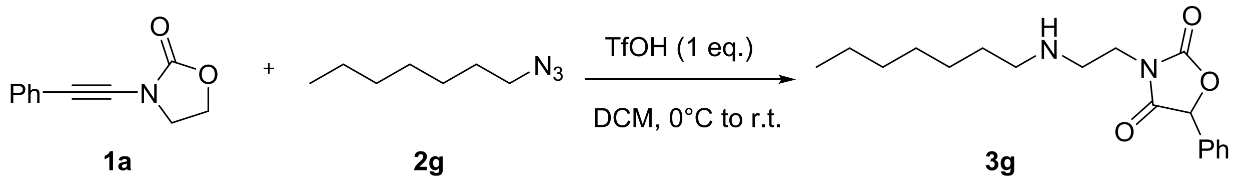

1a **2g** **3g**

Entry	Eq.**1**	Eq.**2**	Solvent	Conditions	Quench	Yield% (Conversion%[a])
1[b]	1.0	2.0	DCM	0°C 2h then 40°C overnight	-	0
2	1.0	2.0	DCM	0°C 2h then 40°C for 5h	-	(80)
3	1.0	2.0	DCE	0°C 2h then 40°C overnight	-	(75)

4	1.0	2.0	CH_3CN	0°C 2h then 40°C 5h	-	Complex mixture
5	1.0	2.0	Toluene	0°C 2h then 40°C 5h	-	Complex mixture
6	1.0	2.0	DCM	0°C 2h then 40°C 16h	$H_2$0 30′ then NaOH/Brine	57
7[c]	1.0	2.0	DCM	0°C 2h then rt 12h	$H_2$0 30′ then NaOH/Brine	63
8[c]	1.0	3.0	DCM	0°C 2h then rt 12h	$H_2$0 30′ then NaOH/Brine	55
9[c]	1.0	5.0	DCM	0°C 2h then rt 12h	$H_2$0 30′ then NaOH/Brine	62
**10[c]**	**2.0**	**1.0**	**DCM**	**0°C 2h then rt 12h**	**$H_2$0 30′ then NaOH/Brine**	**78**
11[c]	1.5	1.0	DCM	0°C 2h then rt 12h	$H_2$0 30′ then NaOH/Brine	71
12[c]	2.0	1.0	DCM	0°C 2h then rt 12h	NaOH/Brine	30

[a].Conversion of the starting material monitored through ^{1}H NMR spectroscopy. [b]. Reaction carried out in absence of TfOH. [c]. Reaction carried out in presence of 2-azidoethylbenzene (**2a**) instead of azide **2g**.

3- General procedure for the synthesis of oxazolidine-2,4-ones (3a-l)

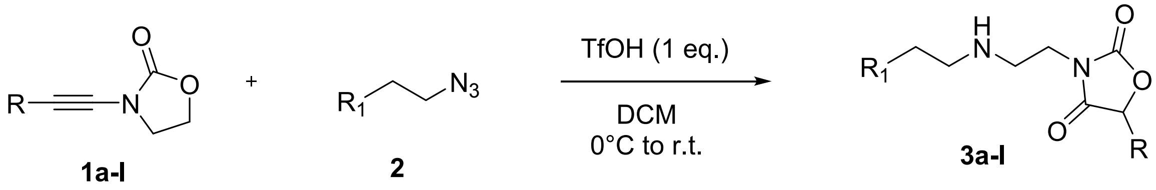

A flame dried Schlenk was charged with ynamide **1** (2.0 equiv., 0.6 mmol). The flask was evacuated and backfilled with Ar, then 0.5 ml of DCM were added and the solution was cooled to 0 °C. The azide **2** (1.0 equiv., 0.3 mmol) was added as a solution in 1 ml of DCM, then TfOH (1.0 equiv., 0.3 mmol, 27µl) was added and the mixture was slowly warmed to r.t. After stirring for 13 h, 2 ml of water were added. After 1 h of stirring, the mixture was washed with solution of NaOH 1M and brine (1:1) and extracted (3x) with DCM, dried over Na_2SO_4 and evaporated under vaccum. Purification through column chromatography using DCM to DCM/DMA (5/1) (DMA= Dichloromethane: Methanol: Ammonia / 9:1:0.15) in DCM.

3-(2-(Phenethylamino)ethyl)-5-phenyloxazolidine-2,4-dione (3a)

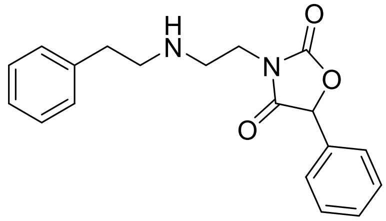

76 mg, 78% yield, yellow semisolid. **^{1}H NMR (400 MHz, $CDCl_3$)** δ = 7.42-7.38 (m, 5H), 7.28-7.23 (m, 2H), 7.19-7.13 (m, 3H), 5.63 (s, 1H), 3.67-3.63 (m, 2H), 2.91-2.83 (m, 2H), 2.70 (t, *J* = 6.9 Hz) ppm. **^{13}C**

NMR (100 MHz, CDCl$_3$) δ = 171.6, 155.6, 139.9, 131.9, 129.9, 129.2, 128.8, 128.6, 126.6, 126.3, 80.4, 50.4, 46.2, 40.0, 36.5. **HRMS (ESI)**: [M+H]$^+$ calculated for $C_{19}H_{20}O_3N_2$ *m/z*= 325.1547, found *m/z*= 325.1551. **ATR-FTIR (cm^{-1})**: 3028, 2941, 2829, 1816, 1737, 1603, 1558, 1540, 1521, 1495, 1115, 1025, 750, 701, 631.

3-(2-(Phenethylamino)ethyl)-5-(m-tolyl)oxazolidine-2,4-dione (3b)

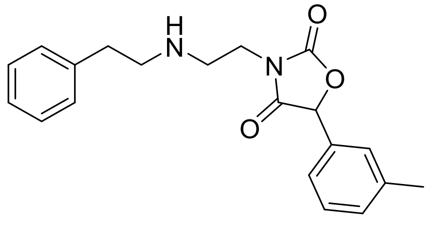

63 mg, 62% yield, orange semisolid. **^{1}H NMR (400 MHz, CDCl$_3$)** δ = 7.33-7.18 (m, 9H), 5.65 (s, 1H), 3.73-3.69 (m, 2H), 2.95-2.89 (m, 4H), 2.76 (t, *J*= 7 Hz, 2H), 2.40 (s, 3H) ppm. **^{13}C NMR (100 MHz, CDCl$_3$)** δ = 171.7, 155.6, 139.9, 139.0, 131.8, 130.7, 129.1, 128.8, 128.6, 127.1, 126.3, 123.6, 80.5, 50.5, 46.3, 40.0, 36.5, 21.5 ppm. **HRMS (ESI)** [M+H$^+$] calculated for $C_{20}H_{22}O_3N_2$*m/z*=319.1703, found *m/z* = 319.1700. **ATR-FTIR (cm^{-1})**: 3334, 3059, 3026, 2921, 2851, 1813, 1731, 1606, 1440, 1409, 1331, 1161, 1109, 1033, 911, 759, 699.

3-(2-(Phenethylamino)ethyl)-5-(p-tolyl)oxazolidine-2,4-dione (3c)

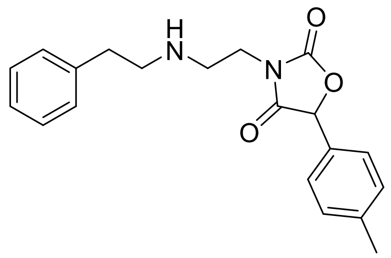

54 mg, 53% yield, orange liquid. **^{1}H NMR (400 MHz, CDCl$_3$)** δ = 7.26-7.10 (m, 9H), 5.57 (s, 1H), 3.64-3.60 (m, 2H), 2.88-2.82 (m, 4H), 2.70-2.66 (m, 2H), 2.31 (s, 3H) ppm. **^{13}C NMR (100 MHz, CDCl$_3$)** δ = 171.8, 155.7, 140.1, 140.0, 129.9, 129.0, 128.9, 128.6, 126.7, 126.3, 80.6, 50.5, 46.3, 40.0, 36.5, 21.4 ppm. **HRMS (ESI)** [M+H$^+$] calculated for $C_{20}H_{22}O_3N_2$ *m/z*=339.1703, found *m/z*= 339.1703. **ATR-FTIR (cm^{-1}):** 3028, 2925, 2846, 1816, 1739, 1472, 1442, 1114, 763, 700.

5-(4-Chlorophenyl)-3-(2-(phenethylamino)ethyl)oxazolidine-2,4-dione (3d)

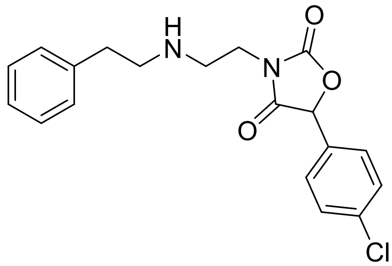

67 mg, 63% yield, yellow semisolid. **^{1}H NMR (400 MHz, CDCl$_3$)** δ = 7.42-7.37 (m, 4H), 7.32-7.28 (m, 2H), 7.23-7.16 (m, 3H), 5.63 (s, 1H), 3.71-3.66 (m, 2H), 2.94-2.86 (m, 4H), 2.73 (t, *J*= 6.9 Hz, 3H) ppm. **^{13}C NMR (100 MHz, CDCl$_3$)** δ = 171.3, 155.3, 139.8, 136.0, 130.4, 129.4, 128.8, 128.6, 127.9, 126.3, 79.6, 50.4, 46.1, 40.0, 36.4 ppm. **HRMS (ESI)** [M+H$^+$] calculated for $C_{19}H_{19}O_3N_2Cl$ *m/z*=359.1157, found *m/z*= 359.1157. **ATR-FTIR (cm^{-1})**: 3062, 2979, 2848, 1819, 1736, 1599, 1541, 1522, 1494, 1442, 1409, 1330, 1296, 1167, 1111, 1046, 1014, 800, 761, 700.

5-(4-Fluorophenyl)-3-(2-(phenethylamino)ethyl)oxazolidine-2,4-dione (3e)

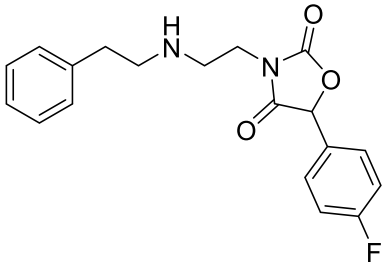

57 mg, 56% yield, white semisolid. **^{1}H NMR (400 MHz, CDCl$_3$)** δ = 7.45-7.41 (m, 2H), 7.31-7.27 (m, 2H), 7.22-7.01 (m, 5H), 5.64 (s, 1H), 3.73-3.64 (m, 2H), 2.94-2.86 (m, 4H), 2.73 (t, *J* = 8 Hz, 2H) ppm. **^{13}C NMR (100 MHz, CDCl$_3$)** δ = 171.4, 163.6 (d, *J*= 249.7 Hz), 155.3, 139.8, 128.7, 128.6 (d, *J*= 8.6 Hz), 128.5, 127.7 (d, *J*= 3.3 Hz), 126.2, 116.2 (d, *J*= 22.0 Hz), 79.7, 50.3, 46.1, 39.9, 36.4 ppm. **HRMS (ESI)** [M+H$^+$] calculated for $C_{19}H_{19}O_3N_2F$ *m/z*=343.1452, found *m/z* = 343.1439. **ATR-FTIR (cm^{-1})**: 3309, 3027, 2930, 2849, 1816, 1735, 1668, 1604, 1510, 1441, 1411, 1337, 1226, 1160, 1108, 1031, 1013, 911, 843, 808, 760, 700, 612.

5-(4-Bromophenyl)-3-(2-(phenethylamino)ethyl)oxazolidine-2,4-dione (3f)

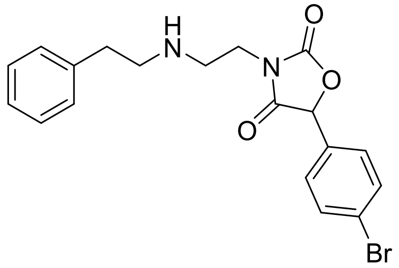

80 mg, 66% yield, yellow semisolid. **^{1}H NMR (400 MHz, CDCl$_3$)** δ = 7.57-7.55 (m, 2H), 7.34-7.27 (m, 4H), 7.21-7.16 (m, 3H), 5.62 (s, 1H), 3.70-3.66 (m, 2H), 2.94-2.86 (m, 4H), 2.73 (t, *J*= 6.9 Hz, 2H) ppm. **^{13}C NMR (100 MHz, CDCl$_3$)** δ = 171.2, 155.3, 139.8, 132.4, 130.9, 128.8, 128.6, 128.1, 126.3, 124.2, 79.6, 50.4, 46.1, 40.0, 36.4 ppm. **HRMS (ESI)** [M+H$^+$] calculated for $C_{19}H_{19}O_3N_2Br$ *m/z*=403.0652, found *m/z* = 403.0650. **ATR-FTIR (cm^{-1})**: 3214, 3027, 2936, 2827, 1815, 1736, 1669, 1633, 1590, 1558, 1541, 1524, 1498, 1440, 1406, 1332, 1107, 1071, 1044, 1030, 1009, 913, 857, 732, 698.

3-(2-(Heptylamino)ethyl)-5-phenyloxazolidine-2,4-dione (3g)

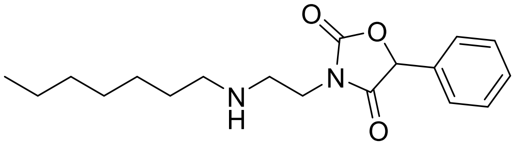

49 mg, 52% yield, white solid. **^{1}H NMR (400 MHz, CDCl$_3$)** δ = 7.45-7.41 (m, 5H), 5.71 (s, 1H), 3.72-3.67 (m, 2H), 2.92 (dt, *J*= 6.2, 1.2 Hz, 2H), 2.42 (dt, *J*= 7.1 Hz, 2H), 1.42-1.39 (m, 2H), 1.29-1.25 (m, 8H), 1.12 (bs, 1H), 0.87 (t, *J*= 7.0 Hz, 3H) ppm. **^{13}C NMR (100 MHz, CDCl$_3$)** δ = 171.7, 155.6, 131.9, 129.9, 129.2, 126.6, 80.5, 49.5, 46.5, 40.1, 31.9, 30.1, 29.3, 27.3, 22.7, 14.2 ppm. **HRMS (ESI)**: [M+H]$^+$ calculated for $C_{18}H_{26}O_3N_2$ *m/z*= 319.2016, found *m/z*= 319.2016. **ATR-FTIR (cm^{-1})**: 2924, 2854, 1813, 1735, 1444, 1415, 1342, 1132, 1076, 738, 631

5-(4-Fluorophenyl)-3-(2-(heptylamino)ethyl)oxazolidine-2,4-dione (3h)

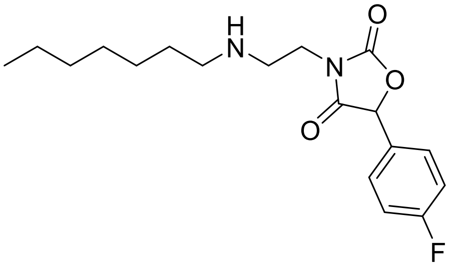

57 mg, 57% yield, white semisolid. **^{1}H NMR (400 MHz, CDCl$_3$)** δ = 7.46-7.43 (m, 2H), 7.13-7.08 (m, 2H), 5.69 (s, 1H), 3.74-3.66 (m, 2H), 2.91 (dt, *J* = 6.2, 2.4 Hz, 2H), 2.58 (t, *J*= 7.1 Hz, 2H), 1.42-1.38 (m, 2H), 1.29-1.25 (m, 8H), 0.87 (t, *J*= 6.8 Hz, 3H) ppm. **^{13}C NMR (100 MHz, CDCl$_3$)** δ = 171.6, 163.7 (d, *J*= 249.6 Hz), 155.4, 128.8 (d, *J*= 8.6 Hz), 127.9 (d, *J*= 3.3 Hz), 116.3 (d, *J*= 22.0 Hz), 79.8, 49.5, 46.5, 40.2, 31.9, 30.3, 29.3, 27.3, 22.7, 14.2 ppm. **HRMS (ESI)** [M+H$^+$] calculated for $C_{18}H_{25}O_3N_2F$ *m/z*=337.1922, found *m/z*= 337.1909. **ATR-FTIR (cm^{-1})**: 3545, 3425, 2954, 2928, 2854, 2414, 1816, 1739, 1713, 1698, 1627, 1592, 1541, 1510, 1470, 1441, 1412, 1332, 1274, 1223, 1189, 1157, 1144, 1101, 1041, 1016, 930, 840, 814, 763, 742, 705.

3-(2-(Phenethylamino)ethyl)-5-(4-(trifluoromethyl)phenyl)oxazolidine-2,4-dione (3i)

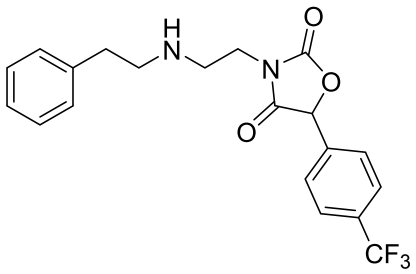

60 mg, 76% yield, yellowish oil. **^{1}H NMR (400 MHz, CDCl$_3$)** δ = 7.69 (bd, *J* = 8.3 Hz, 2H), 7.60 (bd, *J*= 8.3 Hz, 2H), 7.31-7.25 (m, 2H), 7.20 (tt, *J*= 7.3, 2.3 Hz, 1H), 7.17-7.12 (m, 2H), 5.70 (s, 1H), 3.74-3.61 (m, 2H), 2.96-2.81 (m, 4H), 2,71 (t, *J*= 6.8 Hz, 2H) ppm. **^{13}C NMR (100 MHz, CDCl$_3$)** δ = 170.9, 155.2, 139.9, 135.7, 132.1 (d, *J* = 32.5 Hz), 128.9, 128.6, 126.7, 126.4, 126.2 (q, *J*= 3.8 Hz), 123.8 (app. d, *J*= 272.8 Hz), 79.3, 50.4, 46.1, 40.1, 36.5 ppm. **HRMS (ESI)** [M + H]$^+$ calculated for $C_{20}H_{19}F_3N_2O_3$ *m/z* = 393.1421, found *m/z*= 393.1422. **ATR-FTIR (cm^{-1})**: 2925, 1819, 1739, 1443, 1413, 1325, 1167, 1125, 1068, 1017, 843, 762, 701.

Methyl 4-(2,4-dioxo-3-(2-(phenethylamino)ethyl)oxazolidin-5-yl)benzoate (3j)

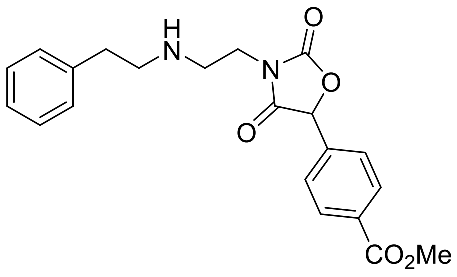

57 mg, 74% yield, orange solid. **^{1}H NMR (400 MHz, CDCl$_3$)** δ = 8.09 (bd, *J* = 8.5 Hz, 2H), 7.54 (bd, *J*= 8.5 Hz, 2H), 7.31-7.25 (m, 2H), 7.20 (tt, *J*= 7.3, 2.1 Hz, 1H), 7.17-7.12 (m, 2H), 5.71 (s, 1H), 3.93 (s, 3H), 3.75-3.61 (m, 2H), 2.96-2.81 (m, 4H), 2.71 (t, *J*= 7.0 Hz, 2H) ppm. **^{13}C NMR (100 MHz, CDCl$_3$)** δ = 171.0, 166.4, 155.3, 139.9, 136.5, 131.5, 130.4, 128.9, 128.6, 126.3, 126.3, 79.6, 53.5, 50.4, 46.1, 40.1, 36.4. **HRMS (ESI)** [M + H]$^+$ calculated for $C_{21}H_{22}N_2O_5$ *m/z*= 383.1601, found *m/z*= 383.1601. **ATR-FTIR (cm^{-1})**: 2951, 1819, 1739, 1440, 1412, 1282, 1192, 1109, 1048, 1018, 756, 720, 701.

3-((*S*)-3-Methyl-1-(phenethylamino)butan-2-yl)-5-phenyloxazolidine-2,4-dione (3k)

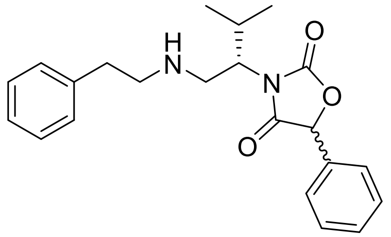

A solution of ynamide **1k** (2.0 equiv., 0.2 mmol, 46 mg) in dry DCM (0.4 mL) was cooled to 0 °C, and the azide **2a** (1.0 equiv., 0.1 mmol, 15 mg) was added (neat addition and after washing of the vial with 0.1

mL dry DCM), followed by TfOH (1.0 equiv., 0.1 mmol, 9.0 µL). After stirring for 13 h at r.t., the mixture was quenched with 2 mL water. After addition of brine (10 mL) and 1 N NaOH (10 mL), the aqueous layer was extracted with DCM (3 x 10 mL). After drying the combined organic layers with $MgSO_4$, the mixture was concentrated under reduced pressure. ^{1}HNMR of the crude mixture revealed a diastereomeric ratio of 85:15. Purification of the crude product by flash chromatography (3-12% DMA-Mix in DCM) gave 26.5 mg (72%) of the product as a white solid with a d.r. of 1:1. Attempts to separate the diastereomers by LC-MS or preparative HPLC were not successful. The following analytical data describes the 1:1 mixture of diastereomers. **^{1}H NMR** (600 MHz, $CDCl_3$): 7.48-7.41 (m, 10H), 7.31-7.26 (m, 4H), 7.22-7.16 (m, 4H), 7.11 (bd, *J* = 7.6 Hz, 2H), 5.55 (s, 1H), 5.50 (s, 1H), 3.76-3.72 (m, 2H), 3.22 (bt, *J*= 12.2 Hz, 1H), 3.17 (bt, *J*= 12.2 Hz, 1H), 2.94-2.86 (m, 4H), 2.78-2.70 (m, 4H), 2.65 (t, *J*= 6.8 Hz, 2H) 2.36-2.27 (m, 2H), 1.00 (dd, *J*= 6.8, 1.2 Hz, 6H), 0.90 (dd, *J*= 6.5, 5.1 Hz, 6H). **^{13}C NMR** (100 MHz, $CDCl_3$): 172.5, 172.3, 155.8, 155.6, 140.1, 140.0, 132.4, 132.3, 129.9, 129.8, 129.2, 129.1, 128.9, 128.8, 128.6 (2C), 126.8, 126.7, 126.2 (2C), 79.8, 79.7, 60.0, 59.8, 50.6, 50.6, 47.5, 47.3, 36.6, 36.5, 28.4, 28.3, 20.4, 20.4, 20.3, 20.2. **HRMS (ESI)** $[M + H]^+$ calculated for $C_{22}H_{26}N_2O_3$ *m/z*= 367.2016, found *m/z*= 367.2015. **ATR-FTIR (cm^{-1})**: 2964, 1810, 1734, 1455, 1404, 1375, 1176, 1128, 1049, 1027, 763, 699.

5-Cyclohexyl-3-(2-(phenethylamino)ethyl)oxazolidine-2,4-dione (3l)

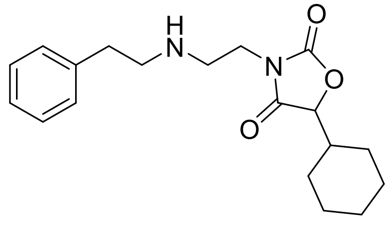

40 mg, 40% yield, white solid. **^{1}H NMR (400 MHz, $CDCl_3$)** δ = 7.21-7.17 (m, 2H,), δ 7.12-7.07 (m, 3H), δ 4.57 (d, *J*= 3.8 Hz, 1H), δ 3.63-3.59 (m, 2H), δ 2.87 (t, *J*= 6.6 Hz, 4H), δ 2.74 (t, *J*= 7.0 Hz, 2H), δ 1.99-1.93 (m, 1H), 1.81-1.75 (m, 3H,), 1.68 (d, *J*= 12.2 Hz, 1H), 1.58 (d, *J*= 9.6 Hz, 1H), 1.32-1.16(m, 6H) ppm. **^{13}C NMR (100 MHz, $CDCl_3$)** δ = 172.9, 156.0, 139.9, 128.9, 128.6, 126.3, 83.6, 50.5, 46.5, 39.6 (2C), 36.5, 28.5, 26.0, 25.8 (2C), 25.7 ppm **HRMS (ESI)** $[M + H]^+$ calculated for $C_{19}H_{26}N_2O_3$ *m/z*= 331.2016, found *m/z*= 331.2005. **ATR-FTIR (cm^{-1})**: 2925, 2853, 1812, 1731, 1445, 1414, 1339, 1119, 764, 701, 631, 531.

3-(2-((4-Fluorophenethyl)amino)ethyl)-5-phenyloxazolidine-2,4-dione (3m)

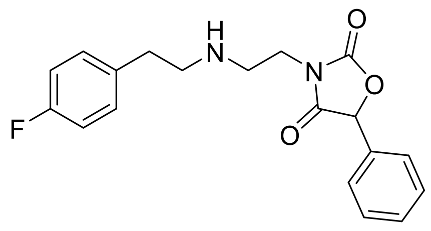

34 mg, 50% yield (0.2 mmol scale), yellow semisolid. **^{1}H NMR (400 MHz, $CDCl_3$)** δ = 7.43 (s, 5H), 7.12-7.09 (m, 2H), 6.98-6.94 (m, 2H), 5.68 (s, 1H), 3.71-3.66 (m, 2H), 2.92 (dt, *J*= 6.1, 2.4 Hz, 2H), 2.86-2.83 (m, 2H), 2.69 (t, *J* = 6.9 Hz) ppm. **^{13}C NMR (100 MHz, $CDCl_3$)** δ = 171.7, 161.6 (d, *J* = 243.9 Hz), 155.6, 135.6 (d, *J*= 3.2 Hz), 131.9, 130.3, 130.2, 130.0, 129.2, 126.5, 115.5, 115.3, 80.5, 50.5, 46.3, 40.0, 35.8. **HRMS (ESI)**: $[M+H]^+$ calculated for $C_{19}H_{20}O_3N_2F$ *m/z*= 343.1452, found *m/z*= 343.1455. **ATR-FTIR (cm^{-1})**:

3356, 2922, 2851, 1817, 1737, 1509, 1442, 1413, 1347, 1220, 1158, 1113, 1046, 1017, 825, 762, 705, 643.5.

3-(2-((4-Methylphenethyl)amino)ethyl)-5-phenyloxazolidine-2,4-dione (3n)

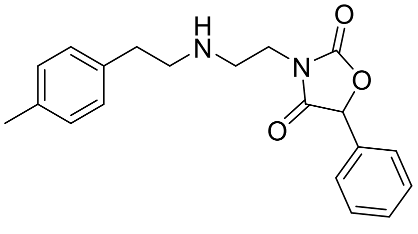

57 mg, 84% yield, green semisolid. **^{1}H NMR (400 MHz, CDCl$_3$)** δ = 7.46-7.41 (m, 5H), 7.10 (d, *J*=8.0 Hz, 2H), 7.05 (d, *J*=8.0 Hz, 2H), 5.76 (s, 1H), 3.72-3.64 (m, 2H), 2.91 (dt, *J*= 6.2, 2.1 Hz, 2H), 2.87-2.84 (m, 2H), 2.69 (t, *J*= 7.0 Hz, 1H),2.31 (s, 1H) ppm. **^{13}C NMR (100 MHz, CDCl$_3$)** δ = 171.7, 136.8, 135.8, 132.0, 130.2, 130.0, 129.3, 129.2, 128.8, 126.6, 80.5, 50.6, 46.3, 40.0, 36.0, 21.1. **HRMS (ESI)**: $[M+H]^+$ calculated for $C_{20}H_{22}O_3N_2$ *m/z*= 339.1703, found *m/z*= 347.2334. **ATR-FTIR (cm^{-1})**: 3838, 3750, 3673, 3649, 3617, 2922, 2851, 1816, 1736, 1558, 1541, 1515, 1495, 1474, 1442, 1410, 1347, 1222, 1166, 1114, 1040, 920, 810, 761, 702, 645.

5-Heptyl-3-(2-(phenethylamino)ethyl)oxazolidine-2,4-dione (3o)

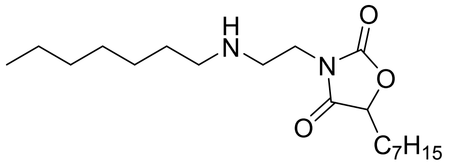

35 mg, 37% yield, white solid **^{1}H NMR (400 MHz, CDCl$_3$)** δ 4.76 (dd, *J* = 7.3, 4.4 Hz, 1H), 3.65-3.61 (m, 2H), 2.87 (t, *J* = 6.2 HZ, 2H), 2.58 (t, *J* = 7.1 HZ, 2H), 2.03-1.94 (m, 1H), 1.85-1.76 (m, 1H), 1.46-1.40 (m, 4H), 1.33-1.26 (m, 16H), 1.04 (bs, 1H), 0.87 (t, *J* = 7.1 Hz, 6H)**; ^{13}C NMR (100 MHz, CDCl$_3$)** δ 173.53, 155.87, 79.85, 49.56, 46.77, 39.95, 31.97, 31.79, 31.08, 30.28, 29.36, 29.10, 29.07, 27.35, 24.13, 22.76, 22.71, 14.21, 14.17. **ESI-MS**: calculated $[M+H]^+$for $C_{19}H_{35}O_3N_2$: 341.2799, found: 341.2803. **ATR-FTIR (cm^{-1})**: 2924, 2854, 1813, 1735, 1444, 1415, 1342, 1132, 1076, 738, 631.

4- Optimization reaction conditions for ynamide dimerization

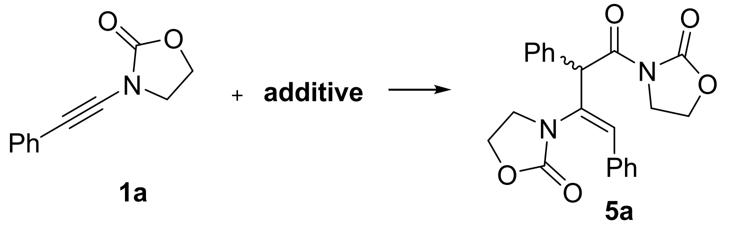

	additive	eq. additive	TfOH (eq.)	Solvent	Concentration (M)	Temperature	Time	Yield **5a**	Notes
1	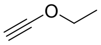	2	1.1	DCM	0.05	-40 to 50°C	16h	35%[a]	-
2	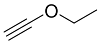	1.2	0.5	DCM	0.05	-40 to 50°C	16h	Traces	-
3	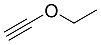	1.2	2	DCM	0.05	-40 to 50°C	16h	10%[a]	-
4	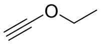	2	1.1	DCM	0.1	-40 to 50°C	1h	28%[a]	-
5	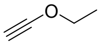	2	1.1	DCM	0.25	-40 to 50°C	1h	34%[a]	-
6	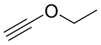	2	1.1	DCM	0.5	-40 to 50°C	1h	18%	-
7	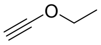	2	TFA	DCM	0.05	-40 to 50°C	-	-	Starting material
8	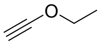	2	MsOH	DCM	0.05	-40 to 50°C	-	-	Starting material
9	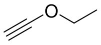	2	Tf_2NH	DCM	0.25	-40 to 50°C	1h	3%[a]	-
10	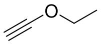	1.2	1.1	THF	0.05	-40 to 50°C	1h	-	Complex mixture
11	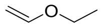	2	1.1	DCM	0.05	-40 to 50°C	1h	0%[a]	Polymerization of the ether
12	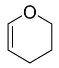	2	1.1	DCM	0.05	-40 to 50°C	1h	0%[a]	Polymerization of the ether
**13**	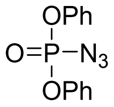	**0.5**	**0.5**	**DCM**	**0.2**	**0°C to r.t.**	**13h**	**80%**	**-**
14	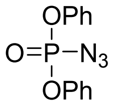	0.1	0.5	DCM	0.2	0°C to r.t.	13h	30%	-

15	O=PPh$_3$	0.5	0.5	DCM	0.2	0°C to r.t.	13h	-	-
16	O=P(OPh)$_3$	0.5	0.5	DCM	0.2	0°C to r.t.	13h	30%	-

[a]. Measured through ^{1}H NMR. b. Reverse addition: a solution of ynamide and azide in 1ml dry DCM was added at 0°C to a solution of TfOH in 0.5 ml of dry DCM.

5- General procedure for the synthesis of bis(oxazolidin-2-one) (5a-h)

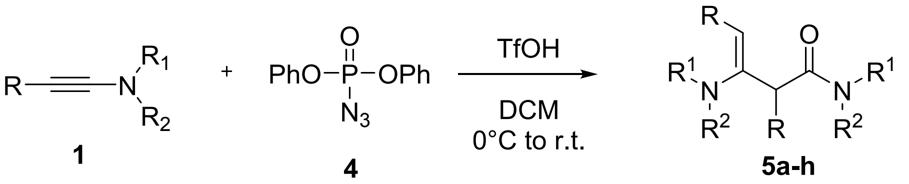

A flame-dried Schlenk was charged with ynamide **1** (2.0 equiv., 0.6 mmol). The flask was evacuated and backfilled with Ar, then 0.5 ml of DCM were added and the solution was cooled to 0 °C. Then dppa **4** (1.0 equiv., 0.3 mmol, 83 mg) was added as a solution in 1 ml of DCM, followed by the addition of TfOH (1.0 equiv.,0.3 mmol, 27 µl) and the mixture was slowly warmed to r.t. After stirring for 13 h, the mixture was washed with 10 ml of a solution of NaOH 1M and brine (1:1), extracted with DCM (3x) and dried over Na_2SO_4. Purification through column chromatography using DCM to DCM/DMA (9:1) led to desired dimers **5a-h**.

***(Z)*-3,3'-(1-Oxo-2,4-diphenylbut-3-ene-1,3-diyl)bis(oxazolidin-2-one) (5a)**

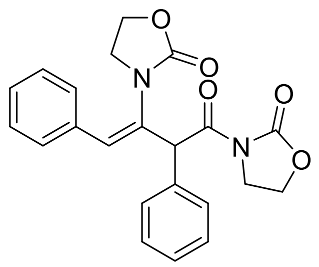

95 mg, 81% yield, orange semisolid. **^{1}H NMR (400 MHz, CDCl$_3$)** δ = 7.48-7.45 (m, 2H), 7.40-7.28 (m, 5H), 7.23–7.21 (m, 3H), 6.61 (s, 1H), 6.00 (s, 1H), 4.43-4.37 (m, 1H), 4.34-4.30 (m, 1H), 4.25-4.21 (m, 1H), 4.20-4.13 (m, 1H), 4.12-4.05 (ddd, *J*= 10.8, 9.4, 6.9 Hz, 1H), 4.02-3.96 (ddd, *J*= 10.9, 9.3, 6.7 Hz, 1H) ppm. **^{13}C NMR (100 MHz, CDCl$_3$)** δ = 171.5, 156.8, 152.6, 135.4, 135.1, 135.0, 130.2, 129.0, 128.7, 128.4, 128.2, 128.0, 127.5, 63.1, 61.8, 53.4, 45.8, 43.0 ppm. **HRMS (ESI)** [M+Na$^+$] calculated for $C_{22}H_{20}O_5N_2$ *m*/*z*=415.1264, found *m*/*z*=415.1272. **ATR-FTIR (cm^{-1})**: 3057, 2987, 2917, 1778, 1748, 1694, 1406, 1264, 1220, 1110, 1039, 730, 699.

***(Z)*-3,3'-(1-Oxo-2,4-di-m-tolylbut-3-ene-1,3-diyl)bis(oxazolidin-2-one) (5b)**

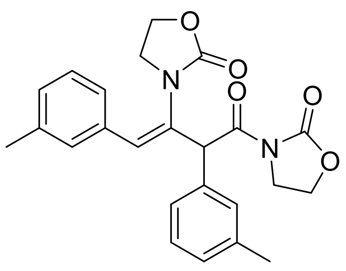

89 mg, 71% yield, white solid. **^{1}H NMR (400 MHz, CDCl$_3$)** δ = 7.27-7.03 (m, 8H), 6.56 (s, 1H), 5.96 (s, 1H), 4.40-3.96 (m, 6H), 3.60-3.54 (m, 1H), 3.48-3.42 (m, 1H), 2.36 (s, 3H), 2.29 (s, 3H) ppm. **^{13}C NMR (100 MHz, CDCl$_3$)** δ = 171.4, 156.7, 152.4, 138.5, 138.1, 135.1, 134.9, 134.7, 130.8, 129.0, 128.9, 128.7, 128.6, 128.4, 127.4, 126.9, 125.2, 77.2, 62.9, 61.6, 53.2, 45.7, 42.9, 21.5, 21.3. **HRMS (ESI)** [M+Na$^+$] calculated for $C_{24}H_{24}O_5N_2$ *m/z*=443.1577, found *m/z* = 443.1576. **ATR-FTIR (cm^{-1})**: 2958, 2918, 1773, 1743, 1691, 1478, 1403, 1385, 1361, 1290, 1242, 1200, 1110, 1092, 1036, 1000, 780, 733, 710, 699.

***(Z)*-3,3'-(2,4-Bis(4-bromophenyl)-1-oxobut-3-ene-1,3-diyl)bis(oxazolidin-2-one) (5c)**

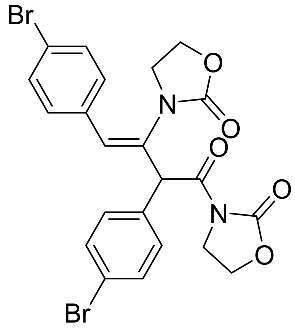

81 mg, 49% yield, yellowish semisolid. **^{1}H NMR (400 MHz, CDCl$_3$)** δ = 7.52 (d, 2H, *J*= 8.4 Hz), 7.45-7.43 (m, 2H), 7.33 (d, 2H, *J*= 8.4 Hz), 7.11 (d, 2H, *J*= 8.4 Hz), 6.55 (s, 1H), 5.89 (s, 1H), 4.42-4.17 (m, 4H), 4.11-3.94 (m, 2H), 3.60-3.54 (m, 1H), 3.48-3.42 (m, 1H) ppm. **^{13}C NMR (100 MHz, CDCl$_3$)** δ = 170.9, 156.7, 152.6, 135.8, 133.8, 132.3, 132.0, 131.9, 129.9, 126.3, 122.6, 122.1, 63.1, 61.9, 52.8, 45.8, 43.0 ppm. **HRMS (ESI)** [M+Na$^+$] calculated for $C_{22}H_{18}O_5N_2Br_2$ *m/z*=572.9454, found *m/z*= 572.9451. **ATR-FTIR (cm^{-1})**: 3528, 3055, 2984, 2918, 2854, 1773, 1744, 1693, 1587, 1484, 1406, 1385, 1363, 1218, 1109, 1072, 1037, 1009, 835, 814, 758, 731.

***(Z)*-3,3'-(1-Oxo-2,4-di-p-tolylbut-3-ene-1,3-diyl)bis(oxazolidin-2-one) (5d)**

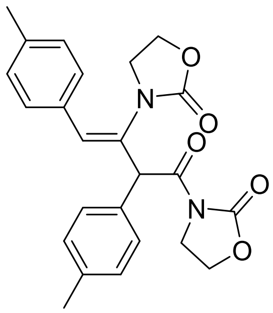

91 mg, 72% yield, brownish semisolid. **^{1}H NMR (400 MHz, CDCl$_3$)** δ = 7.34 (d, *J*= 8.1 Hz, 2H), 7.18 (d, *J*= 8.0 Hz, 2H), 7.13 (d, *J*= 8.1 Hz, 2H), 7.09 (d, *J*= 8.1 Hz, 2H), 6.54 (s, 1H), 5.96 (s, 1H), 4.40-4.36 (m, 1H), 4.32-4.28 (m, 1H), 4.20-4.16 (m, 1H), 4.09-4.05 (m, 1H), 4.00-4.95 (m, 1H), 3.60-3.56 (m, 1H), 3.49-3.45 (m, 1H), 2.35 (s, 3H), 2.31 (s, 3H) ppm. **^{13}C NMR (100 MHz, CDCl$_3$)** δ = 171.6, 156.8, 152.6, 138.0, 137.8, 134.6, 132.1, 131.9, 130.1, 129.7, 129.3, 128.2, 127.2, 63.1, 61.8, 53.0, 45.6, 43.0, 21.4, 21.3 ppm. **HRMS (ESI)** [M+Na$^+$] calculated for $C_{24}H_{24}O_5N_2$ *m/z*=443.1577, found *m/z*= 443.1565. **ATR-FTIR (cm^{-1})**: 2920, 2250, 1745, 1692, 1512, 1478, 1404, 1386, 1363, 1219, 1112, 1038, 964, 909, 842, 758, 728, 644.

***(Z)*-3,3'-(1-oxo-2,4-bis(4-(trifluoromethyl)phenyl)but-3-ene-1,3-diyl)bis(oxazolidin-2-one) (5e)**

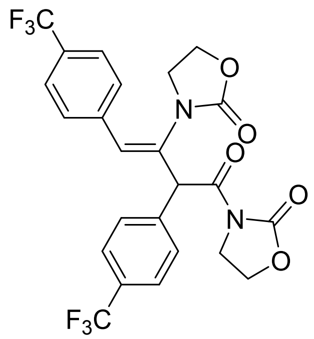

32 mg, 34% yield, brownish semisolid. **^{1}H NMR (400 MHz, $CDCl_3$)** δ = 7.62-7.46 (m, 4H), 7.31-7.26 (d, *J* = 8.4 Hz, 2H), 6.61 (s, 1H), 5.88 (s, 1H), 4.40-4.12 (m, 4H), 3.98-3.82 (m, 2H), 3.60-3.42 (m, 2H) ppm. **^{13}C NMR (100 MHz, $CDCl_3$)** δ = 170.4, 156.4, 152.5, 138.6, 138.0, 134.7, 130.4, 130.1, 129.2, 128.3, 126.1, 125.9, 125.6, 122.5, 64.8, 62.9, 61.8, 53.0, 51.5, 45.8, 42.7, 35.4 ppm. **HRMS (ESI)** [M+Na$^+$] calculated for $C_{24}H_{18}O_5N_2F_6$ *m/z*=551.1018, found *m/z*= 551.1021. **ATR-FTIR (cm^{-1})**: 2924, 1751, 1698, 1480, 1388, 1365, 1265, 1221, 1165, 1040, 963, 847, 733, 635.

***(Z)*-3,3'-(2-(4-Methoxyphenyl)-1-oxo-4-(p-tolyl)but-3-ene-1,3-diyl)bis(oxazolidin-2-one) (5f)**

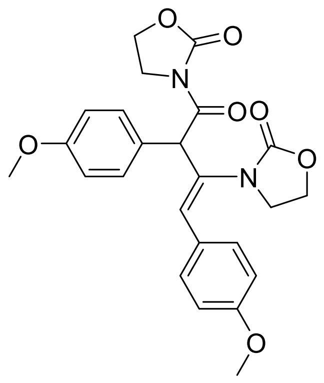

82 mg, 60% yield, pink solid. **^{1}H NMR (400 MHz, $CDCl_3$)** δ = 7.39-7.37 (m, 2H), 7.19-7.17 (m, 2H), 6.90 (d, 2H, *J*= 8.8 Hz), 6.82 (d, 2H, *J*= 8.8 Hz), 6.51 (s, 1H), 5.97 (s, 1H), 4.40-4.16 (m, 4H), 4.09-4.04 (m, 2H), 3.81 (s, 3H), 3.79 (s, 3H) ppm. **^{13}C NMR (100 MHz, $CDCl_3$)** δ = 171.8, 159.5, 159.3, 156.9, 152.6, 134.0, 131.4, 129.7, 127.5, 127.0, 126.9, 114.4, 114.1, 63.1, 61.8, 55.4, 52.5, 45.6, 43.0 ppm. **HRMS (ESI)** [M+Na$^+$] calculated for $C_{24}H_{24}O_7N_2$ *m/z*=575.1467, found *m/z*= 575.1460. **ATR-FTIR (cm^{-1})**: 2910, 2837, 1741, 1692, 1605, 1510, 1404, 1386, 1362, 1298, 1245, 1176, 1110, 1030, 963, 908, 834, 794, 759, 726, 646.

***(Z)*-3,3'-(2,4-di(cyclohex-1-en-1-yl)-1-oxobut-3-ene-1,3-diyl)bis(oxazolidin-2-one) (5g)**

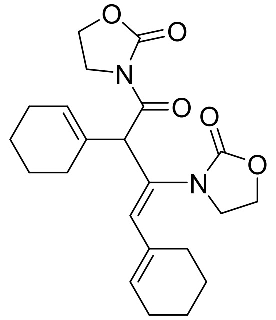

68 mg, 60% yield, pink solid. **^{1}H NMR (400 MHz, $CDCl_3$)** δ = 5.85-5.82 (s, 1H), 5.76-5.72 (s, 1H), 5.48-5.45 (s, 1H), 5.12-5.09 (s, 1H), 4.35-4.20 (m, 4H), 4.09-4.04 (m, 2H) 3.77-3.65 (m, 2H), 2.12-2.04 (m, 6H), 2.01-1.91 (m, 2H), 1.57-1.44 (m, 10H) ppm. **^{13}C NMR (100 MHz, $CDCl_3$)** δ = 171.2, 156.5, 153.0, 134.1, 133.5, 132.7, 131.6, 130.1, 129.1, 127.4, 125.7, 62.3, 61.7, 55.4, 46.1, 42.4, 35.1, 29.4, 28.6, 26.5, 25.8, 25.4, 24.6, 22.8, 22.2, 21.7, 21.1 ppm. **HRMS (ESI)** [M+Na$^+$] calculated for $C_{22}H_{28}O_5N_2$ *m/z*=423.1869, found *m/z*=423.1882. **ATR-FTIR (cm^{-1})**: 2929, 1748, 16952, 1616, 1480, 1323, 1266, 1165, 1039, 1016, 974, 847, 732, 701, 636.

***(Z)*-3-((N,4-dimethylphenyl)sulfonamido)-N-methyl-2,4-diphenyl-N-tosylbut-3-enamide (5h)**

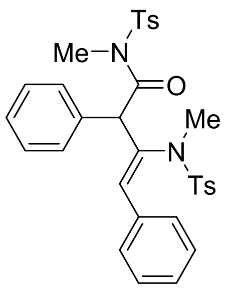

44 mg, 58% yield, yellowish solid. **^{1}H NMR (400 MHz, CDCl$_3$)** δ = 7.82-7.77 (d, *J* = 8.4 Hz, 1H), 7.55-7.51 (d, *J*= 8.6 Hz, 1H), 7.44-7.40 (d, *J*= 8.6 Hz, 1H), 7.38-7.32 (t, *J*= 9.1 Hz, 8.8 Hz, 1H), 7.27-7.20 (m, 3H), 7.19-7.15 (t, *J*= 7.7 Hz, *J*= 7.5 Hz, 1H), 7.10-7.04 (t, *J*= 6.9 Hz, *J*= 7.4 Hz, 2H), 7.03-6.95 (m, 3H), 3.49-4.45 (s, 1H), 2.71-2.65 (s, 3H), 2.69-2.65 (d, *J*= 5.5 Hz 1H), 2.50-2.43 (s, 6H), 2.40-2.36 (s, 3H) ppm. **^{13}C NMR (100 MHz, CDCl$_3$)** δ = 144.8, 143.5(2C), 136.8, 135.6(2C), 135.2, 134.4, 129.8(2C), 129.2(2C), 128.8(2C), 128.1(2C), 127.6(2C), 127.2(2C), 98.5, 38.7(2C), 37.1 (2C), 22.7, 21.5 (2C), 21.4 (2C) ppm. **HRMS (ESI)** [M+Na$^+$] calculated for $C_{33}H_{33}N_2O_5S_2$ *m/z*=611.1650, found *m/z*= 611.1648. **ATR-FTIR (cm^{-1})**: 3308, 3055, 2942, 1752, 1597, 1493, 1410, 1340, 1265, 1155, 1088, 1010, 948, 881, 786, 754, 696, 602.

3,3'-(2-butyl-1-oxooct-3-ene-1,3-diyl)bis(oxazolidin-2-one) (5i)

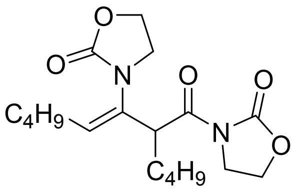

87 mg, 83% yield, yellow oil. **^{1}H NMR (400 MHz, CDCl$_3$)** δ = 5.68 (t, *J*= 7.3, 7.3 Hz, 1H), 4.57 (t, *J*= 7.4, 7.5 Hz, 1H), 4.41-4.33 (m, 4H), 4.01 (t, *J*= 8.2, 7.9 Hz, 2H), 3.82-3.65 (m, 2H), 2.35-2.19 (m, 1H), 2.05-1.96 (m, 2H), 1.71-1.60 (m, 1H), 1.43-1.29 (m, 8H), 0.94-0.83 (m, 6H) ppm. **^{13}C NMR (100 MHz, CDCl$_3$)** δ = 172.8, 157.6, 153.6, 133.6, 131.5, 62.4, 62.1, 46.9, 46.1, 43.2, 30.7, 30.5, 29.7, 27.3, 22.5, 22.2, 13.9, 13.7 ppm. **HRMS (ESI)** [M+Na$^+$] calculated for $C_{18}H_{28}O_5N_2$ *m/z*=375.1896, found *m/z*=375.1878. **ATR-FTIR (cm^{-1})**: 3535, 2956, 2927, 2860, 1747, 1694, 1479, 1409, 1384, 1362, 1196, 1096, 1037, 970, 929, 830, 758, 731, 700, 619.

3,3'-(2-hexyl-1-oxodec-3-ene-1,3-diyl)bis(oxazolidin-2-one) (5j)

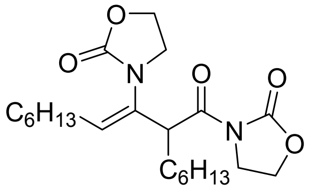

86 mg, 71% yield, yellow oil. **^{1}H NMR (400 MHz, CDCl$_3$)** δ = 5.68 (t, *J*= 7.2, 7.2 Hz, 1H), 4.57 (t, *J*= 7.5, 7.3 Hz, 1H), 4.41-4.34 (m, 4H), 4.00 (t, *J*= 8.0, 7.8 Hz, 2H), 3.82-3.67 (m, 2H), 2.34-2.17 (m, 1H), 2.05-1.97 (m, 2H), 1.72-1.62 (m, 1H), 1.93-1.21 (m, 14H), 0.93-0.83 (m, 8H) ppm. **^{13}C NMR (100 MHz, CDCl$_3$)** δ = 172.8, 156.6, 153.6, 133.7, 131.4, 62.3, 62.1, 46.9, 46.1, 43.3, 31.7, 31.5, 30.8, 29.1, 28.9, 28.6, 27.5, 27.4, 22.5(2C), 14.0(2C) ppm. **HRMS (ESI)** [M+Na$^+$] calculated for $C_{22}H_{36}O_5N_2$ *m/z*=431.2522, found *m/z*=431.2521. **ATR-FTIR (cm^{-1})**: 2954, 2923, 2855, 1749, 1695, 1479, 1408, 1384, 1362, 1216, 1100, 1038, 966, 895, 758, 725, 700.

3,3'-(2,4-dicyclopentyl-1-oxobut-3-ene-1,3-diyl)bis(oxazolidin-2-one) (5k)

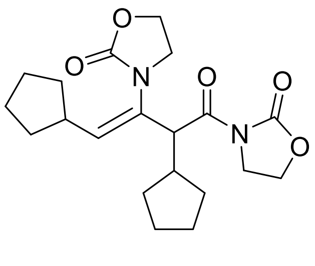

91 mg, 80% yield, yellowish oil. **^{1}H NMR (400 MHz, CDCl$_3$)** δ = 5.68 (t, *J*= 7.2, 7.2 Hz, 1H), 4.57 (t, *J*= 7.5, 7.3 Hz, 1H), 4.41-4.34 (m, 4H), 4.00 (t, *J*= 8.0, 7.8 Hz, 2H), 3.82-3.67 (m, 2H), 2.34-2.17 (m, 1H), 2.05-1.97 (m,

2H), 1.72-1.62 (m, 1H), 1.93-1.21 (m, 14H), 0.93-0.83 (m, 8H) ppm. **^{13}C NMR (100 MHz, CDCl$_3$)** δ = 172.8, 156.6, 153.6, 133.7, 131.4, 62.3, 62.1, 46.9, 46.1, 43.3, 31.7, 31.5, 30.8, 29.1, 28.9, 28.6, 27.5, 27.4, 22.5(2C), 14.0(2C) ppm. **HRMS (ESI)** [M+Na$^+$] calculated for $C_{22}H_{36}O_5N_2$ *m/z*=431.2522, found *m/z*=431.2521. **ATR-FTIR (cm^{-1})**: 2950, 2866, 1770, 1746, 1693, 1410, 1383, 1216, 1103, 1037, 731, 703, 645.

3,3'-(7-chloro-2-(3-chloropropyl)-1-oxohept-3-ene-1,3-diyl)bis(oxazolidin-2-one) (5l)

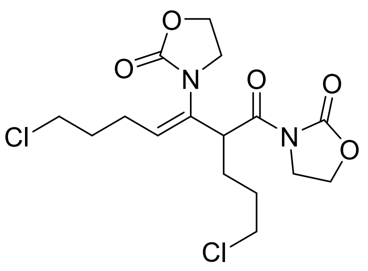

76 mg, 64% yield, yellowish oil. **^{1}H NMR (400 MHz, CDCl$_3$)** δ = 5.69 (t, *J*= 7.3, 7.2 Hz, 1H), 4.61 (t, *J*= 7.2, 7.4 Hz, 1H), 4.44-4.38 (m, 4H), 4.05-3.98 (m, 2H), 3.87-3.74 (m, 2H), 3.61-3.50 (m, 4H), 2.25-2.18 (m, 2H), 2.13-2.02 (m, 1H), 1.93-1.77 (m, 5H). **^{13}C NMR (100 MHz, CDCl$_3$)** δ = 172.1, 156.6, 153.6, 132.5, 132.0, 62.6, 62.1, 46.6, 46.2, 44.5(2C), 43.3, 31.5, 30.8, 28.2, 24.8 ppm. **HRMS (ESI)** [M+Na$^+$] calculated for $C_{16}H_{22}O_5N_2Cl_2$ *m/z*=415.0803, found *m/z*=415.0803. **ATR-FTIR (cm^{-1})**: 3363, 2958, 2919, 1771, 1747, 1693, 1479, 1409, 1385, 1363, 1219, 1110, 1037, 958, 821, 757, 732, 700, 646.

6- Procedure for the Synthesis of 1-Phenethyl-7-phenyltetrahydro-1H,5H-imidazo[1,2-c]oxazol-5-one (7a)

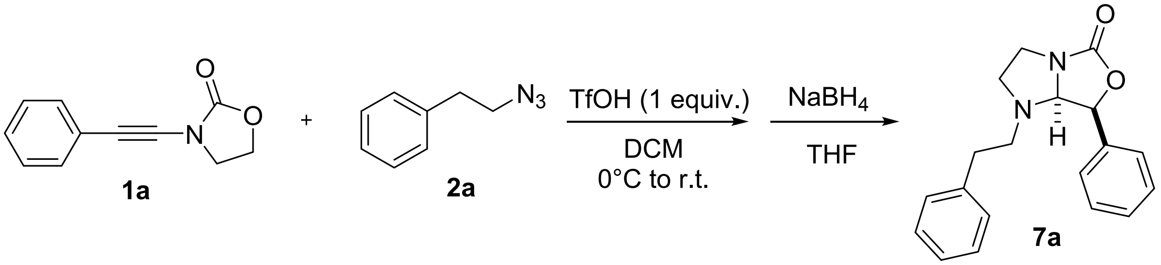

A flame dried Schlenk was charged with the ynamide **1a** (2.0 equiv., 0.6 mmol, 112 mg). The flask was evacuated and backfilled with Ar, then 0.5 ml of DCM were added and the solution was cooled to 0°C. The azide **2a** (1 equiv., 0.3 mmol, 44 mg) was added as a solution in 1 ml of DCM, then TfOH (1 equiv., 0.3 mmol, 27 μl) was added and the mixture was slowly warmed to r.t. After stirring for 13 h, the reduction with sodium borohydrate was performed adding the sodium borohydride (4.0 equiv., 1.2 mmol, 45 mg) in 3 portions at 0 °C over 20 minutes. To increase the solubility of sodium borohydride, 2 ml of dry MeOH were added. The reaction was then warmed to room temperature and stirred for 24 h. Then reaction was quenched with NH_4Cl sat. and washed with brine. Purification by column chromatography using DCM to DCM/DMA (5:1) afforded compound **7a** as white solid in 54% yield.

1-Phenethyl-7-phenyltetrahydro-1H,5H-imidazo[1,2-c]oxazol-5-one (7a)

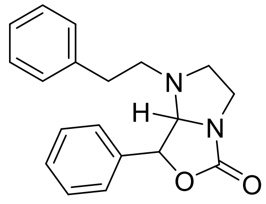

50 mg, 54% yield, white solid. **^{1}H NMR (400 MHz, $CDCl_3$)** δ = 7.43-7.41 (m, 5H), 7.18-7.12 (m, 3H), 6.71 (d, *J*= 6.6 Hz, 2H), 5.54 (d, *J*= 5.4 Hz, 1H), 4.11 (d, *J*= 5.4 Hz, 1H), 3.88 (dt, *J*= 10.8, 8.1 Hz, 1H), 3.48 (td, *J*= 8.7, 3.6 Hz, 1H), 3.39-3.33 (m, 1H), 2.43-2.36 (m, 1H), 2.22-2.15 (m, 1H), 1.93-1.86 (m, 1H), 1.79 (td, *J*= 11.5, 5.6 Hz, 1H). **^{13}C NMR (100 MHz, $CDCl_3$)** 162.5, 139.3, 133.0, 129.3, 128.6, 128.4, 128.3, 127.9, 126.2, 81.4, 55.1, 52.6, 45.1, 35.2. **HRMS (ESI)** [$M+H^+$] calculated for $C_{19}H_{20}O_2N_2$ *m/z*= 309.1598, found *m/z*= 309.1598. **ATR-FTIR (cm^{-1})**: 3064, 2911, 2827, 1762, 1603, 1496, 1456, 1383, 1246, 1198, 1167, 1046, 753, 699, 626.

7- Procedure for the synthesis of fully-substituted pyridine:

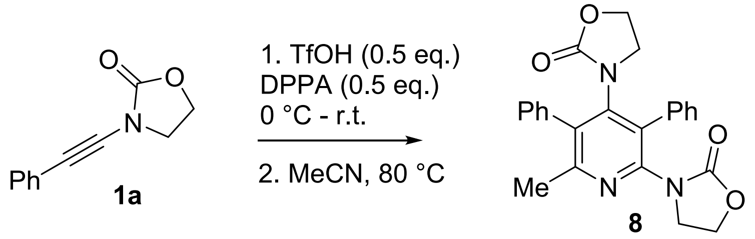

A flame-dried Schlenk tube was charged with ynamide **1a** (2 equiv., 0.4 mmol, 75 mg), evacuated and backfilled with Argon. Dry DCM (0.3 mL) was added and the solution was cooled to 0 °C. A solution of dppa **4** (1.0 equiv., 0.2 mmol, 55 mg) in dry DCM (0.7 mL) was added followed by addition of triflic acid (1.0 equiv., 0.2 mmol, 18 µL), and the mixture was stirred for 16 h at room temperature. The tube was equipped with a septa and dry acetonitrile (1 mL) was added. The septum was punctured with a needle, the mixture was heated to 65 °C and the DCM was removed from the mixture by means of an Argon stream (for 1 h). The tube was closed with a stopper and stirred for 20 h at 80 °C. After removal of the solvent under reduced pressure, the product was purified by preparative HPLC (column Waters, X select CSH perp C18, 5 µm, 30x150mm, Acetonitrile/ 1mM NH_4HCO_3 solution in water from 10% to 95%, flow 20 mL/min).

3,3'-(6-Methyl-3,5-diphenylpyridine-2,4-diyl)bis(oxazolidin-2-one) (8a)

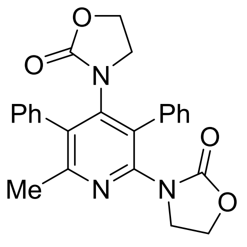

16.5 mg, 20% yield, yellow oil. **^{1}H NMR (400 MHz, $CDCl_3$):** 6.97-6.89 (m, 8H), 6.81-6.79 (m, 2H), 4.29-4.21 (m, 6H), 3.59-3.55 (m, 2H), 1.72 (s, 3H). **^{13}C NMR (100 MHz, $CDCl_3$):** 170.3, 156.8, 149.1, 140.6, 136.2, 134.7, 132.6, 130.7, 129.0,

128.3, 127.8, 127.7, 127.3, 126.7, 117.8, 63.1, 58.2, 48.7, 44.6, 27.1. **ATR-FTIR (cm^{-1})**: 3286, 1771, 1715, 1657, 1619, 1543, 1495, 1420, 1367, 1342, 1234, 1026, 757, 703. **HRMS (ESI)**: $[M + H]^+$ calculated for $C_{24}H_{22}N_3O_4^+$ *m/z* = 416.1605, found *m/z* = 416.1603.

8- Procedure for the derivatization of oxazolidine-dione 3a

A flame-dried Schlenk tube was charged with oxazolidine-dione **3a** (1 equiv., 1.0 mmol, 347 mg), evacuated and backfilled with Argon. Dry ether (30 mL) was added and the solution was cooled to 0 °C. A mixture of Et_3N (2.0 equiv., 2.0 mmol, 0.3ml) and acetyl chloride (2.0 equiv., 2.0 mmol, 0.15ml) were added and the mixture was stirred for 12 h at room temperature. The mixture was then quenched with NH_4Cl and extracted with DCM (2x15ml). After removal of the solvent under reduced pressure, the product was used directly for the next step.

A solution of acylated oxazolidine-dione (1equiv., 0.1mmol, 37mg) dissolved in 1ml THF was cooled down to -78°C and LHMDS (1.2 equiv., 0.12mmol, 1M in THF, 0.12ml) was added dropwise. The mixture was stirred at -78°C for 30min. Then alkyl halide (2 equiv., 0.2mmol) was added and the mixture was stirred for another 30min at -78°C and another 1h at r.t. The mixture was quenched with NH_4Cl, extracted with ether (2x5ml) and dried over Na_2SO_4. Purification by column chromatography using DCM to DCM/DMA (5:1) afforded compounds **9a-b**.

***N*-(2-(5-methyl-2,4-dioxo-5-phenyloxazolidin-3-yl)ethyl)-N-phenethylacetamide (9a)**

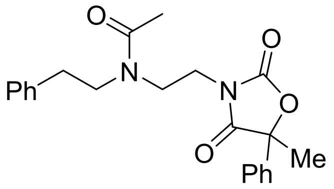

28 mg, 73% yield, yellowish oil. **^{1}H NMR (400 MHz, $CDCl_3$):** 7.49-7.45 (m, 2H), 7.34-7.25 (m, 3H), 7.24-7.19 (m, 2H), 7.17-7.09 (m, 1H), 7.02 (d, *J*= 7.4 Hz, 2H), 3.66-3.59 (m, 2H), 3.43-3.28 (m, 4H), 2,70 (t, *J*= 7.3, 7.3 Hz, 2H), 1.82 (s, 3H), 1.71 (s, 3H). **^{13}C NMR (100 MHz, $CDCl_3$):** 174.4, 171.5, 154.4, 138.0, 136.8, 128.8(3C), 128.7(3C), 126.8, 124.4(3C), 85.8, 50.3, 42.6, 38.4, 35.2, 24.9, 20.9. **ATR-FTIR (cm^{-1})**: 3181, 1851, 1780, 1714, 1633, 1613, 1505, 1495, 1367, 1318, 1236, 1020, 744, 703. **HRMS (ESI)**: $[M + H]^+$ calculated for $C_{22}H_{24}N_2O_4Na$ *m/z* = 403.1634, found *m/z* = 403.1634.

***N*-(2-(5-allyl-2,4-dioxo-5-phenyloxazolidin-3-yl)ethyl)-N-phenethylacetamide (9b)**

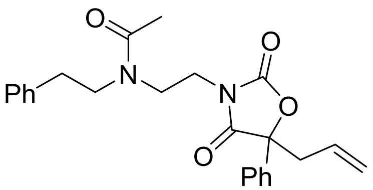

28 mg, 69% yield, yellowish oil. **^{1}H NMR (400 MHz, $CDCl_3$):** 7.61-7.56 (m, 2H), 7.45-7.33 (m, 3H), 7.33-7.28 (m, 2H), 7.25-7.19 (m, 1H), 7.08 (d, *J*= 8.1 Hz, 2H), 5.70-5.58 (m, 1H), 5.25-5.13 (m, 2H), 3.82-

3.61 (m, 2H), 3.44-3.28 (m, 2H), 3.01-2.85 (m, 2H), 2,75 (t, *J*= 7.7, 7.4 Hz, 2H), 1.74 (s, 3H). **^{13}C NMR (100 MHz, $CDCl_3$):** 173.3, 171.1, 154.5, 137.8, 135.4, 129.0, 128.9, 128.7, 128.6, 128.5(2C), 128.3, 126.8, 124.7(2C), 121.7, 87.7, 50.6, 42.9, 42.5, 38.2, 35.5, 21.4, 20.8. **ATR-FTIR (cm^{-1})**: 2927, 1814, 1736, 1684, 1645, 1435, 1409, 1197, 1089, 1033, 732, 699. **HRMS (ESI)**: $[M + H]^+$ calculated for $C_{24}H_{26}N_2O_4Na$ *m/z* = 429.1790, found *m/z* = 429.1778.

9- Spectra:

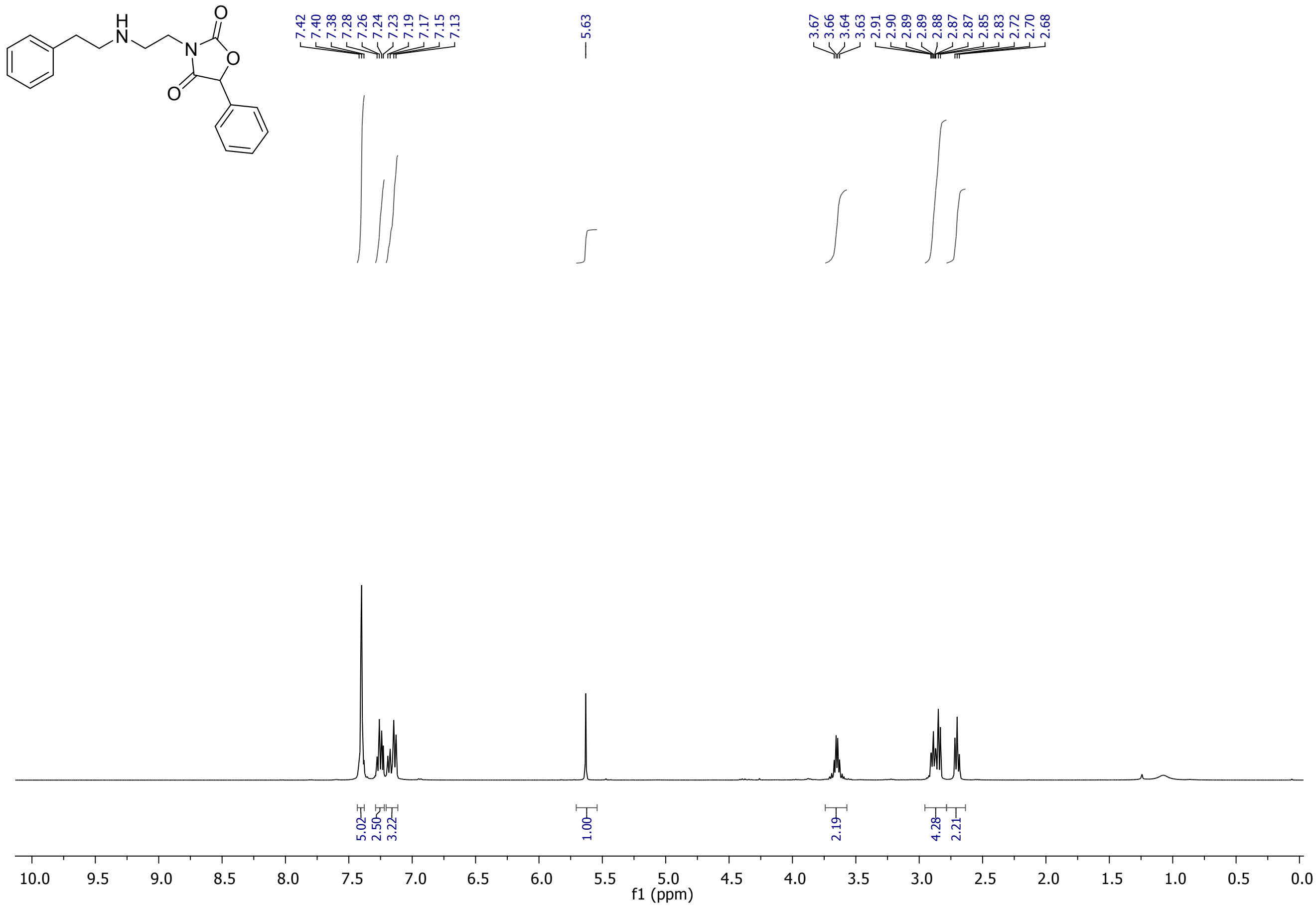
7.42
7.40
7.38
7.28
7.26
7.24
7.23
7.19
7.17
7.15
7.13
5.63
3.67
3.66
3.64
3.63
2.91
2.90
2.89
2.89
2.88
2.87
2.87
2.85
2.83
2.72
2.70
2.68
5.02
2.50
3.22
1.00
2.19
4.28
2.21
10.0 9.5 9.0 8.5 8.0 7.5 7.0 6.5 6.0 5.5 5.0 4.5 4.0 3.5 3.0 2.5 2.0 1.5 1.0 0.5 0.0
f1 (ppm)

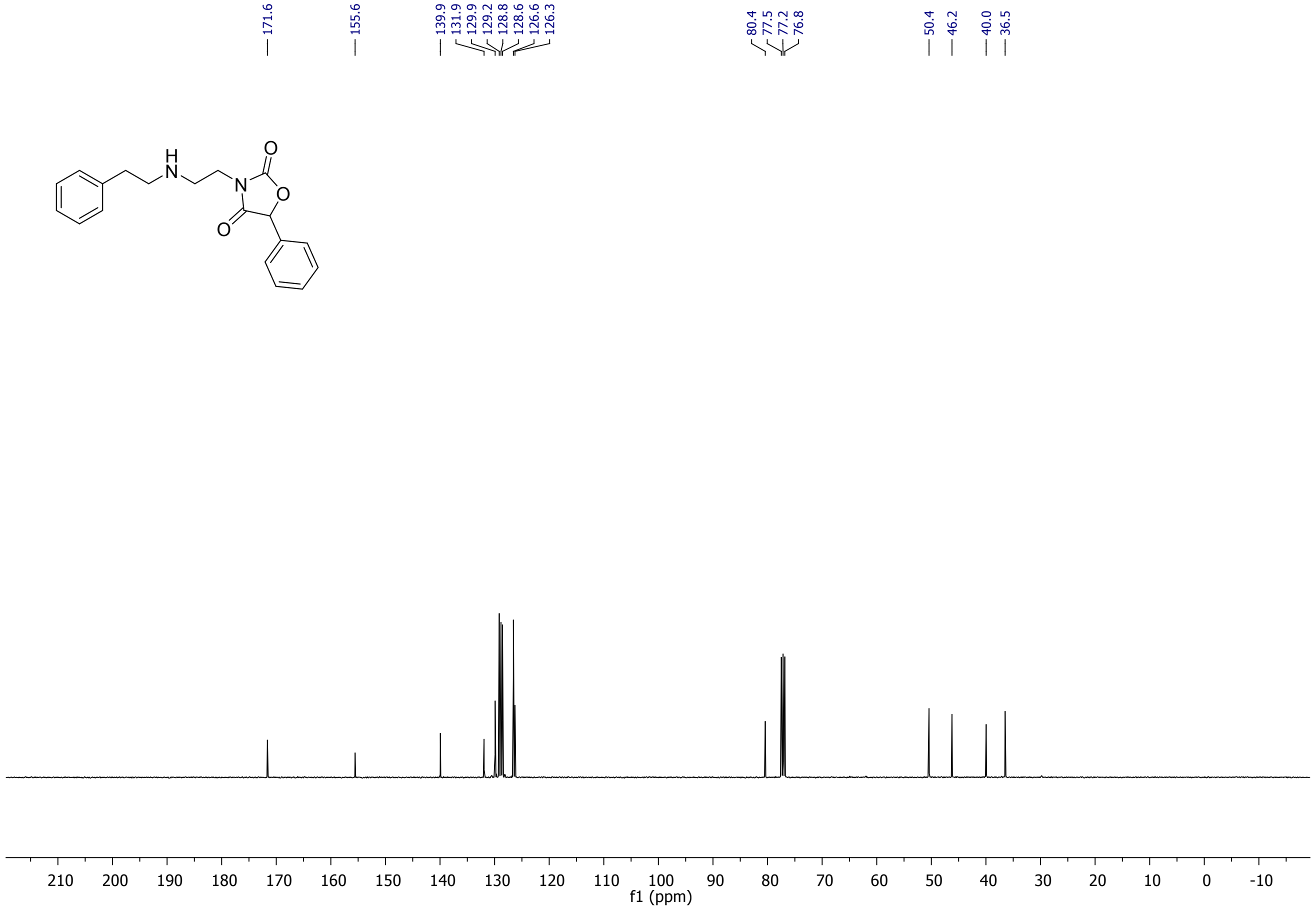
171.6
155.6
139.9
131.9
129.9
129.2
128.8
128.6
126.6
126.3
80.4
77.5
77.2
76.8
50.4
46.2
40.0
36.5
210 200 190 180 170 160 150 140 130 120 110 100 90 80 70 60 50 40 30 20 10 0 -10
f1 (ppm)

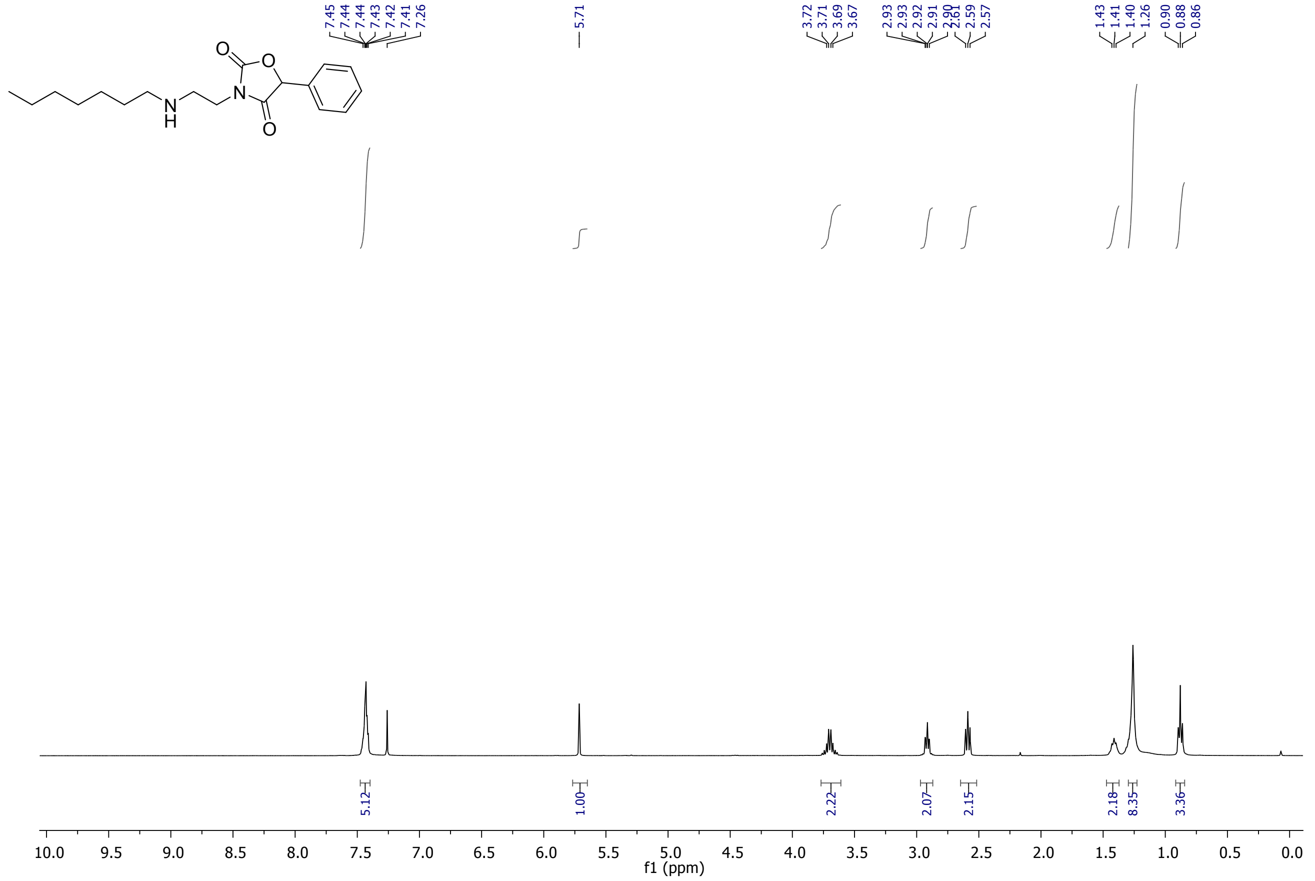
7.45
7.44
7.44
7.43
7.42
7.41
7.26
5.71
3.72
3.71
3.69
3.67
2.93
2.93
2.92
2.91
2.90
2.61
2.59
2.57
1.43
1.41
1.40
1.26
0.90
0.88
0.86
O
N
H
5.12
1.00
2.22
2.07
2.15
2.18
8.35
3.36
f1 (ppm)

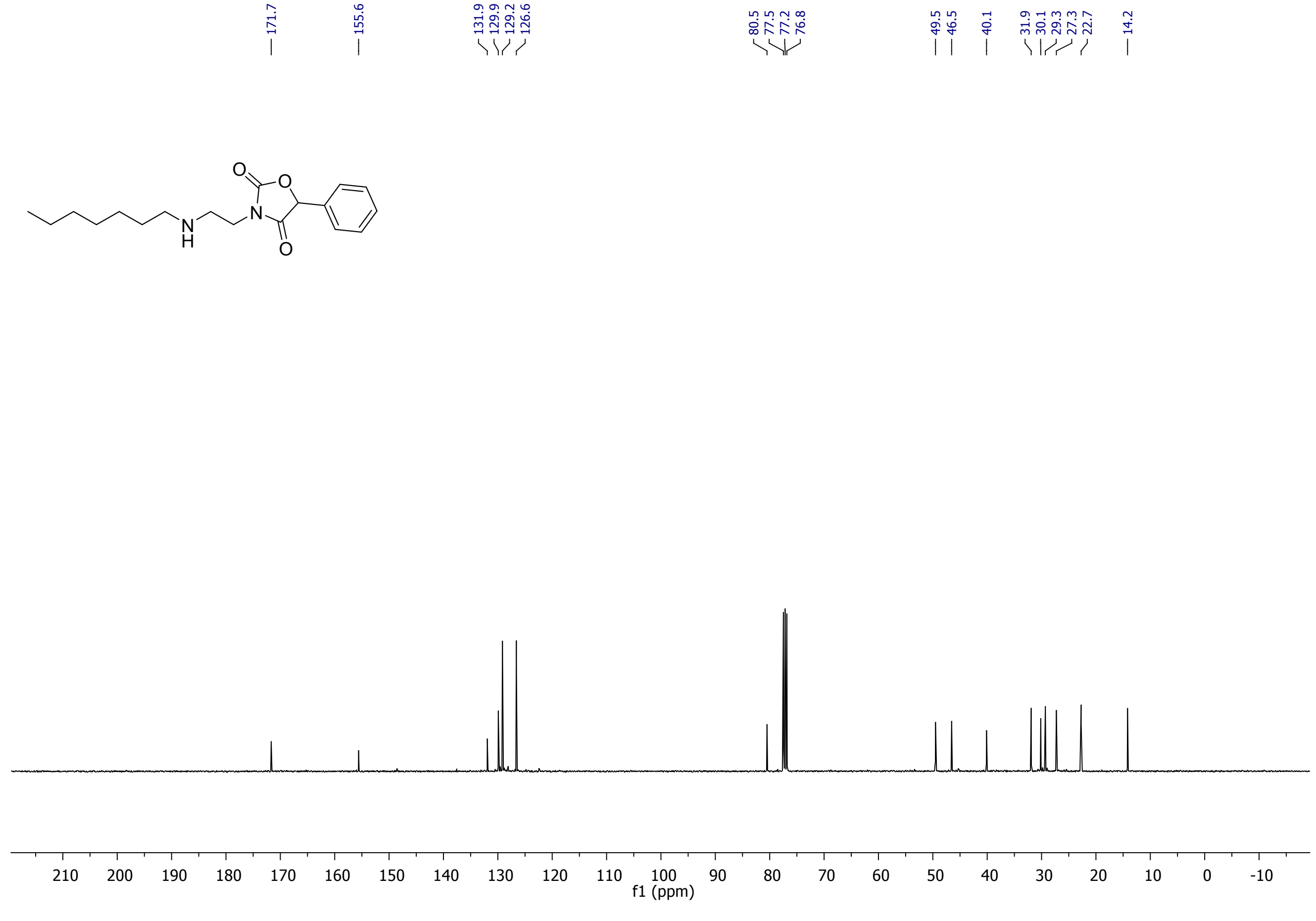
171.7
155.6
131.9
129.9
129.2
126.6
80.5
77.5
77.2
76.8
49.5
46.5
40.1
31.9
30.1
29.3
27.3
22.7
14.2
O
N
H
f1 (ppm)

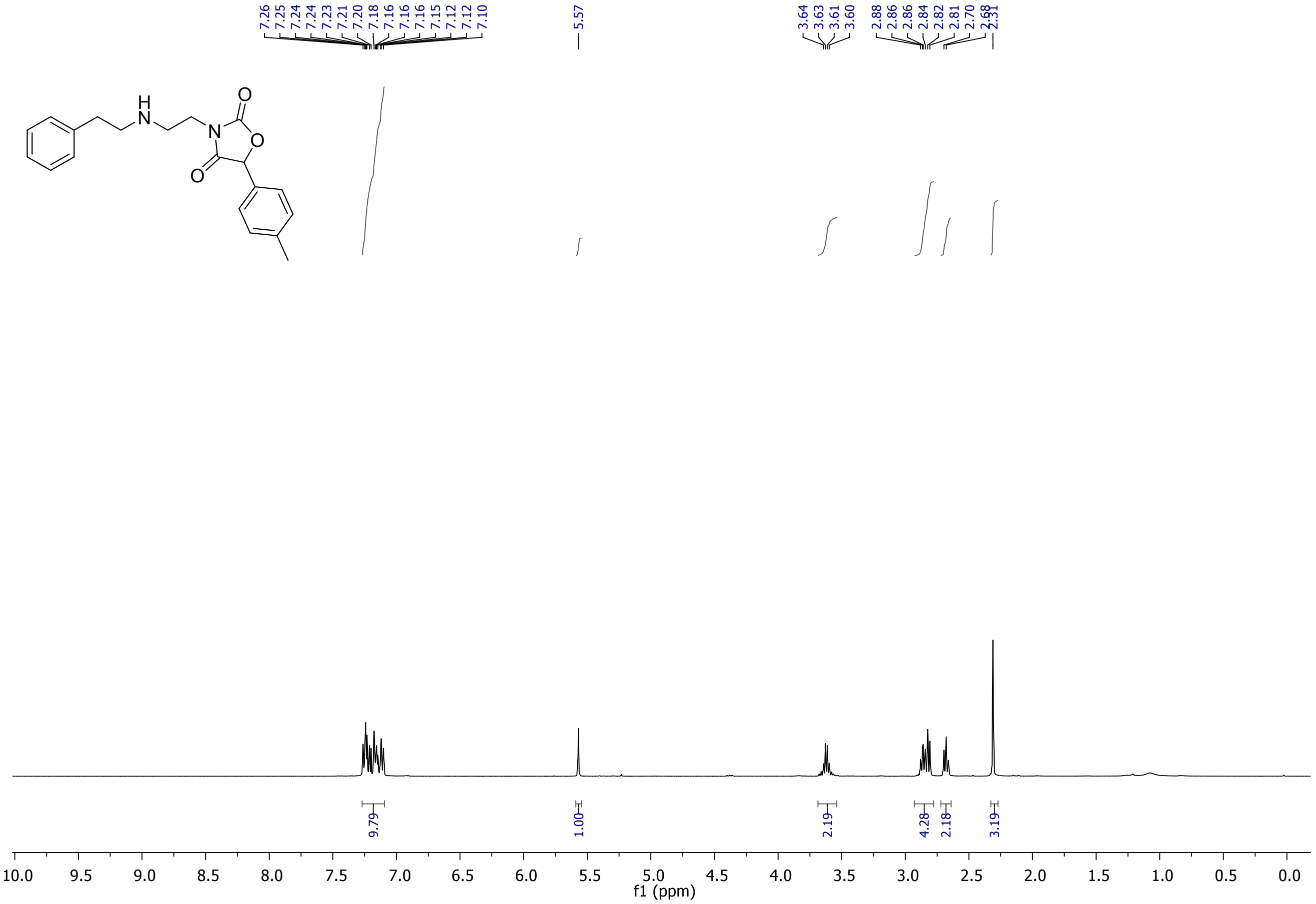
7.26 7.25 7.24 7.24 7.23 7.21 7.20 7.18 7.16 7.16 7.16 7.15 7.12 7.12 7.10
5.57
3.64 3.63 3.61 3.60
2.88 2.86 2.86 2.84 2.82 2.81 2.70 2.68 2.31
H
N
O
O
N
O
9.79
1.00
2.19
4.28
2.18
3.19
10.0 9.5 9.0 8.5 8.0 7.5 7.0 6.5 6.0 5.5 5.0 4.5 4.0 3.5 3.0 2.5 2.0 1.5 1.0 0.5 0.0
f1 (ppm)

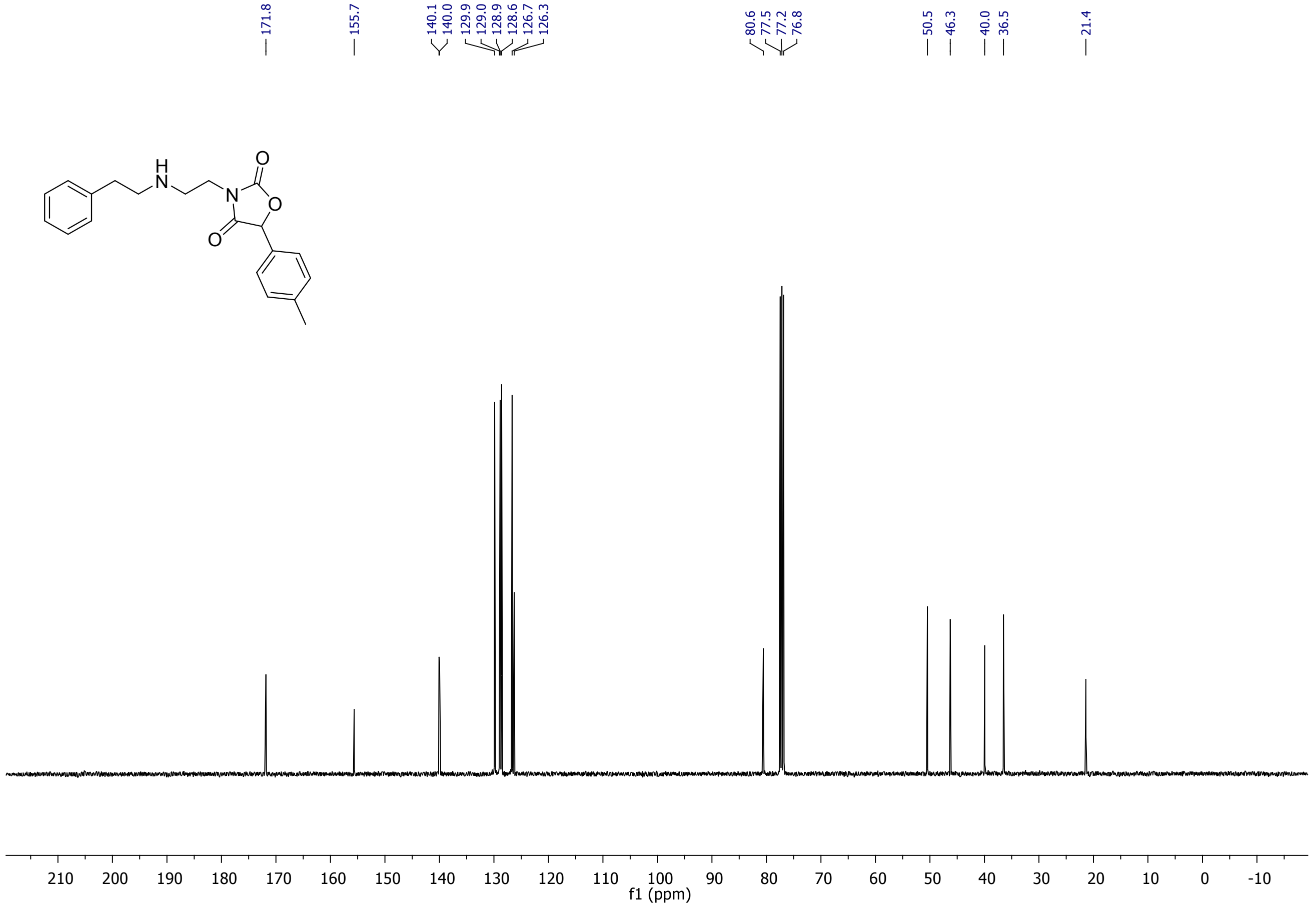
171.8
155.7
140.1 140.0
129.9 129.0 128.9 128.6 126.7 126.3
80.6 77.5 77.2 76.8
50.5
46.3
40.0
36.5
21.4
H
N
O
O
N
O
210 200 190 180 170 160 150 140 130 120 110 100 90 80 70 60 50 40 30 20 10 0 -10
f1 (ppm)

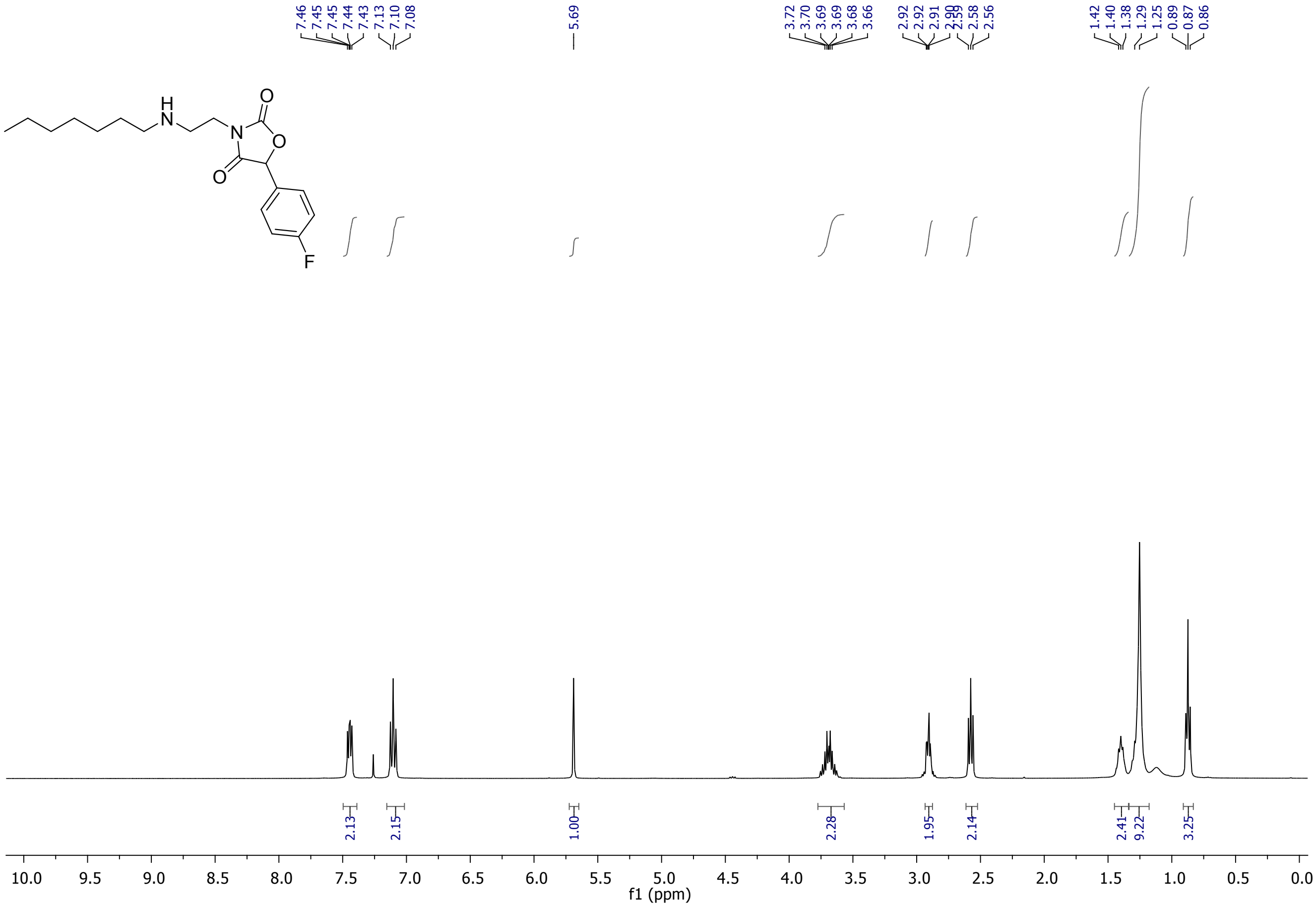
7.46
7.45
7.45
7.44
7.43
7.13
7.10
7.08
5.69
3.72
3.70
3.69
3.69
3.68
3.66
2.92
2.92
2.91
2.90
2.59
2.58
2.56
1.42
1.40
1.38
1.29
1.25
0.89
0.87
0.86
F
2.13
2.15
1.00
2.28
1.95
2.14
2.41
9.22
3.25
f1 (ppm)

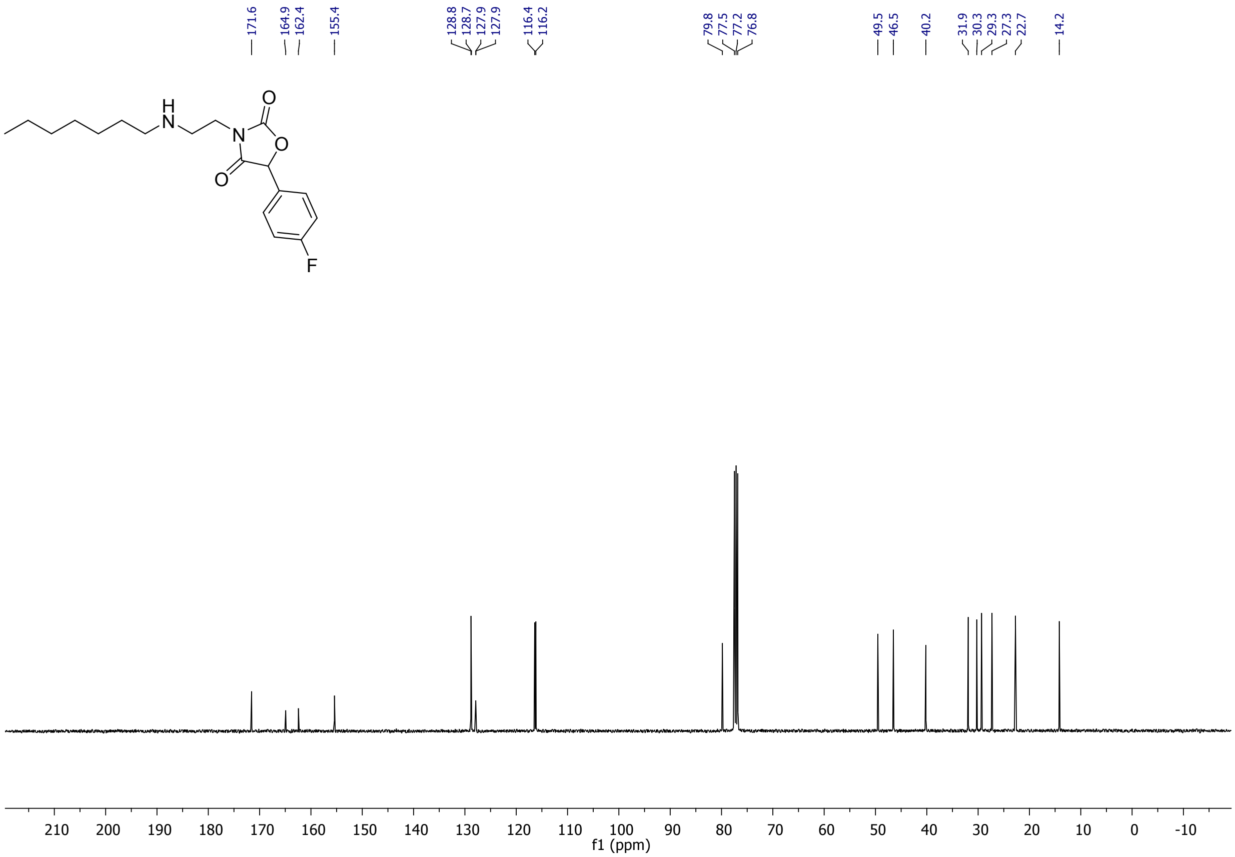
171.6
164.9
162.4
155.4
128.8
128.7
127.9
127.9
116.4
116.2
79.8
77.5
77.2
76.8
49.5
46.5
40.2
31.9
30.3
29.3
27.3
22.7
14.2
F
f1 (ppm)

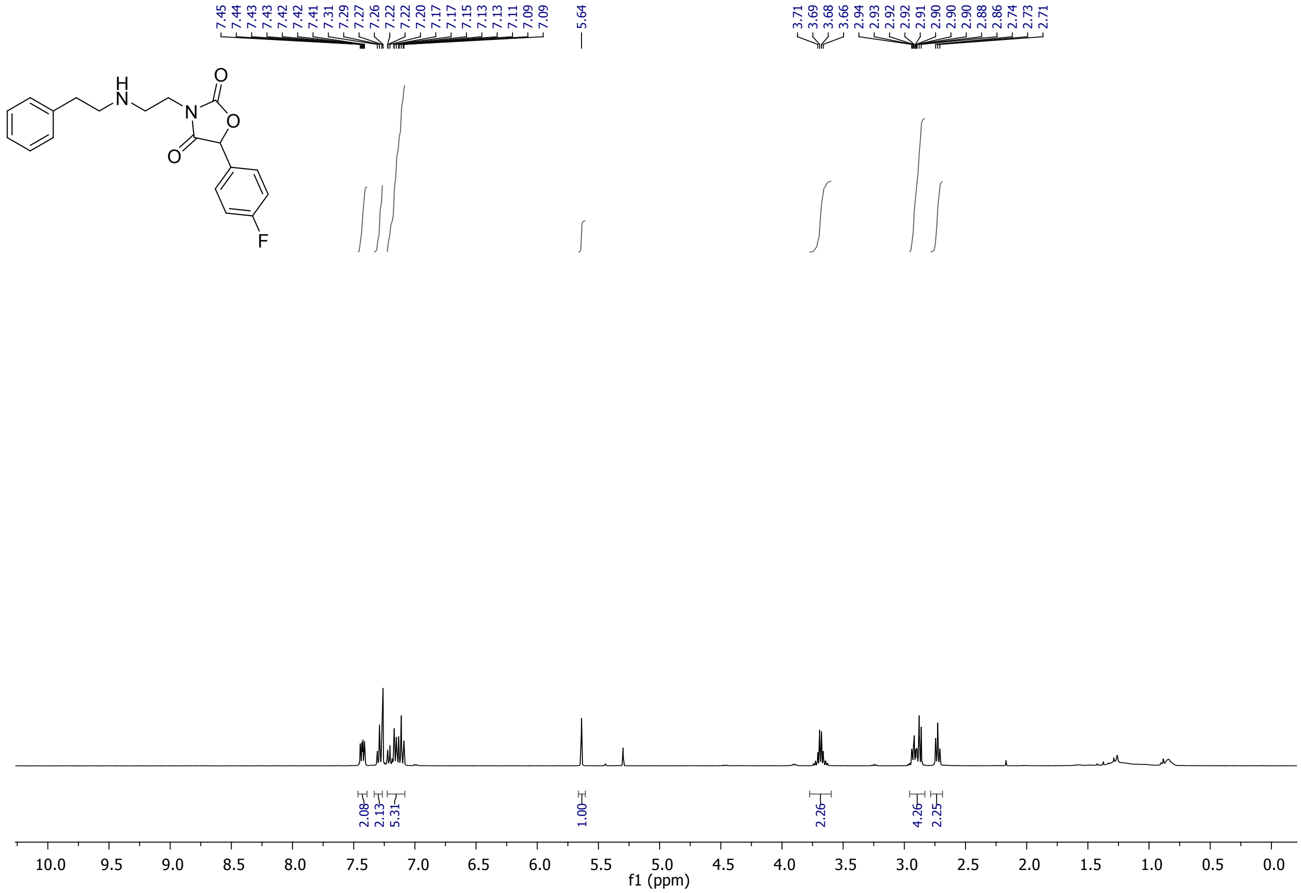

7.45 7.44 7.43 7.43 7.42 7.42 7.41 7.31 7.29 7.27 7.26 7.22 7.22 7.20 7.17 7.17 7.15 7.13 7.11 7.09 7.09
5.64
3.71 3.69 3.68 3.66 2.94 2.93 2.92 2.92 2.91 2.90 2.90 2.90 2.88 2.86 2.74 2.73 2.71
H
N
O
F
2.08 2.13 5.31 1.00 2.26 4.26 2.25
f1 (ppm)

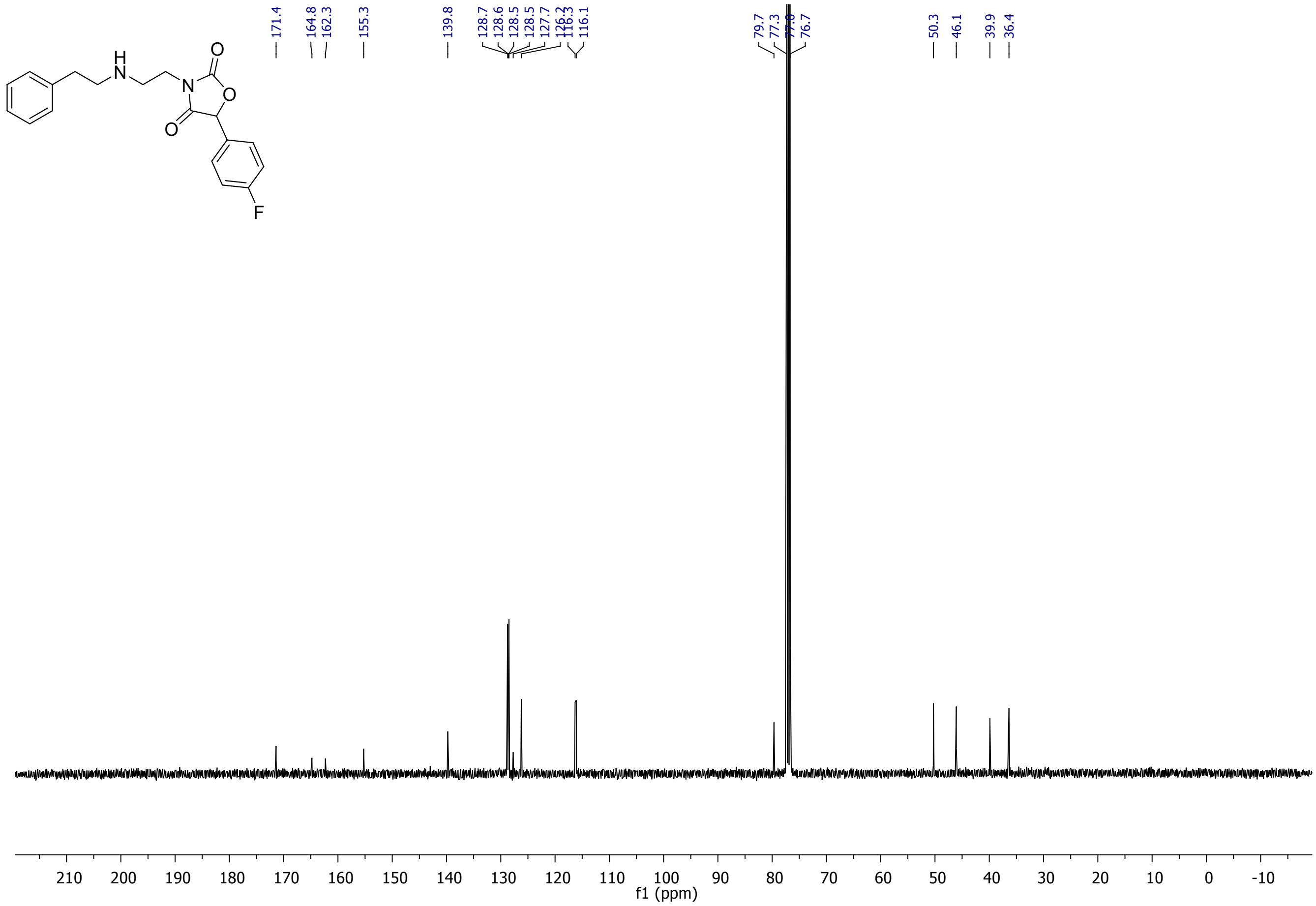

171.4 164.8 162.3 155.3 139.8 128.7 128.6 128.5 128.5 127.7 126.2 116.3 116.1
79.7 77.3 77.0 76.7
50.3 46.1 39.9 36.4
H
N
O
F
f1 (ppm)

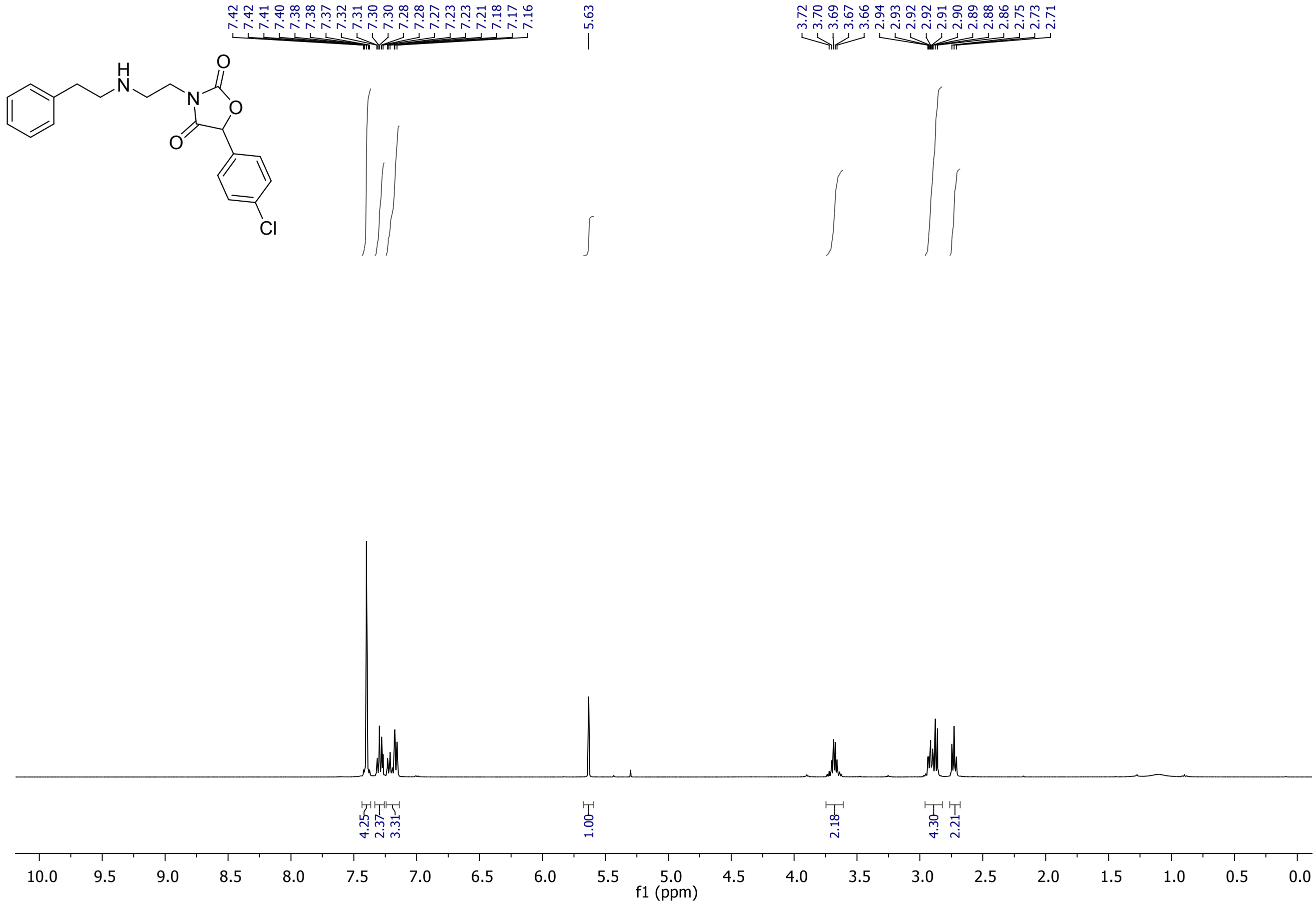
7.42 7.42 7.41 7.40 7.38 7.38 7.37 7.32 7.31 7.30 7.30 7.28 7.28 7.27 7.23 7.23 7.21 7.18 7.17 7.16
5.63
3.72 3.70 3.69 3.67 3.66 2.94 2.93 2.92 2.92 2.91 2.90 2.89 2.88 2.86 2.75 2.73 2.71
H
N
O
O
N
O
Cl
4.25 2.37 3.31
1.00
2.18
4.30 2.21
f1 (ppm)

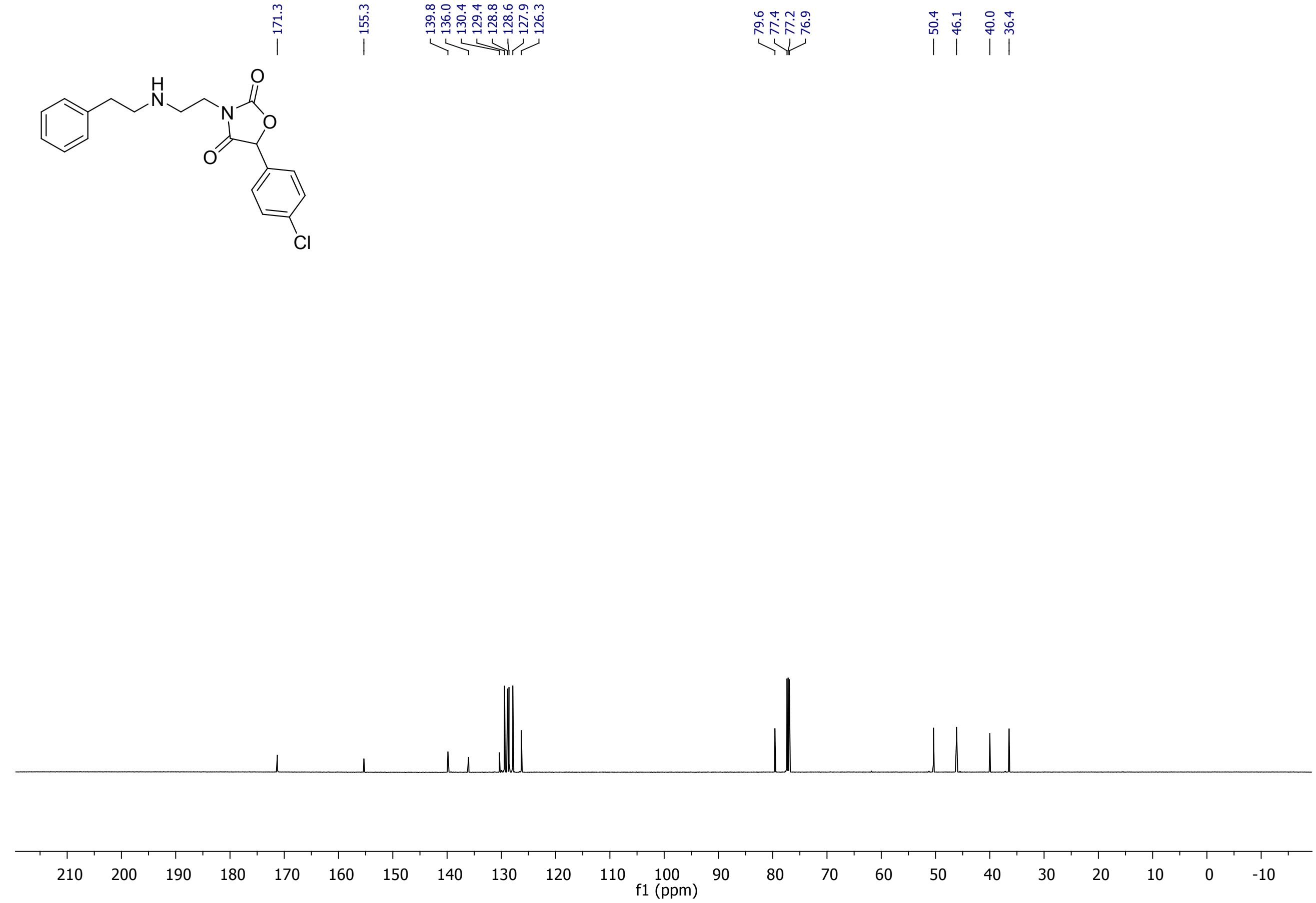
171.3
155.3
139.8 136.0 130.4 129.4 128.8 128.6 127.9 126.3
79.6 77.4 77.2 76.9
50.4 46.1 40.0 36.4
H
N
O
O
N
O
Cl
f1 (ppm)

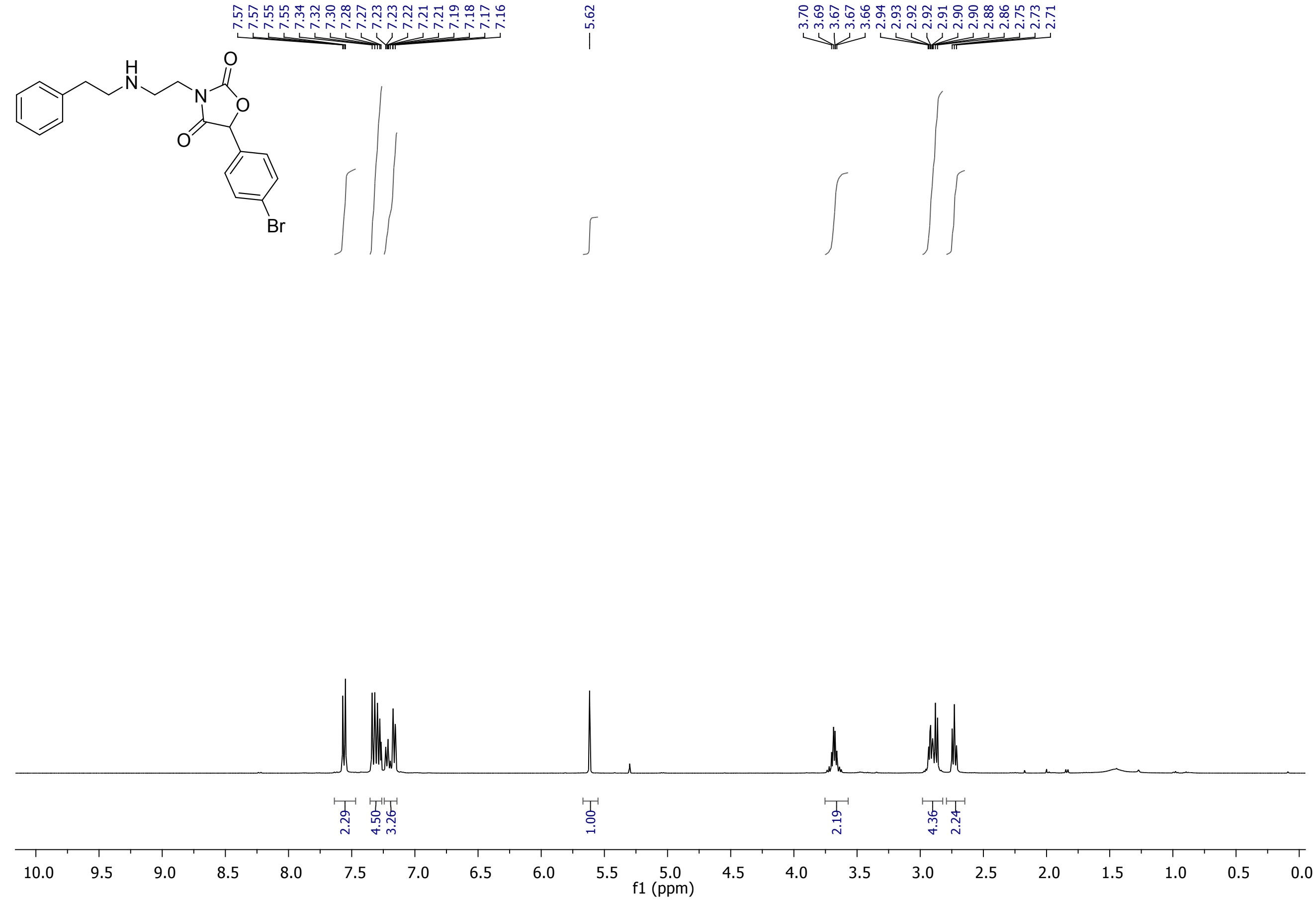

7.57
7.57
7.55
7.34
7.32
7.30
7.28
7.27
7.23
7.23
7.22
7.21
7.21
7.19
7.18
7.17
7.16
5.62
3.70
3.69
3.67
3.67
3.66
2.94
2.93
2.92
2.92
2.91
2.90
2.90
2.88
2.86
2.75
2.73
2.71
H
N
O
O
O
Br
2.29
4.50
3.26
1.00
2.19
4.36
2.24
10.0 9.5 9.0 8.5 8.0 7.5 7.0 6.5 6.0 5.5 5.0 4.5 4.0 3.5 3.0 2.5 2.0 1.5 1.0 0.5 0.0
f1 (ppm)

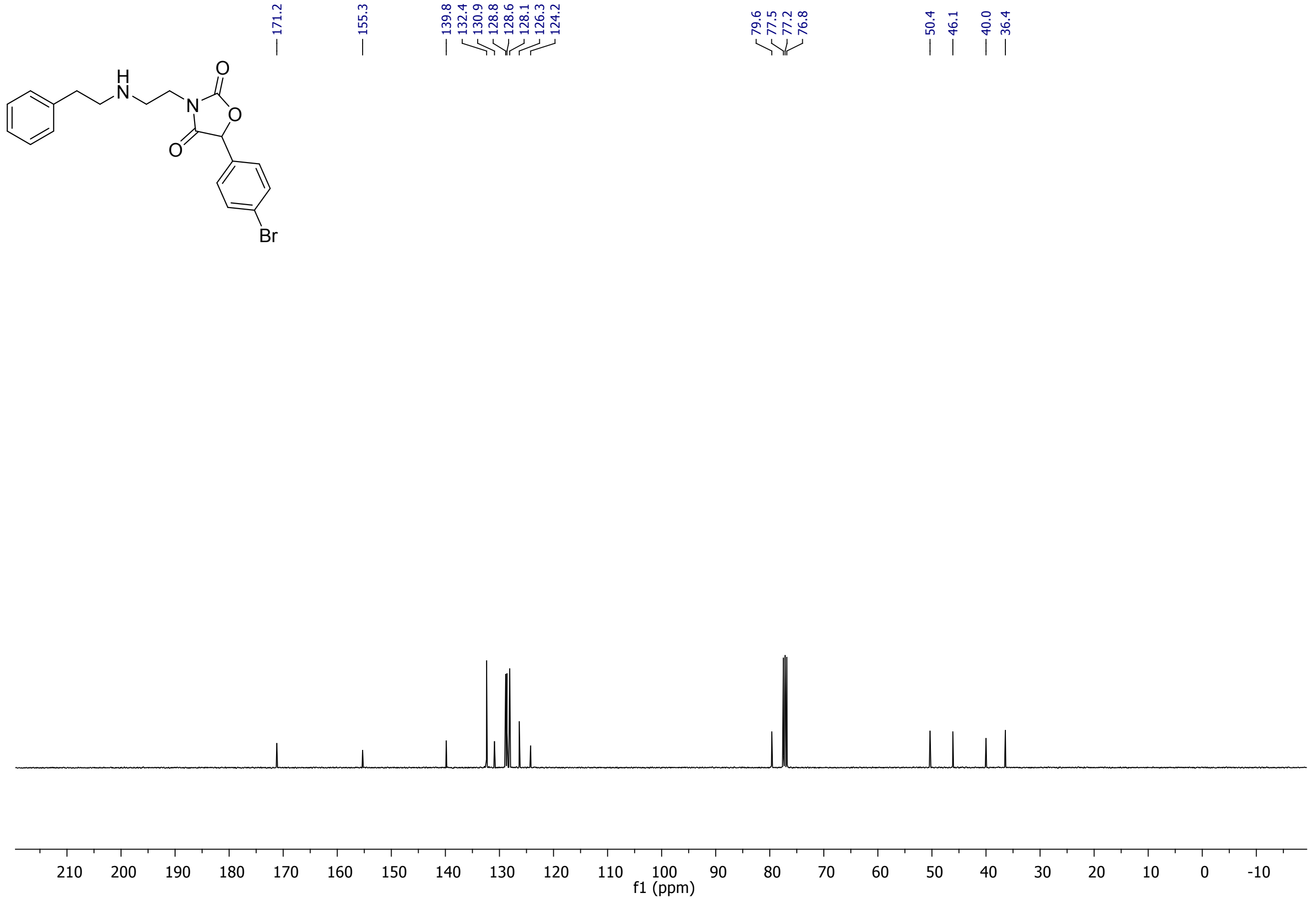

171.2
155.3
139.8
132.4
130.9
128.8
128.6
128.1
126.3
124.2
79.6
77.5
77.2
76.8
50.4
46.1
40.0
36.4
H
N
O
O
O
Br
210 200 190 180 170 160 150 140 130 120 110 100 90 80 70 60 50 40 30 20 10 0 -10
f1 (ppm)

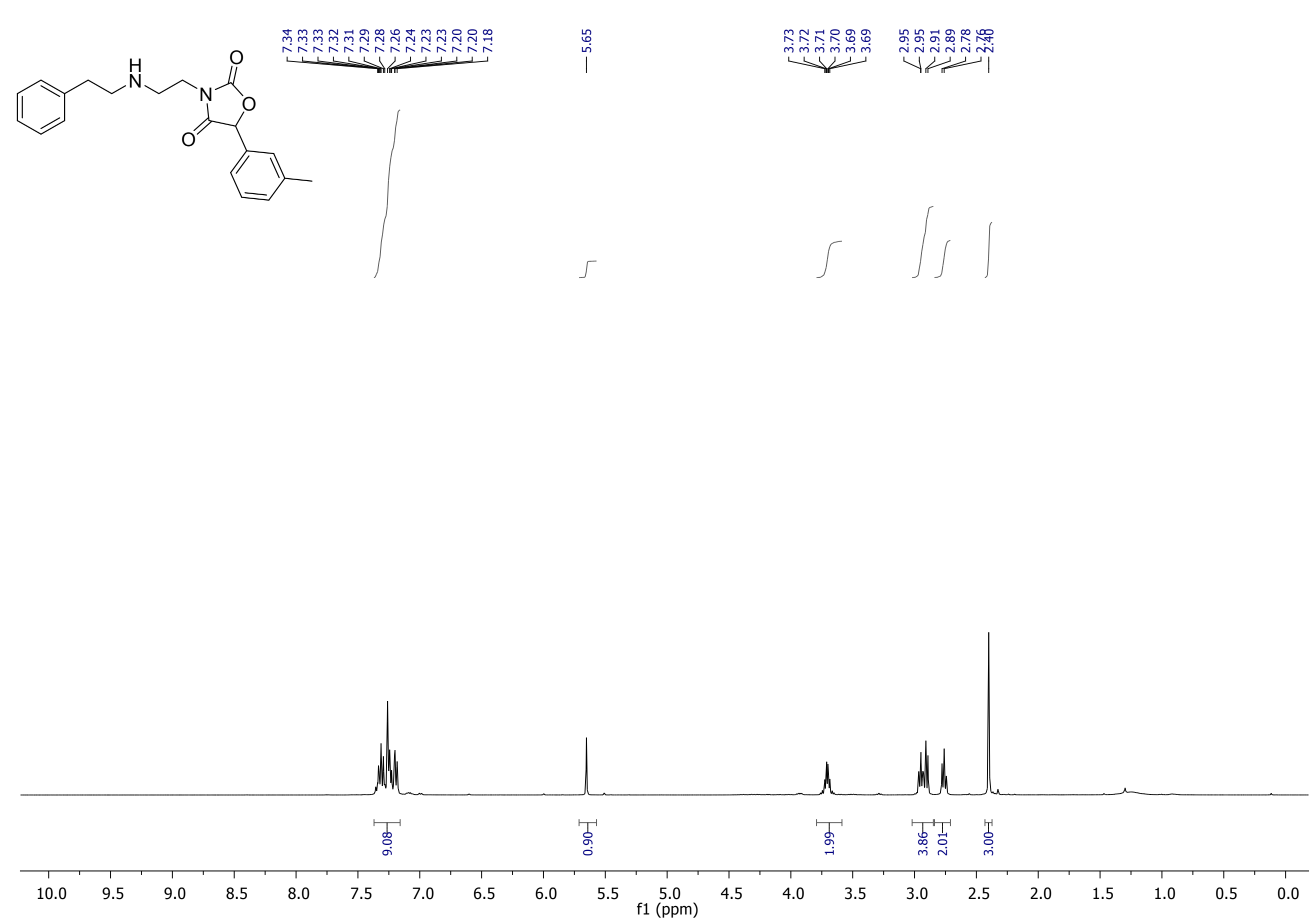
7.34 7.33 7.33 7.32 7.31 7.29 7.28 7.26 7.24 7.23 7.23 7.20 7.20 7.18
5.65
3.73 3.72 3.71 3.70 3.69 3.69
2.95 2.95 2.91 2.89 2.78 2.76 2.40
H
N
O
O
O
9.08
0.90
1.99
3.86
2.01
3.00
10.0 9.5 9.0 8.5 8.0 7.5 7.0 6.5 6.0 5.5 5.0 4.5 4.0 3.5 3.0 2.5 2.0 1.5 1.0 0.5 0.0
f1 (ppm)

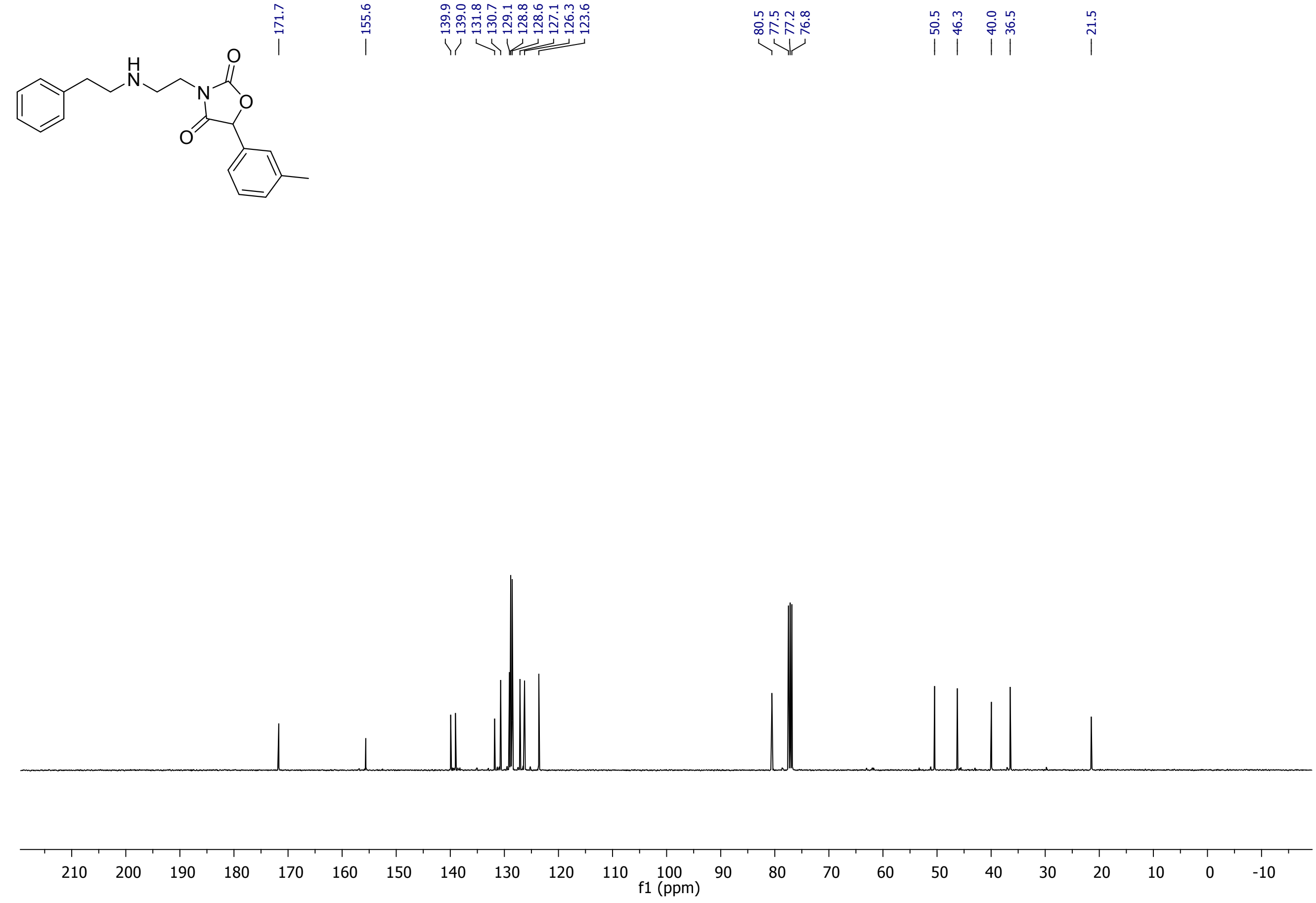
171.7
155.6
139.9 139.0 131.8 130.7 129.1 128.8 128.6 127.1 126.3 123.6
80.5 77.5 77.2 76.8
50.5 46.3 40.0 36.5
21.5
H
N
O
O
O
210 200 190 180 170 160 150 140 130 120 110 100 90 80 70 60 50 40 30 20 10 0 -10
f1 (ppm)

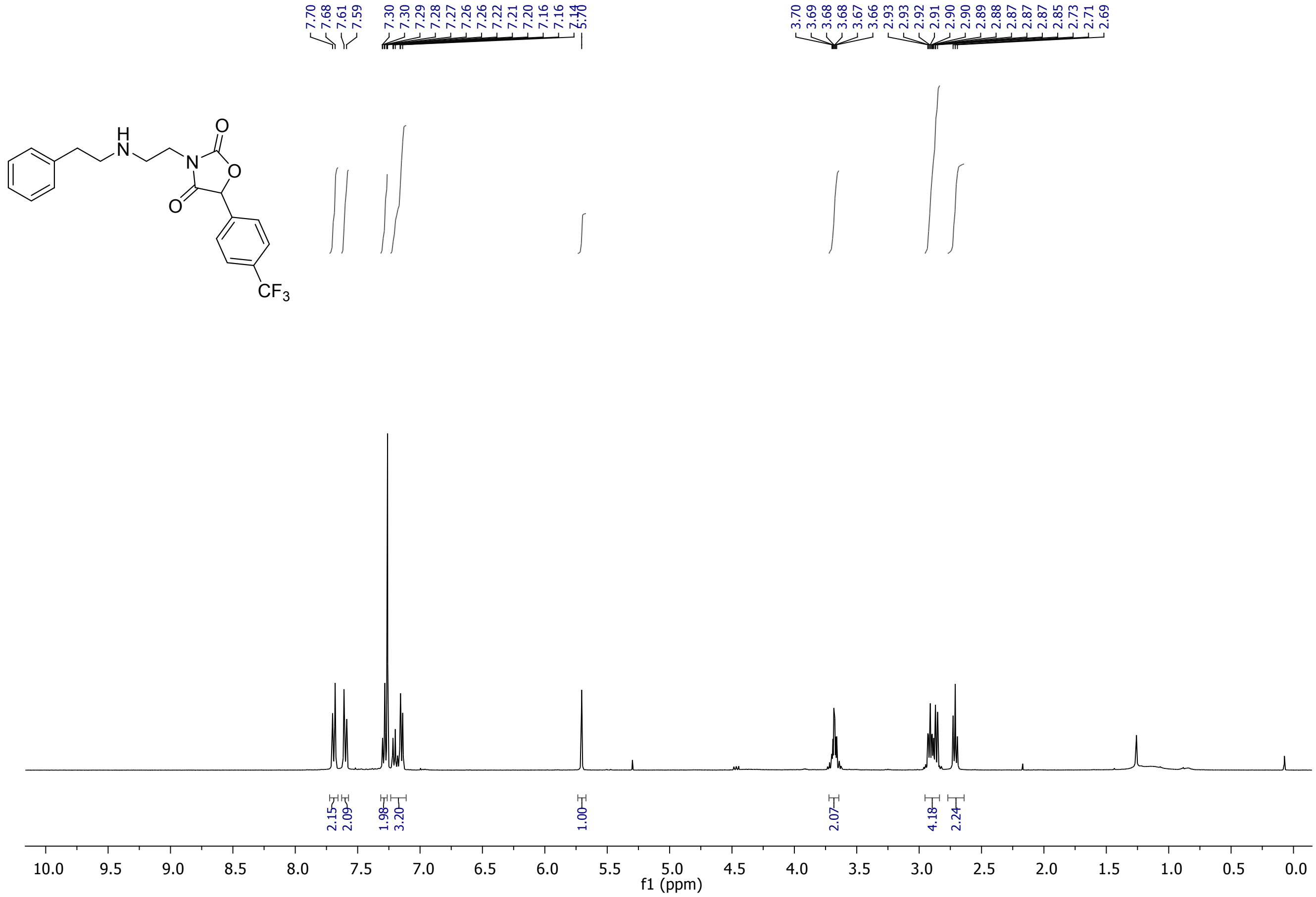
7.70 7.68 7.61 7.59 7.30 7.30 7.29 7.28 7.27 7.26 7.22 7.21 7.20 7.16 7.16 7.14 5.70
3.70 3.69 3.68 3.68 3.67 3.66 2.93 2.93 2.92 2.91 2.90 2.90 2.89 2.88 2.87 2.87 2.87 2.85 2.73 2.71 2.69
H
N
O
CF3
2.15 2.09 1.98 3.20 1.00 2.07 4.18 2.24
10.0 9.5 9.0 8.5 8.0 7.5 7.0 6.5 6.0 5.5 5.0 4.5 4.0 3.5 3.0 2.5 2.0 1.5 1.0 0.5 0.0
f1 (ppm)

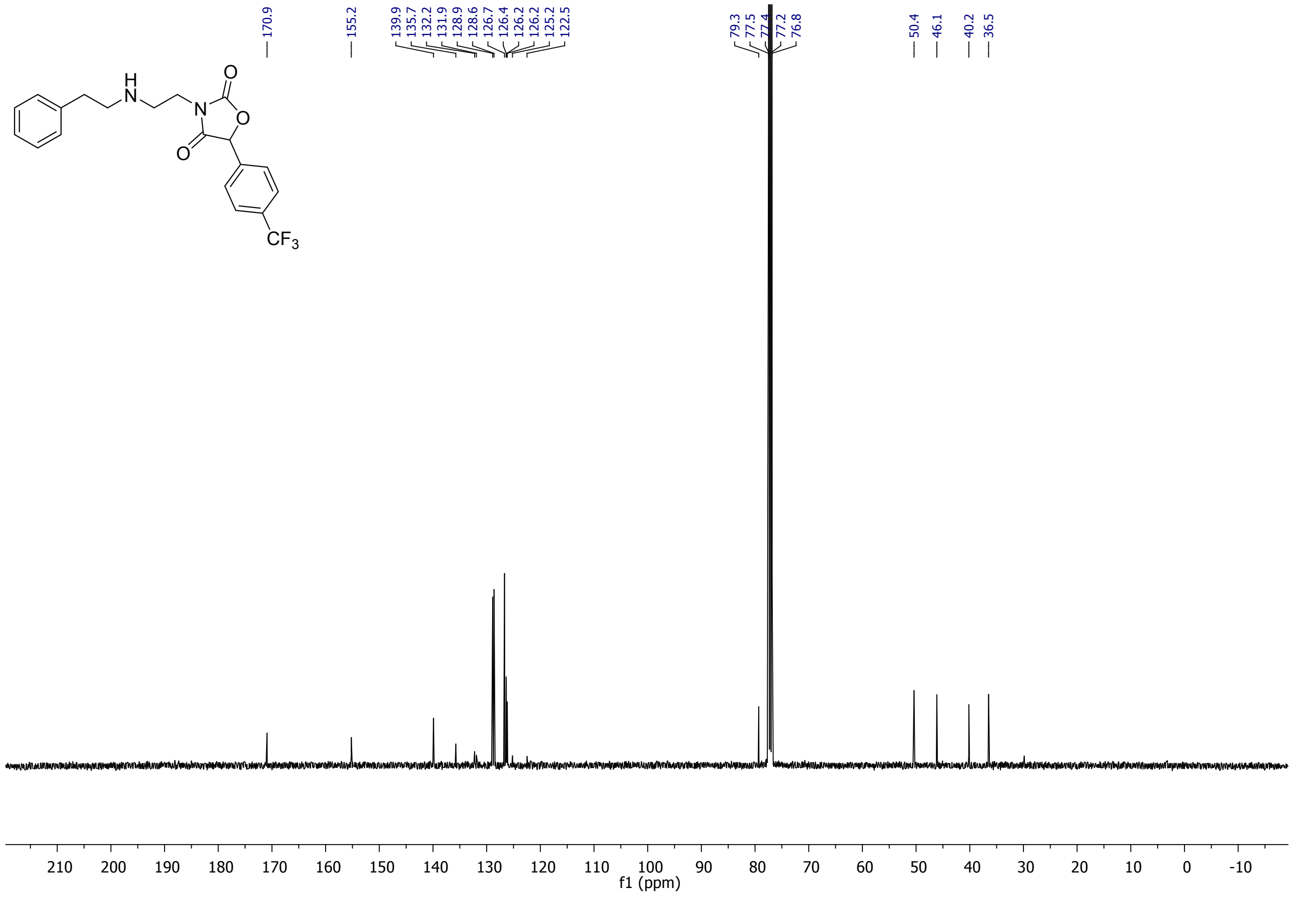
170.9
155.2
139.9 135.7 132.2 131.9 128.9 128.6 126.7 126.4 126.2 126.2 125.2 122.5
79.3 77.5 77.4 77.2 76.8
50.4
46.1
40.2
36.5
H
N
O
CF3
210 200 190 180 170 160 150 140 130 120 110 100 90 80 70 60 50 40 30 20 10 0 -10
f1 (ppm)

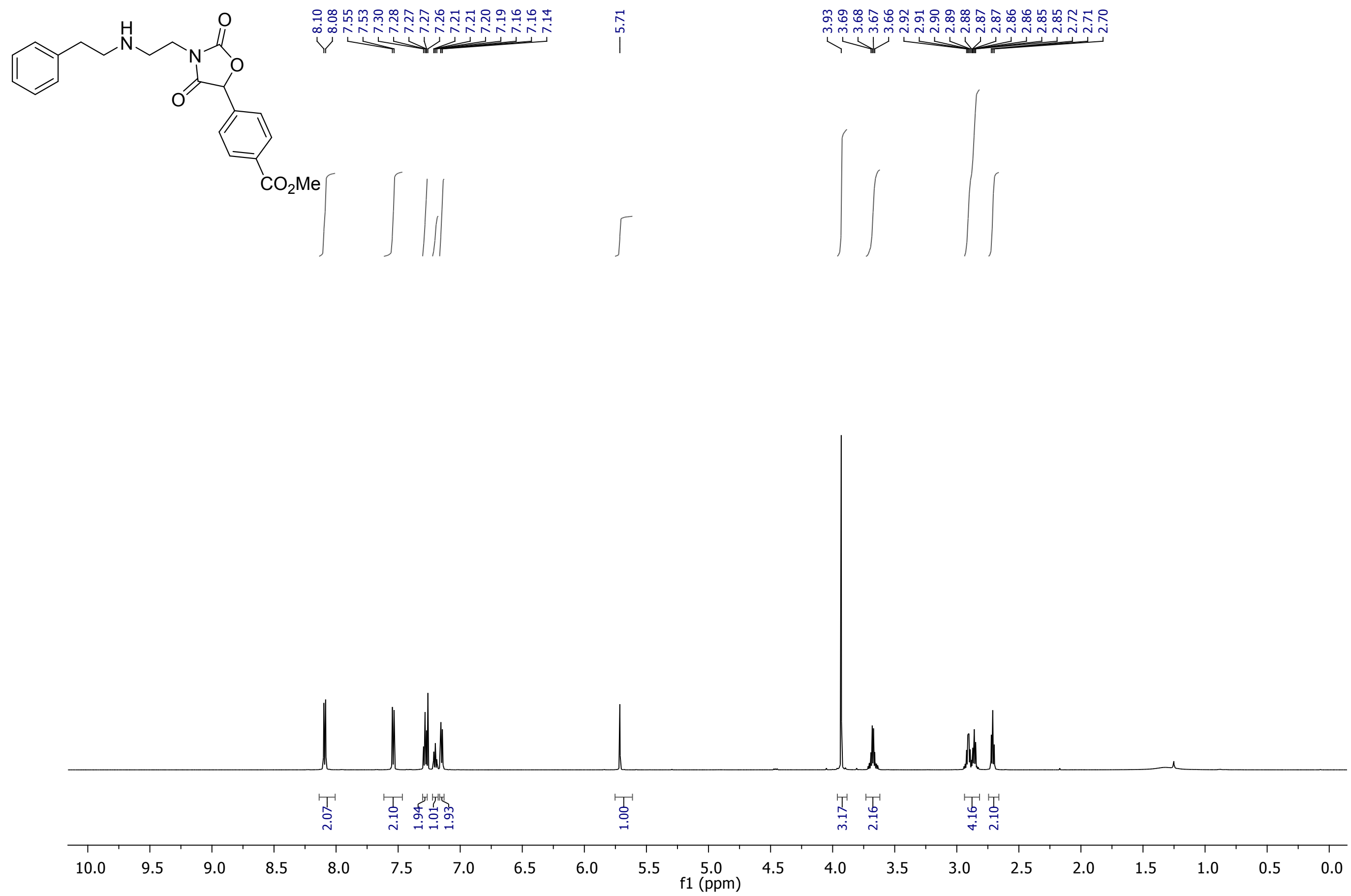
8.10
8.08
7.55
7.53
7.30
7.28
7.27
7.27
7.26
7.21
7.21
7.20
7.19
7.16
7.16
7.14
5.71
3.93
3.69
3.68
3.67
3.66
2.92
2.91
2.90
2.89
2.88
2.87
2.87
2.86
2.86
2.85
2.72
2.71
2.70
CO2Me
2.07
2.10
1.94
1.01
1.93
1.00
3.17
2.16
4.16
2.10
f1 (ppm)

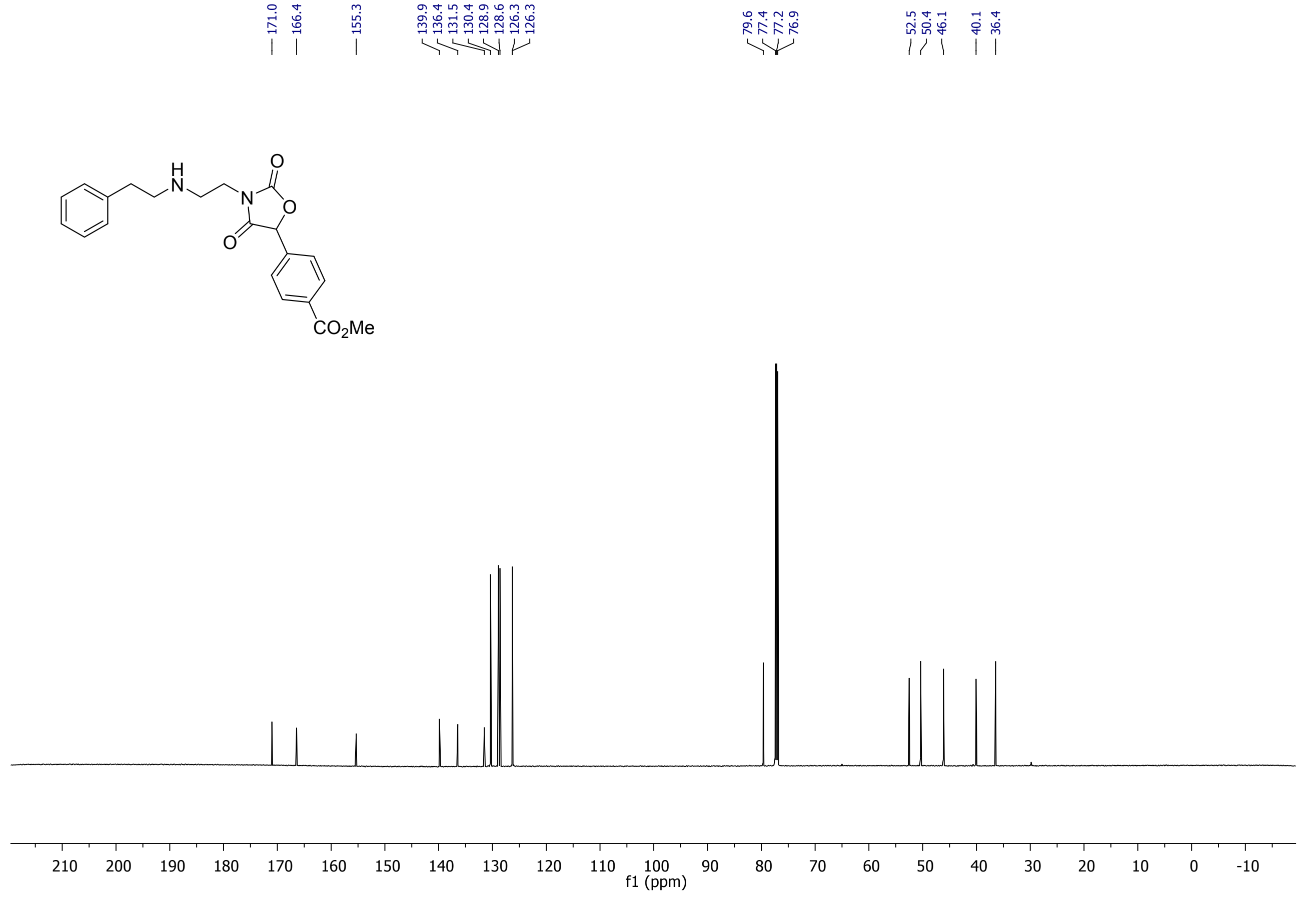
171.0
166.4
155.3
139.9
136.4
131.5
130.4
128.9
128.6
126.3
126.3
79.6
77.4
77.2
76.9
52.5
50.4
46.1
40.1
36.4
CO2Me
f1 (ppm)

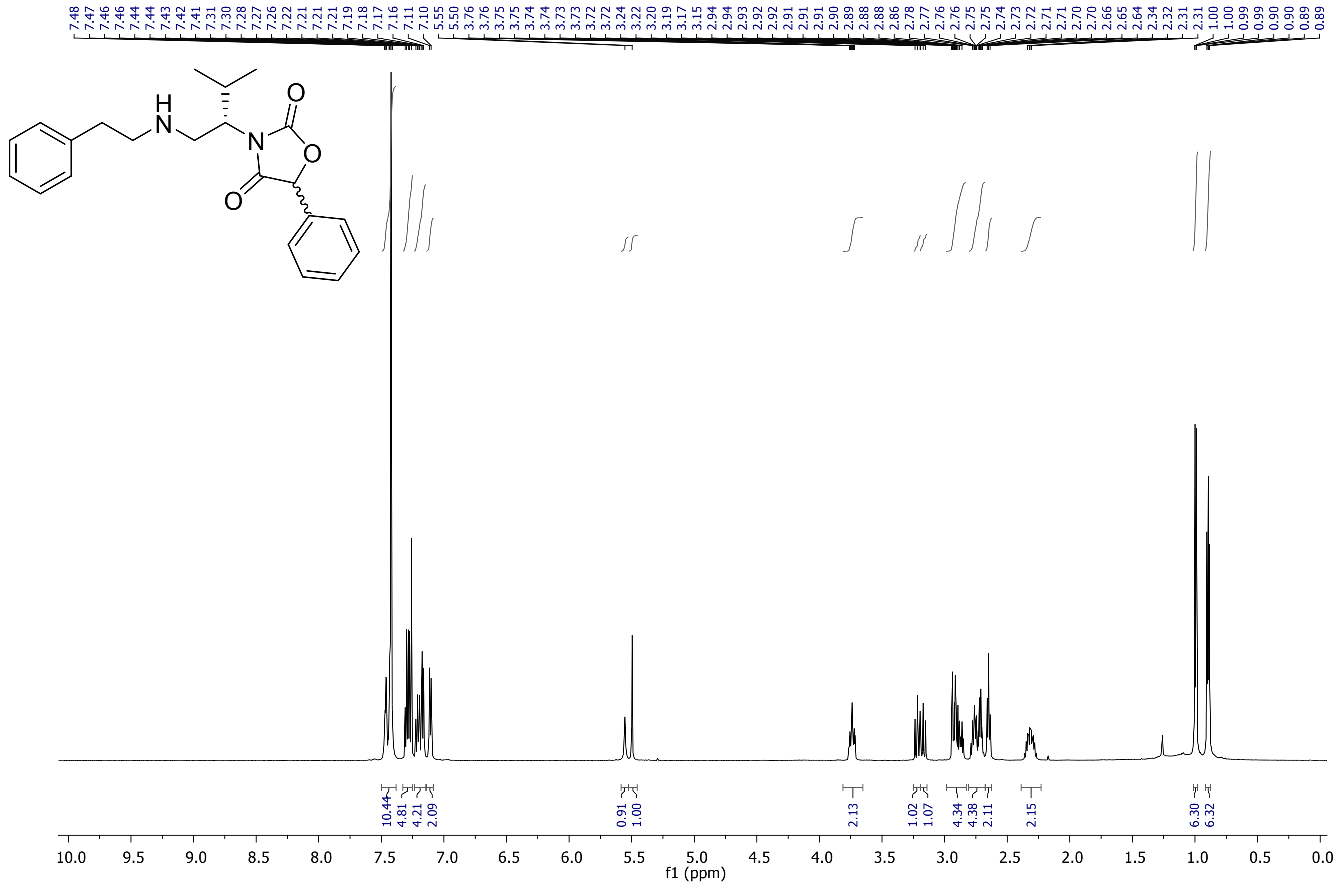
7.48 7.47 7.46 7.44 7.44 7.43 7.42 7.41 7.31 7.30 7.28 7.27 7.26 7.22 7.21 7.21 7.21 7.21 7.19 7.18 7.17 7.16 7.11 7.10 5.55 5.50 3.76 3.76 3.75 3.75 3.74 3.74 3.73 3.73 3.72 3.72 3.24 3.22 3.20 3.19 3.17 3.15 2.94 2.94 2.93 2.92 2.92 2.91 2.91 2.91 2.91 2.90 2.89 2.88 2.88 2.86 2.78 2.77 2.76 2.76 2.75 2.75 2.74 2.73 2.72 2.71 2.71 2.70 2.70 2.66 2.65 2.64 2.34 2.32 2.31 2.31 1.00 1.00 0.99 0.99 0.90 0.90 0.89 0.89
H
N
N
O
O
O
10.44
4.81
4.21
2.09
0.91
1.00
2.13
1.02
1.07
4.34
4.38
2.11
2.15
6.30
6.32
10.0 9.5 9.0 8.5 8.0 7.5 7.0 6.5 6.0 5.5 5.0 4.5 4.0 3.5 3.0 2.5 2.0 1.5 1.0 0.5 0.0
f1 (ppm)

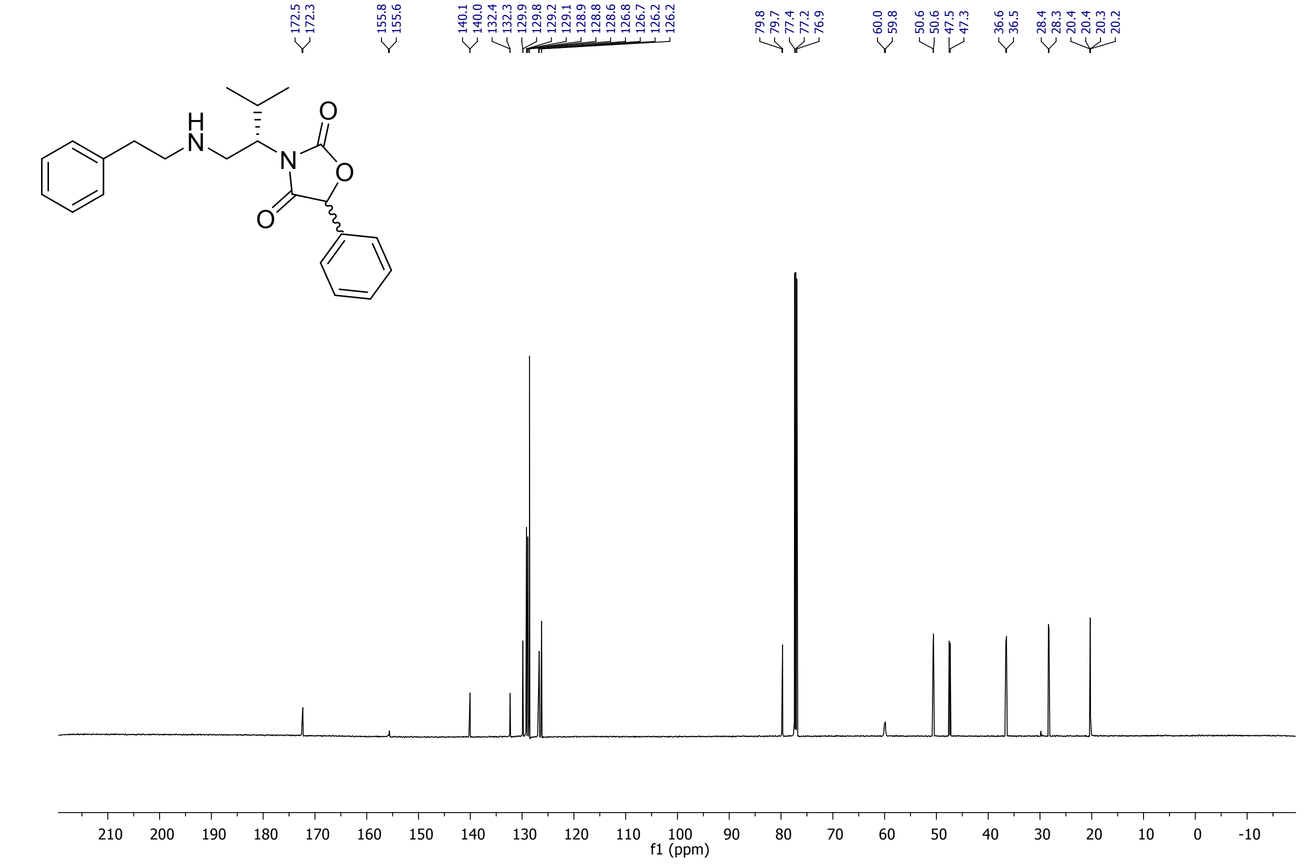
172.5 172.3 155.8 155.6 140.1 140.0 132.4 132.3 129.9 129.8 129.2 129.1 128.9 128.8 128.6 126.8 126.7 126.2 126.2 79.8 79.7 77.4 77.2 76.9 60.0 59.8 50.6 50.6 47.5 47.3 36.6 36.5 28.4 28.3 20.4 20.4 20.3 20.2
H
N
N
O
O
O
210 200 190 180 170 160 150 140 130 120 110 100 90 80 70 60 50 40 30 20 10 0 -10
f1 (ppm)

Determination of the diasteromeric ratio with internal standard (left)

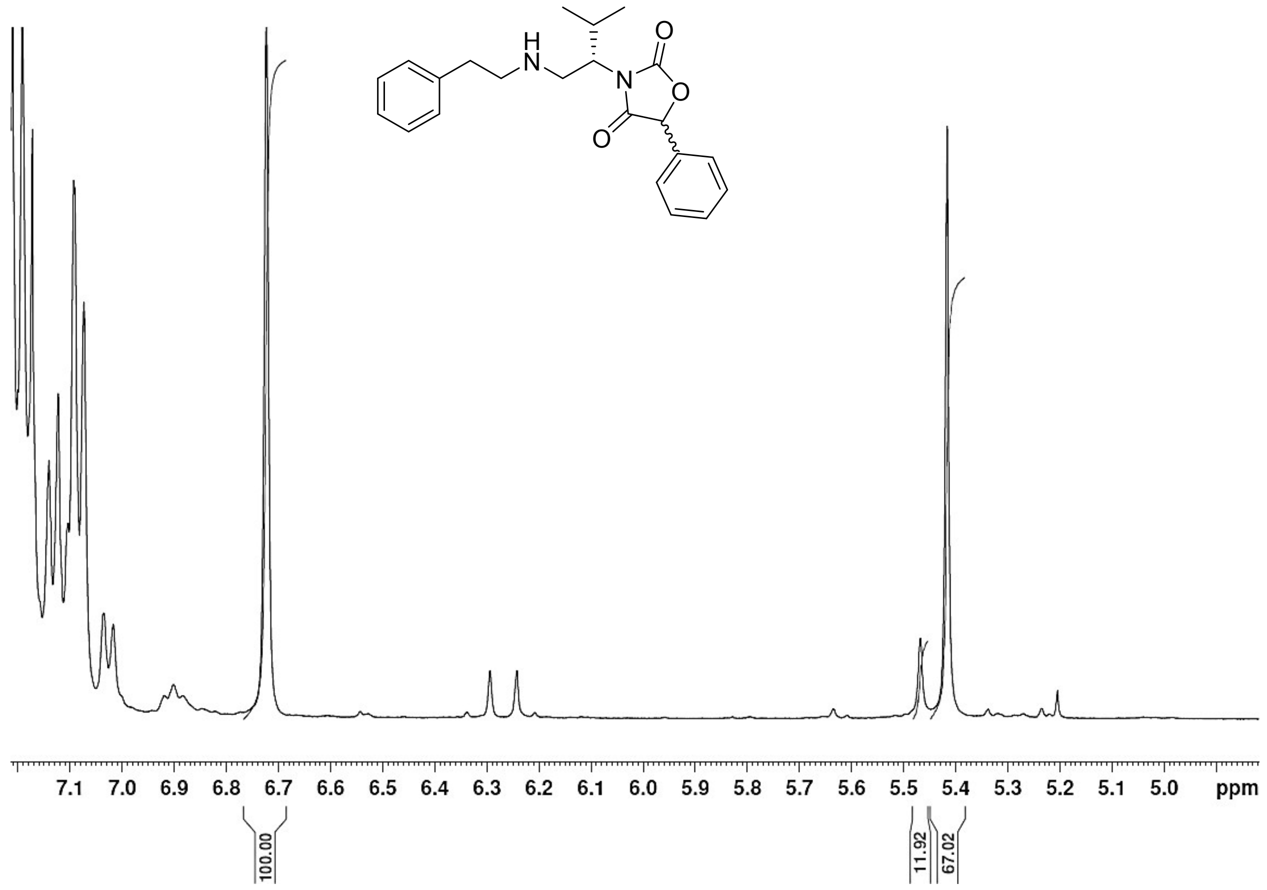

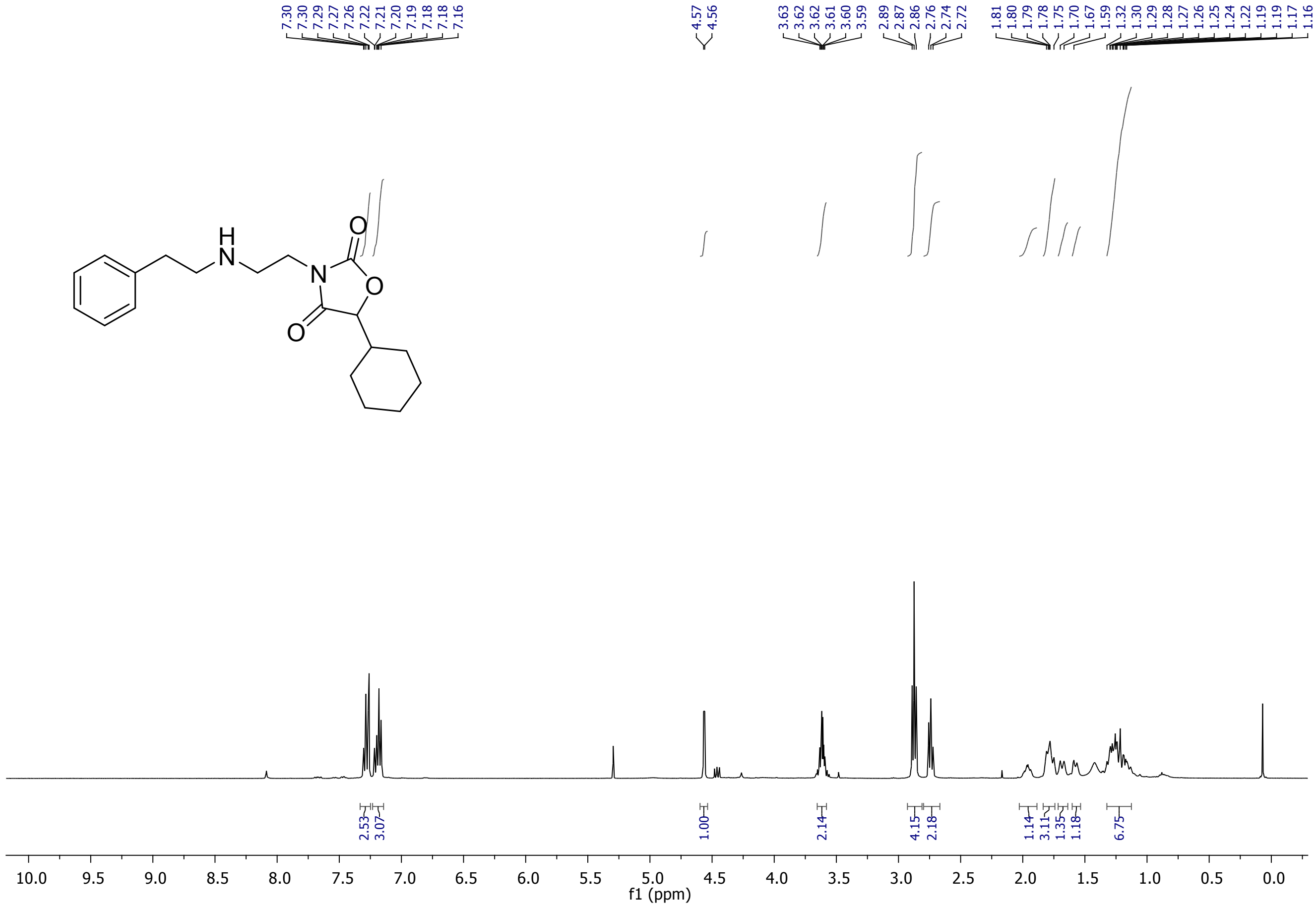

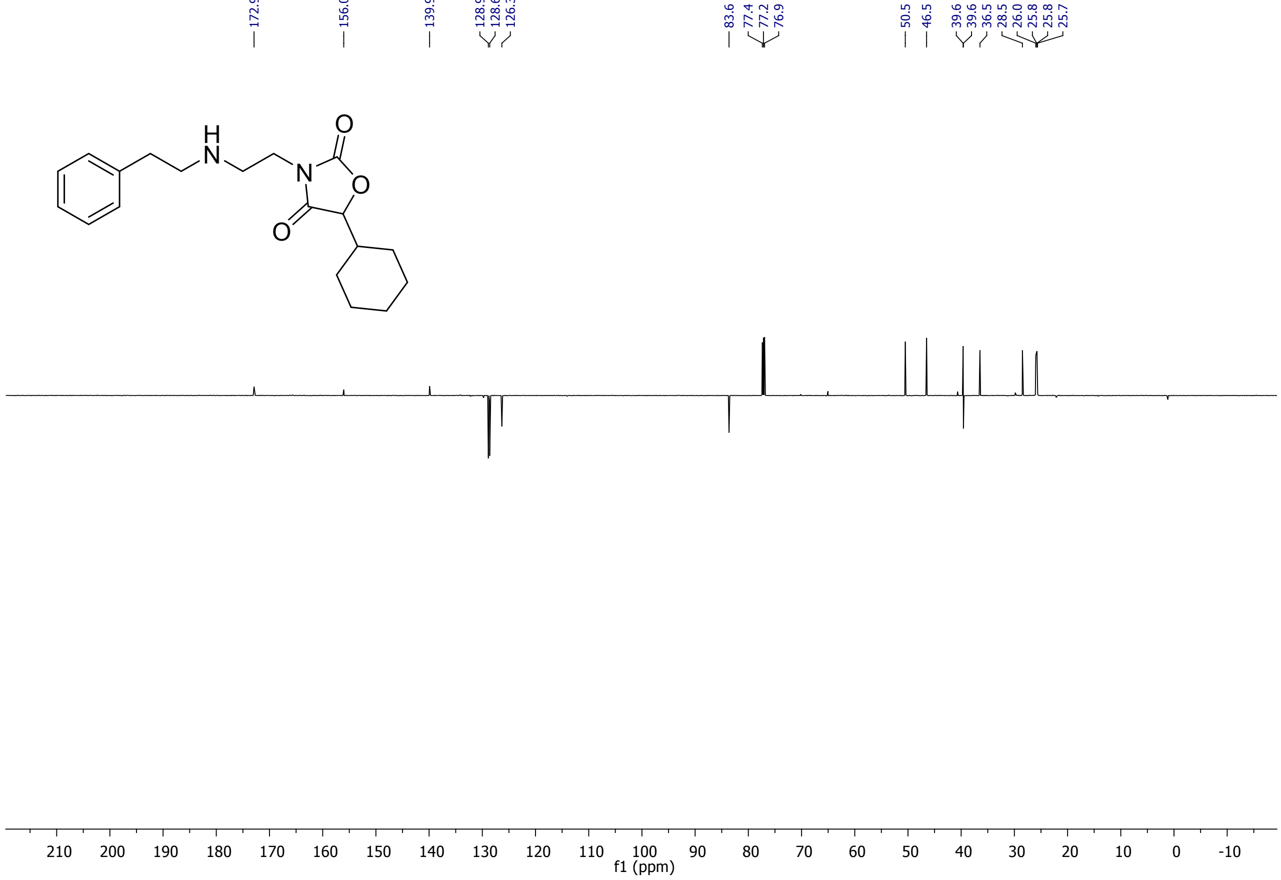

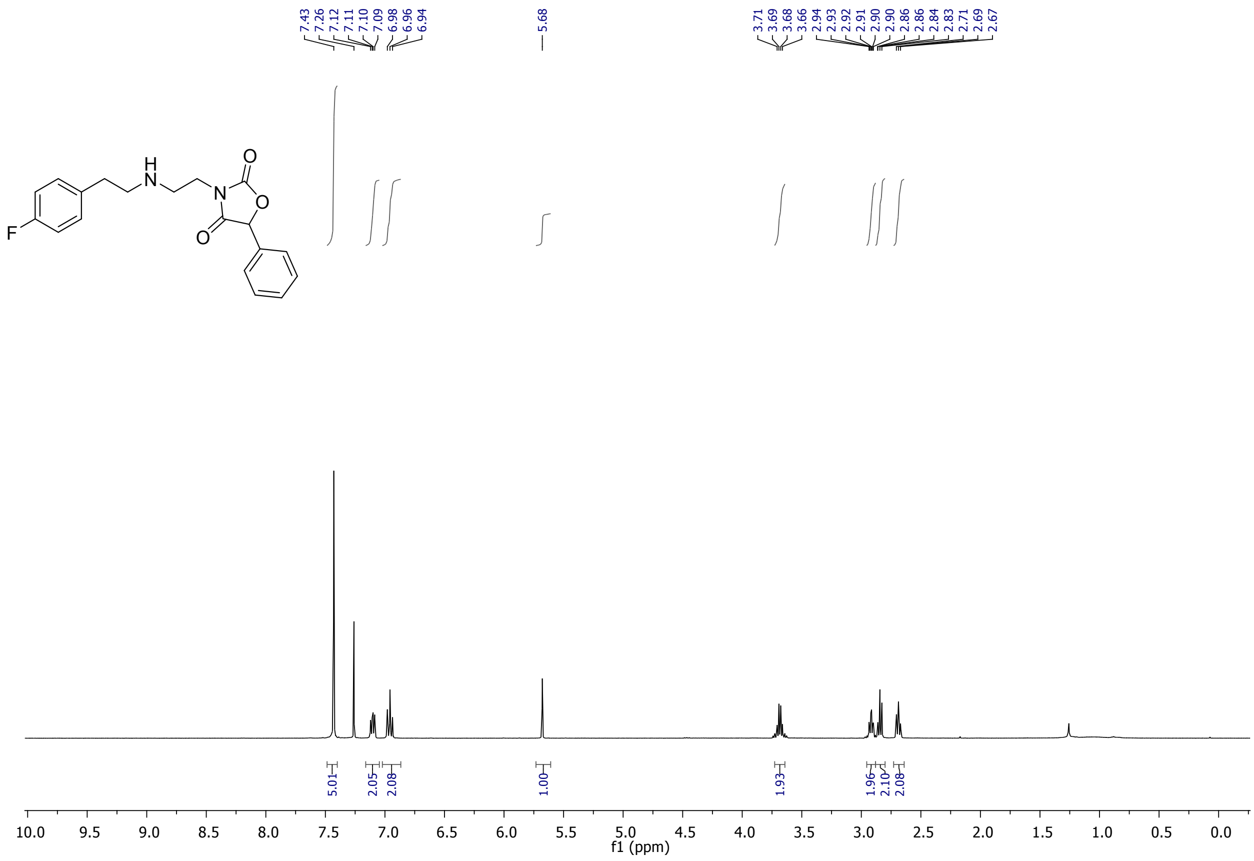

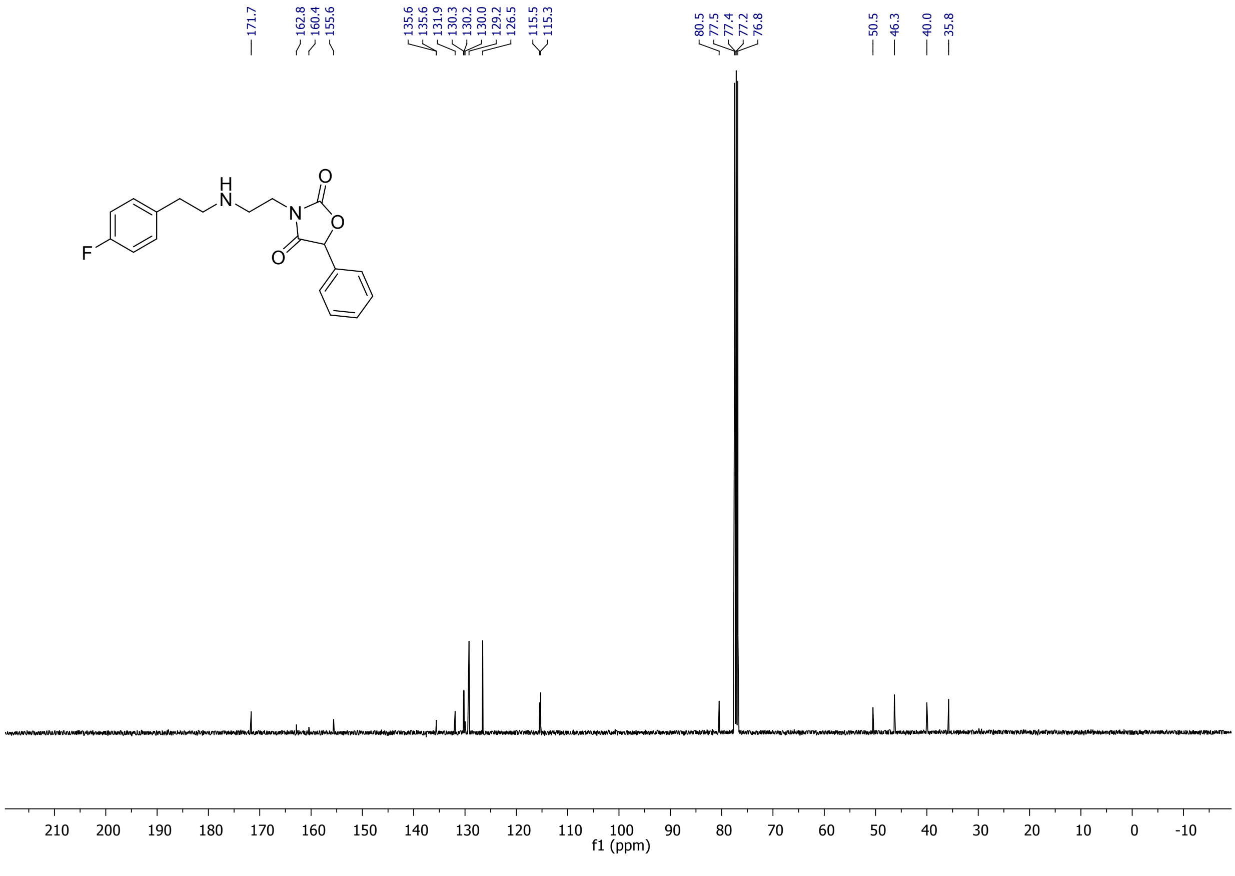

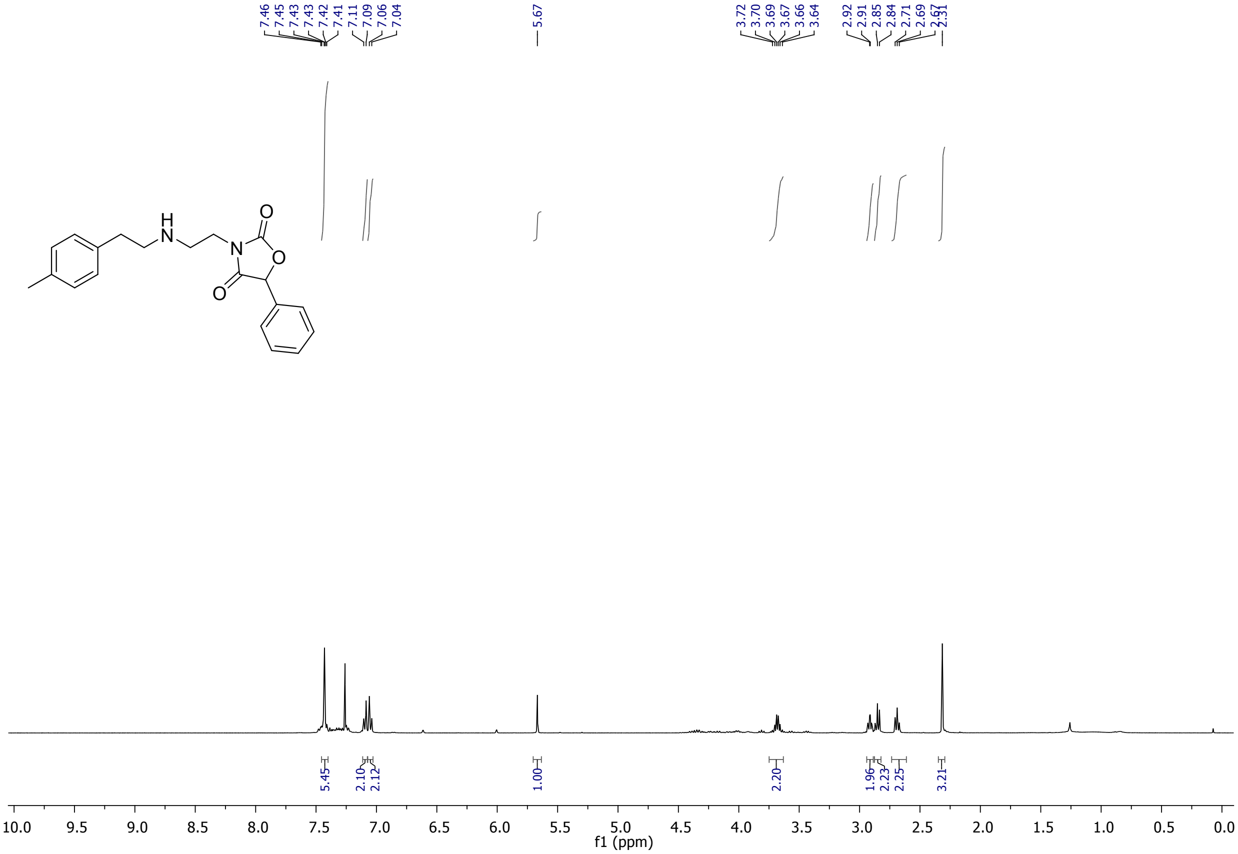
7.46 7.45 7.43 7.43 7.42 7.41 7.11 7.09 7.06 7.04
5.67
3.72 3.70 3.69 3.67 3.66 3.64
2.92 2.91 2.85 2.84 2.71 2.69 2.67 2.31
5.45 2.10 2.12 1.00 2.20 1.96 2.23 2.25 3.21
f1 (ppm)

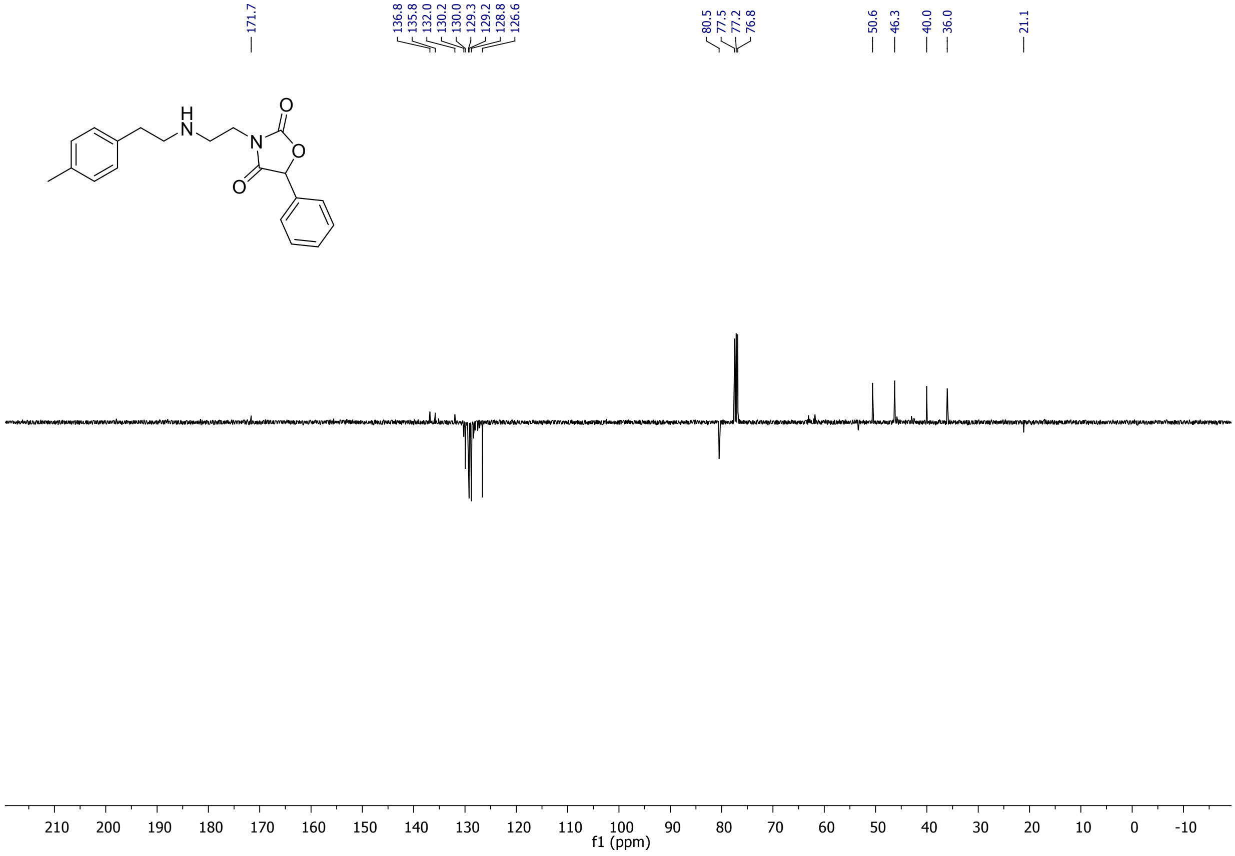
171.7
136.8 135.8 132.0 130.2 130.0 129.3 129.2 128.8 126.6
80.5 77.5 77.2 76.8
50.6 46.3 40.0 36.0 21.1
f1 (ppm)

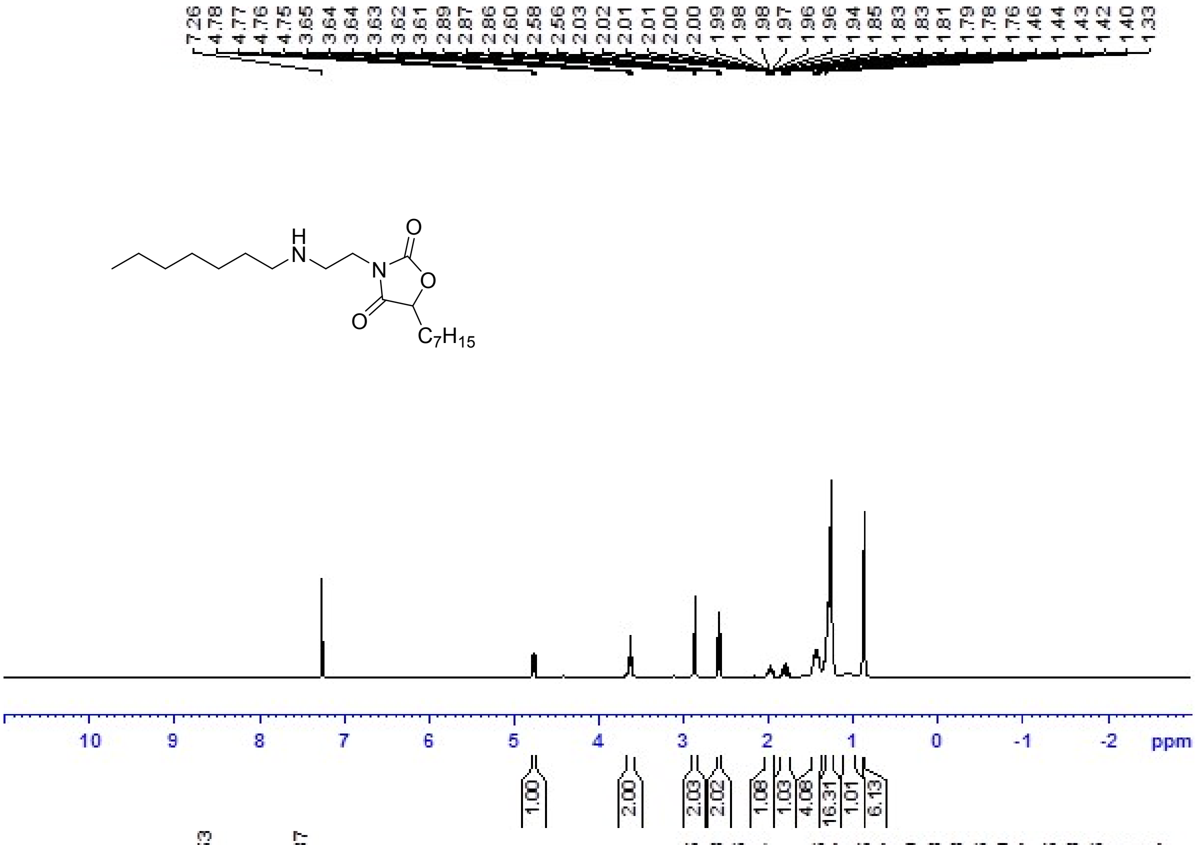

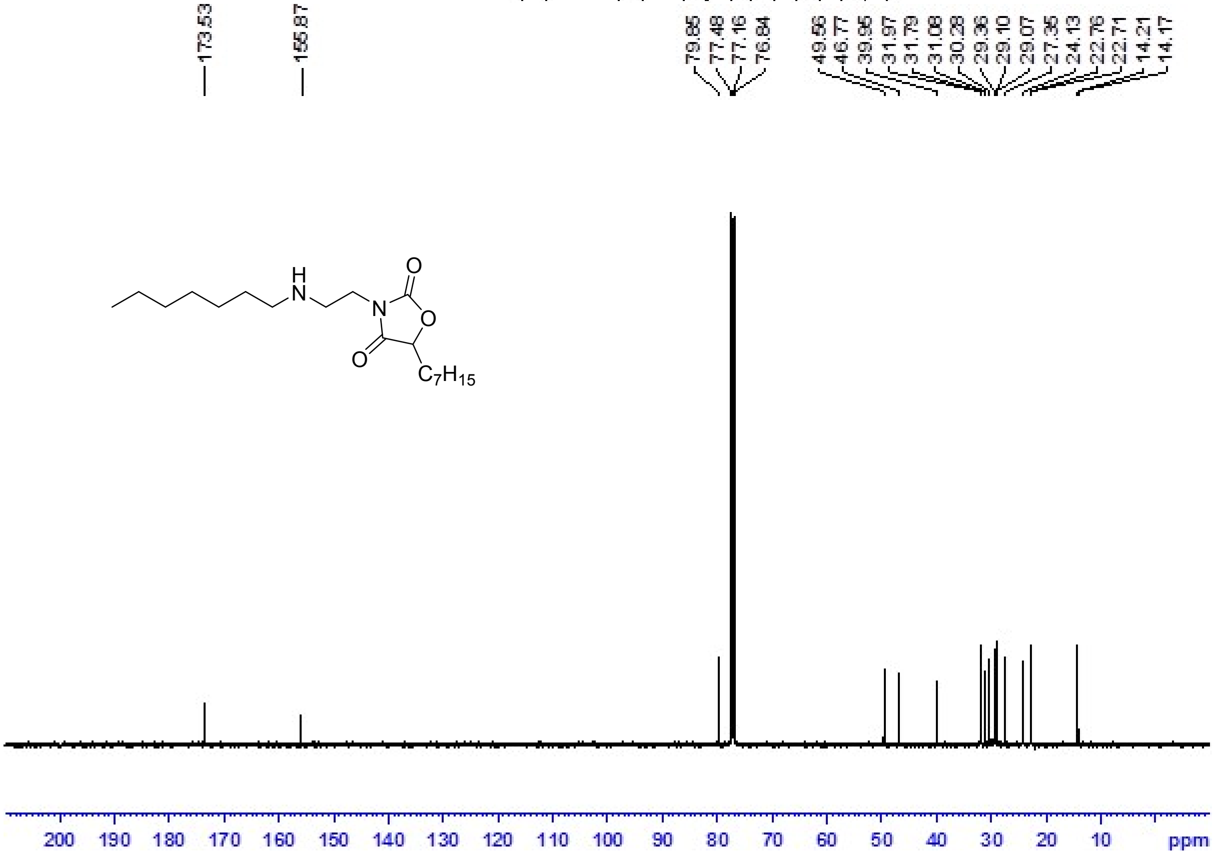

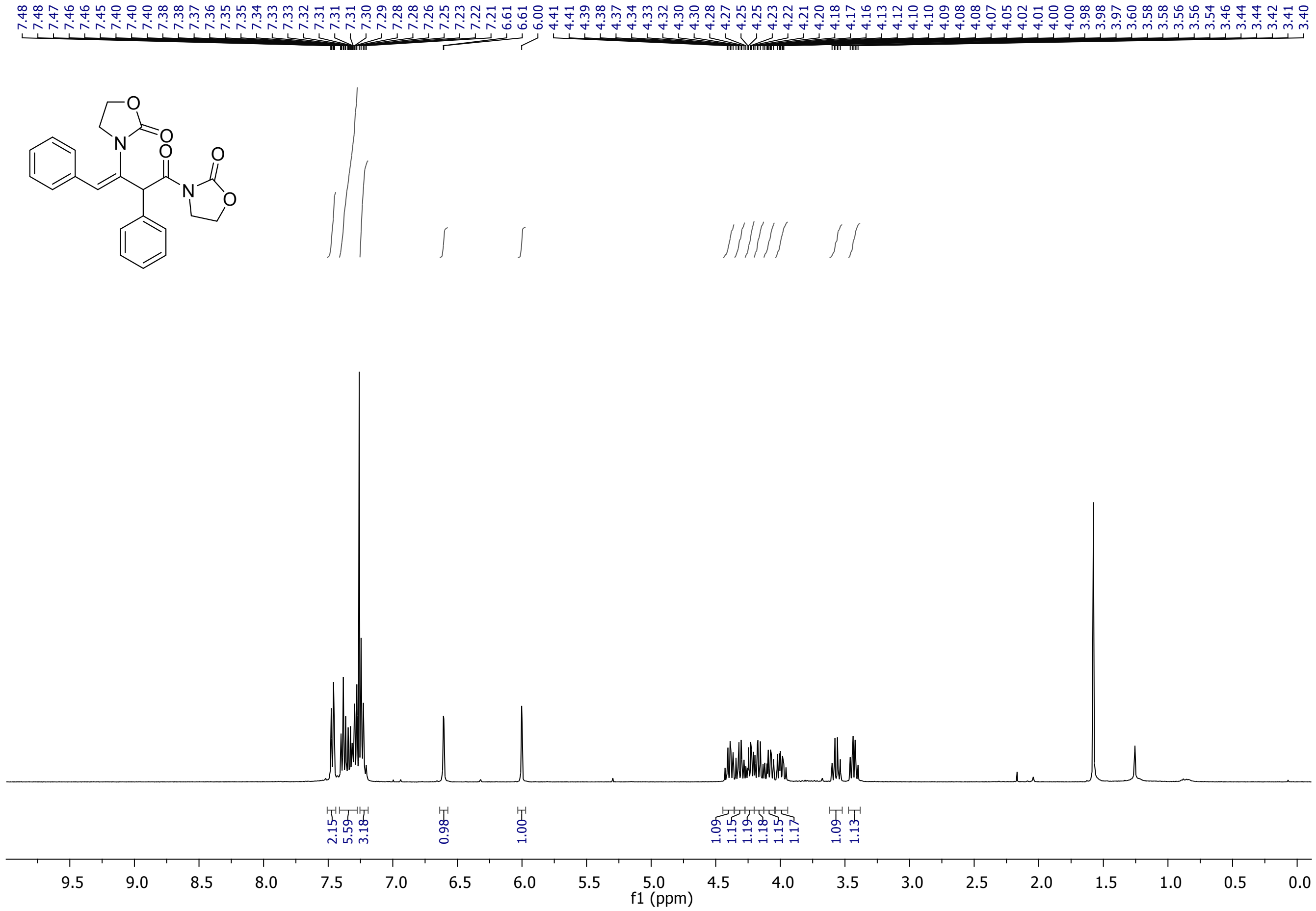
7.48 7.48 7.47 7.46 7.46 7.45 7.40 7.40 7.38 7.38 7.37 7.36 7.35 7.35 7.34 7.33 7.33 7.32 7.31 7.31 7.31 7.30 7.29 7.28 7.28 7.26 7.25 7.23 7.22 7.21 6.61 6.00 4.41 4.41 4.39 4.38 4.37 4.34 4.33 4.32 4.30 4.28 4.27 4.25 4.25 4.23 4.22 4.21 4.20 4.18 4.17 4.16 4.13 4.12 4.10 4.10 4.09 4.08 4.08 4.07 4.05 4.02 4.01 4.00 4.00 3.98 3.98 3.97 3.60 3.58 3.58 3.56 3.56 3.54 3.46 3.44 3.44 3.42 3.41 3.40
2.15 5.59 3.18 0.98 1.00 1.09 1.15 1.19 1.18 1.15 1.17 1.09 1.13
9.5 9.0 8.5 8.0 7.5 7.0 6.5 6.0 5.5 5.0 4.5 4.0 3.5 3.0 2.5 2.0 1.5 1.0 0.5 0.0
f1 (ppm)

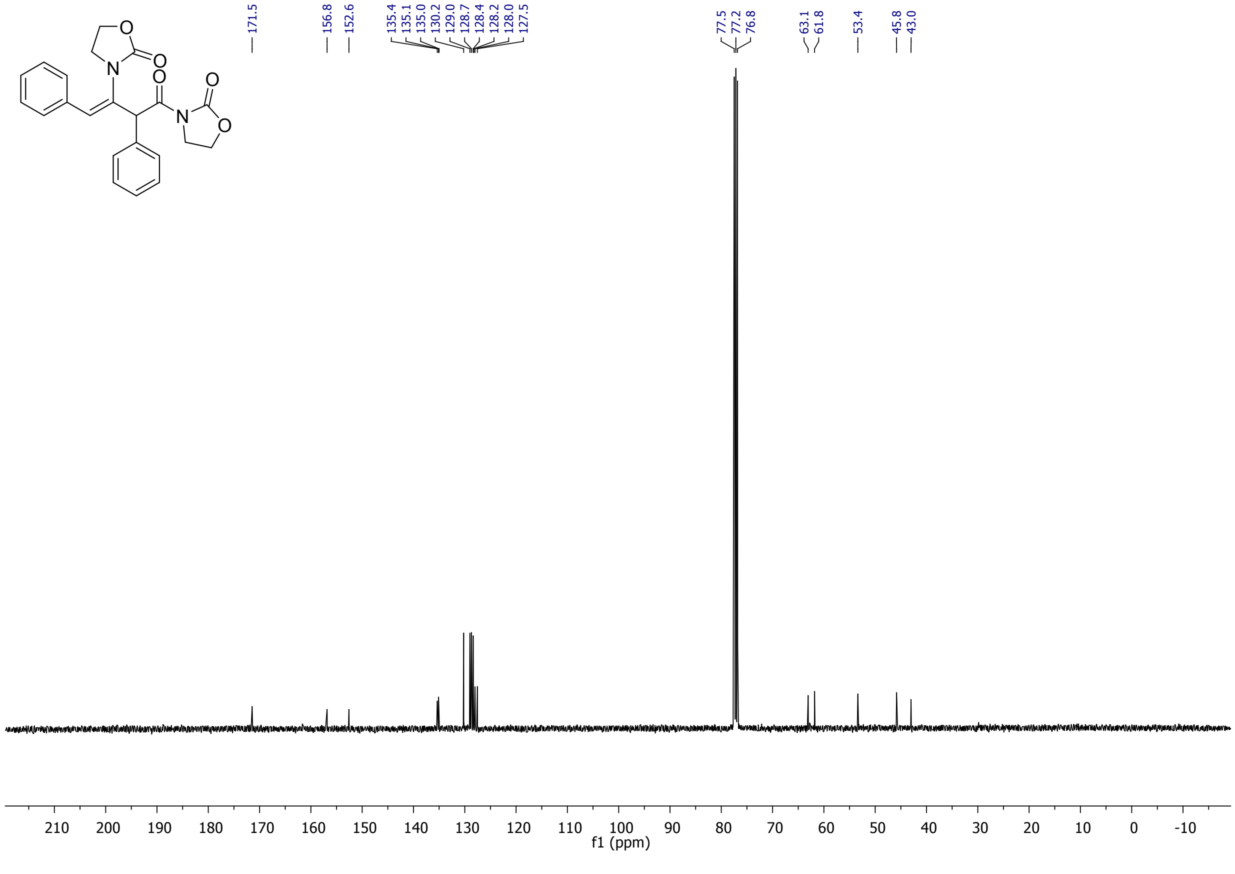
171.5 156.8 152.6 135.4 135.1 135.0 130.2 129.0 128.7 128.4 128.2 128.0 127.5 77.5 77.2 76.8 63.1 61.8 53.4 45.8 43.0
210 200 190 180 170 160 150 140 130 120 110 100 90 80 70 60 50 40 30 20 10 0 -10
f1 (ppm)

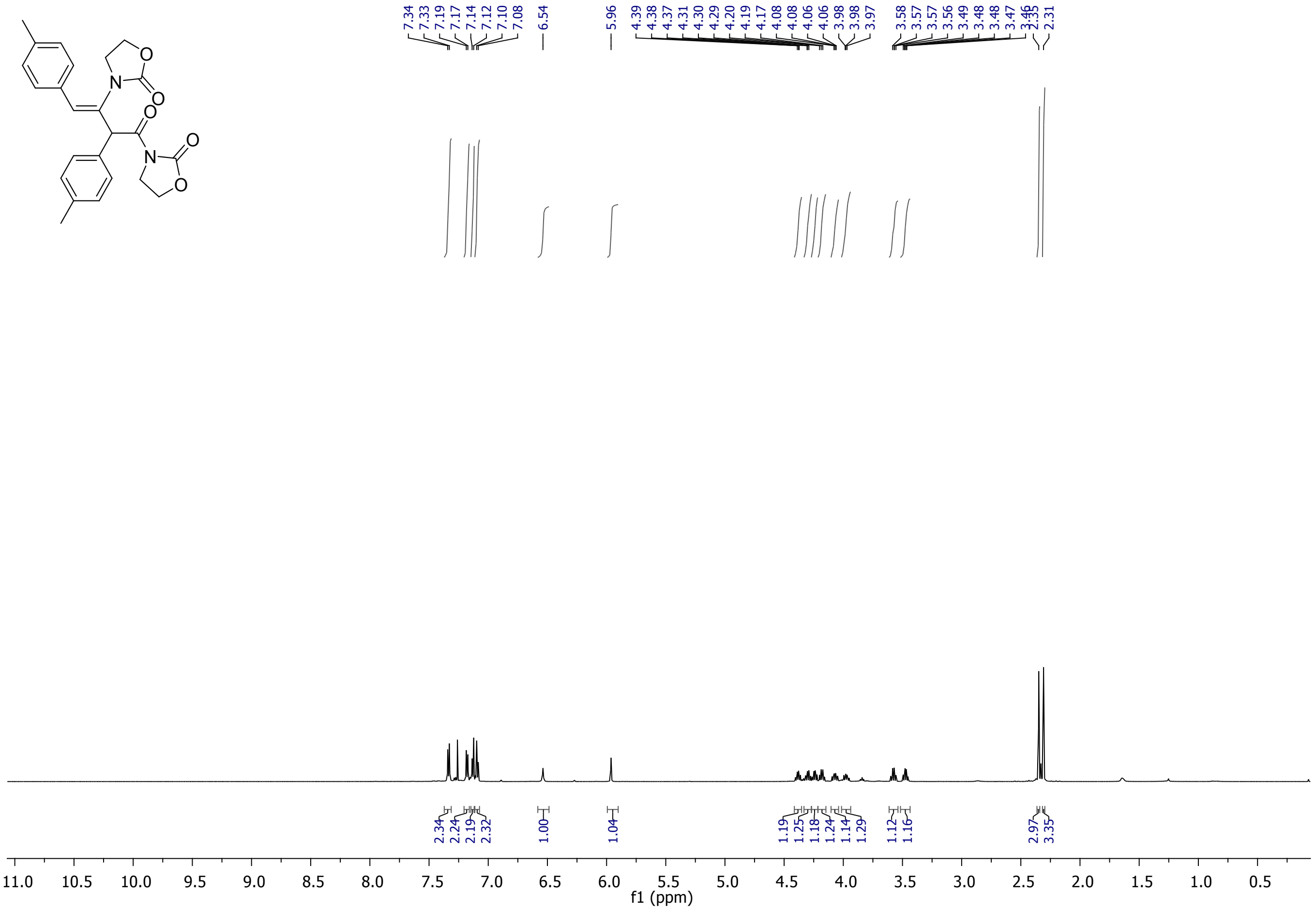

7.34
7.33
7.19
7.17
7.14
7.12
7.10
7.08
6.54
5.96
4.39
4.38
4.37
4.31
4.30
4.29
4.20
4.19
4.17
4.08
4.08
4.06
4.06
3.98
3.98
3.97
3.58
3.57
3.57
3.56
3.49
3.48
3.48
3.47
3.46
2.35
2.31
2.34
2.24
2.19
2.32
1.00
1.04
1.19
1.25
1.18
1.24
1.14
1.29
1.12
1.16
2.97
3.35
f1 (ppm)

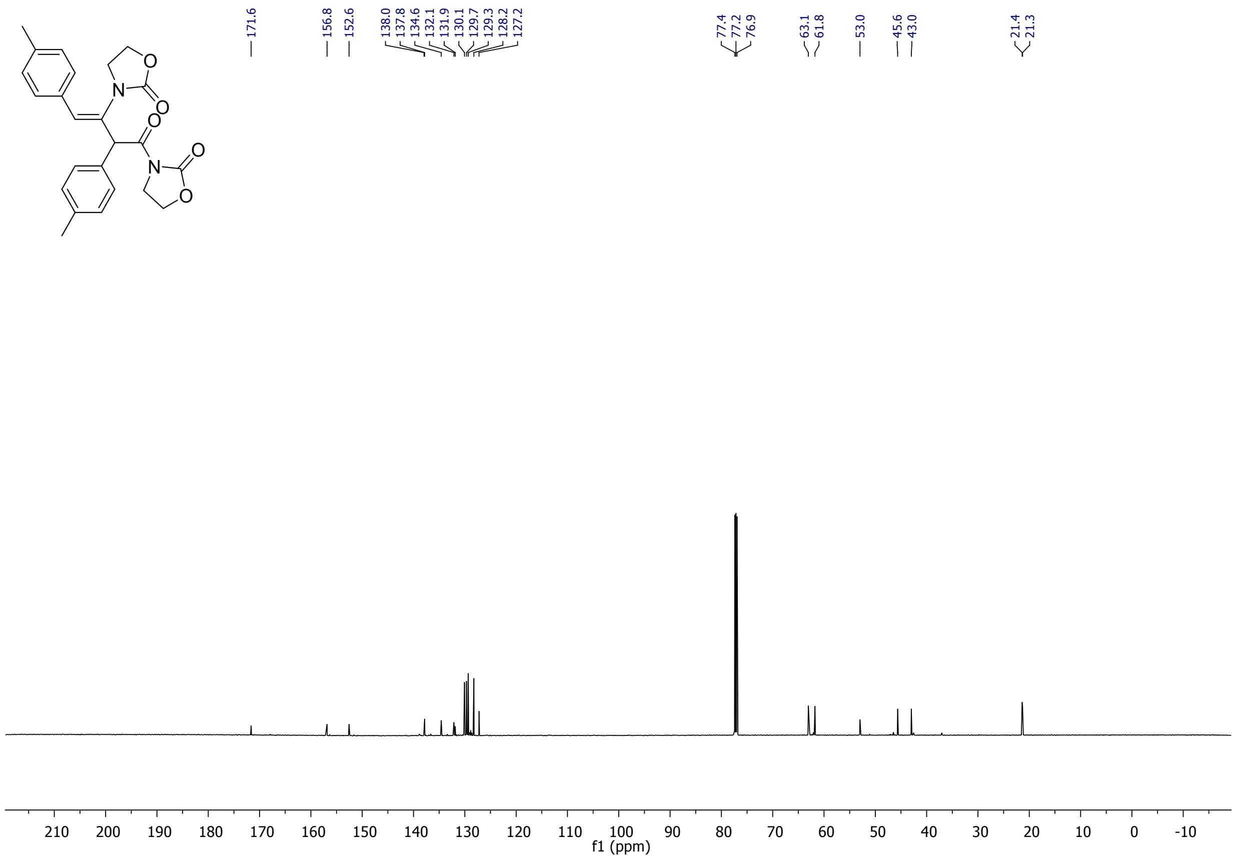

171.6
156.8
152.6
138.0
137.8
134.6
132.1
131.9
130.1
129.7
129.3
128.2
127.2
77.4
77.2
76.9
63.1
61.8
53.0
45.6
43.0
21.4
21.3
f1 (ppm)

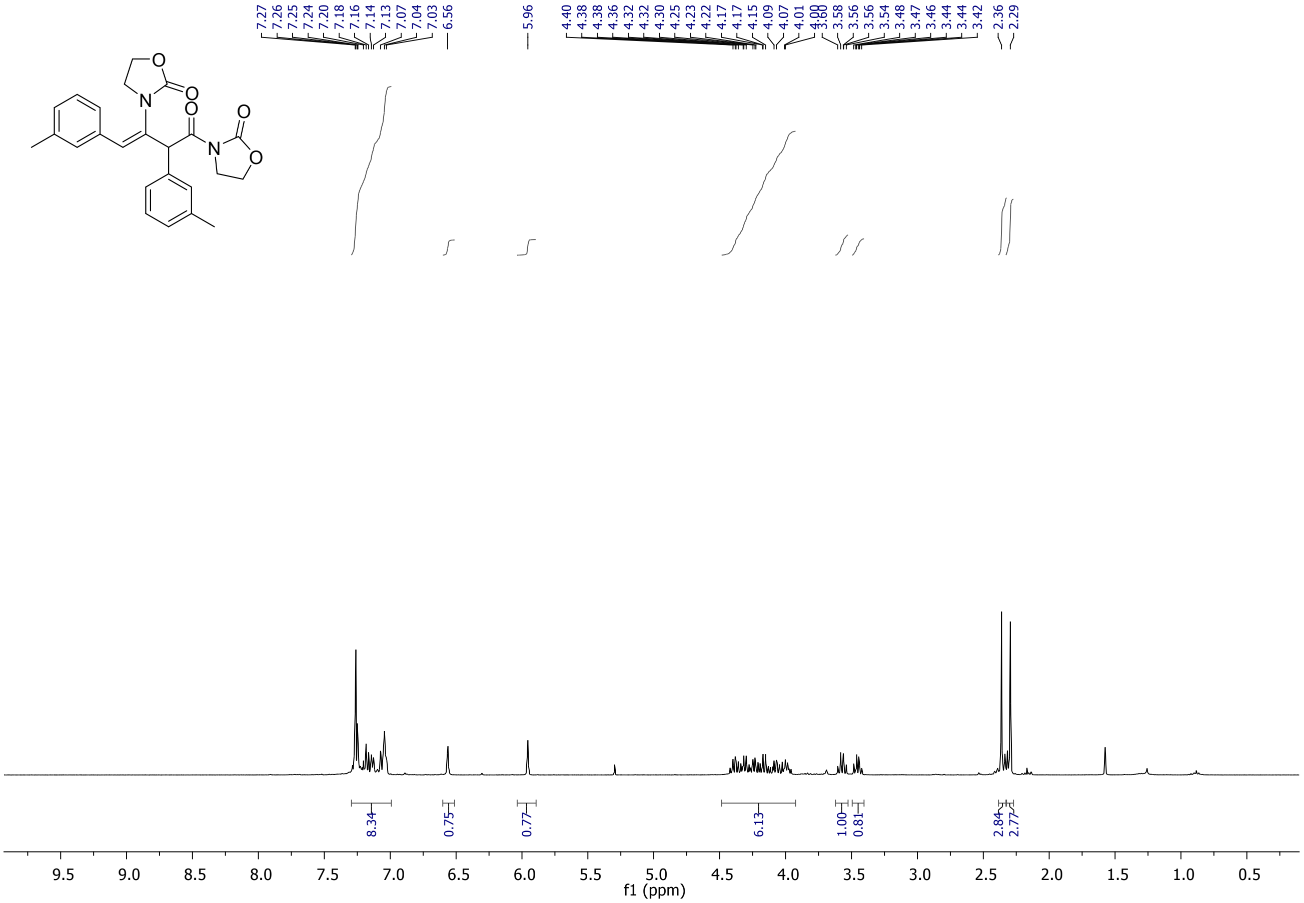

7.27 7.26 7.25 7.24 7.20 7.18 7.16 7.14 7.13 7.07 7.04 7.03 6.56
5.96
4.40 4.38 4.38 4.36 4.32 4.32 4.30 4.25 4.23 4.22 4.17 4.17 4.15 4.09 4.07 4.01
3.58 3.56 3.56 3.54 3.48 3.47 3.46 3.44 3.44 3.42
2.36 2.29
8.34 0.75 0.77 6.13 1.00 0.81 2.84 2.77
9.5 9.0 8.5 8.0 7.5 7.0 6.5 6.0 5.5 5.0 4.5 4.0 3.5 3.0 2.5 2.0 1.5 1.0 0.5
f1 (ppm)

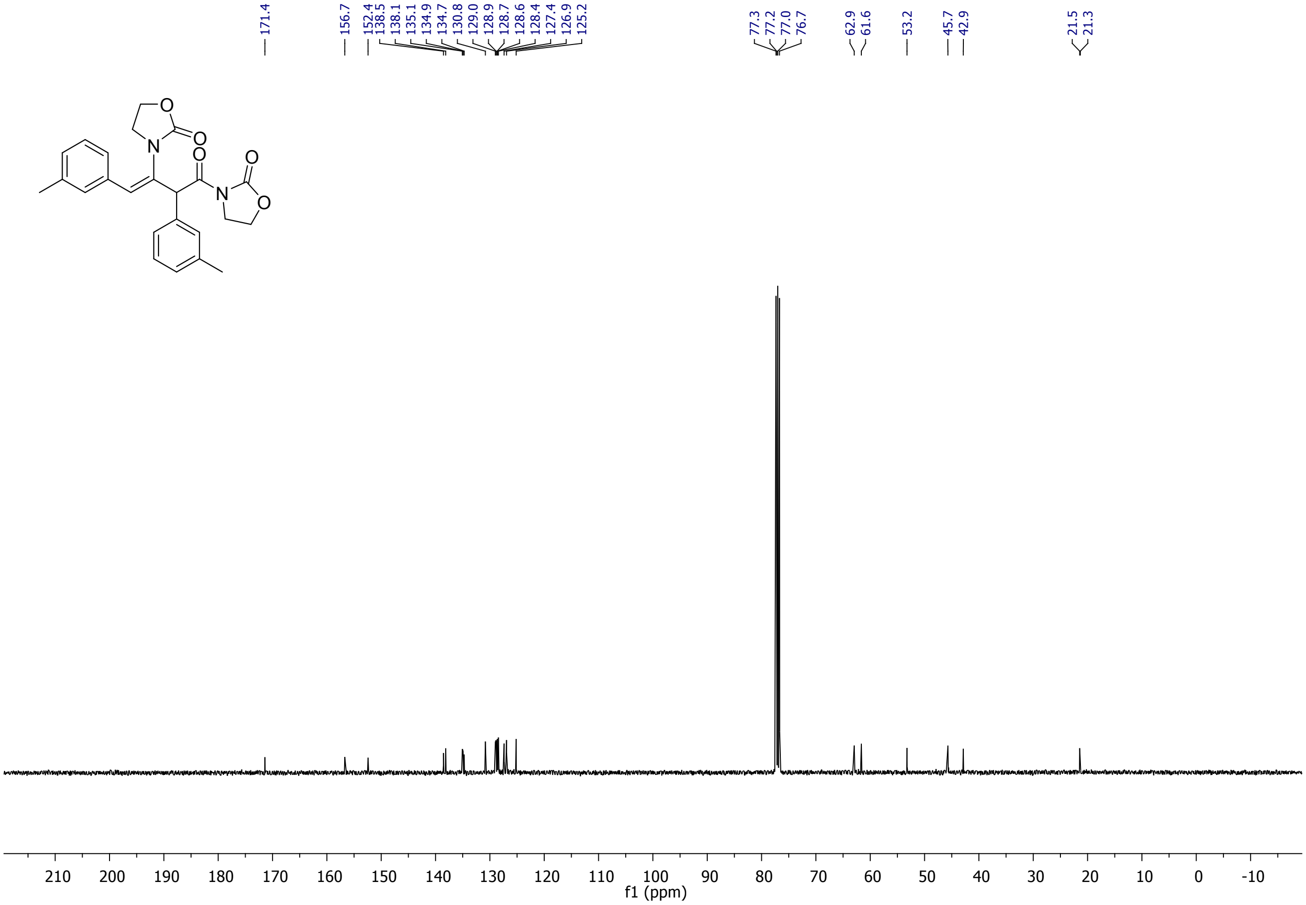

171.4
156.7 152.4 138.5 138.1 135.1 134.9 134.7 130.8 129.0 128.9 128.7 128.6 128.4 127.4 126.9 125.2
77.3 77.2 77.0 76.7
62.9 61.6
53.2
45.7 42.9
21.5 21.3
210 200 190 180 170 160 150 140 130 120 110 100 90 80 70 60 50 40 30 20 10 0 -10
f1 (ppm)

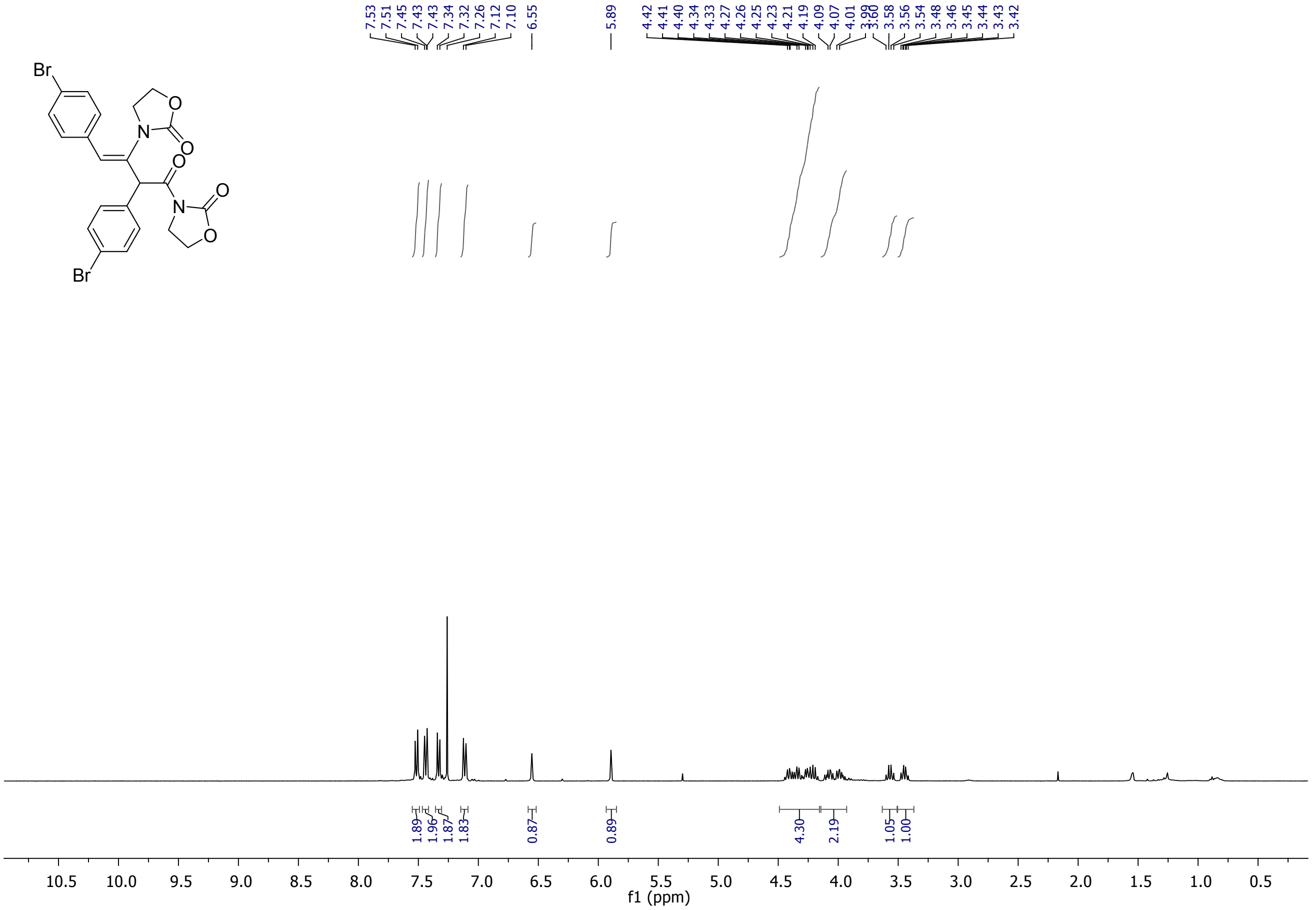
7.53
7.51
7.45
7.43
7.43
7.34
7.32
7.26
7.12
7.10
6.55
5.89
4.42
4.41
4.40
4.34
4.33
4.27
4.26
4.25
4.23
4.21
4.19
4.09
4.07
4.01
3.99
3.60
3.58
3.56
3.54
3.48
3.46
3.45
3.44
3.43
3.42
1.89
1.96
1.87
1.83
0.87
0.89
4.30
2.19
1.05
1.00
f1 (ppm)

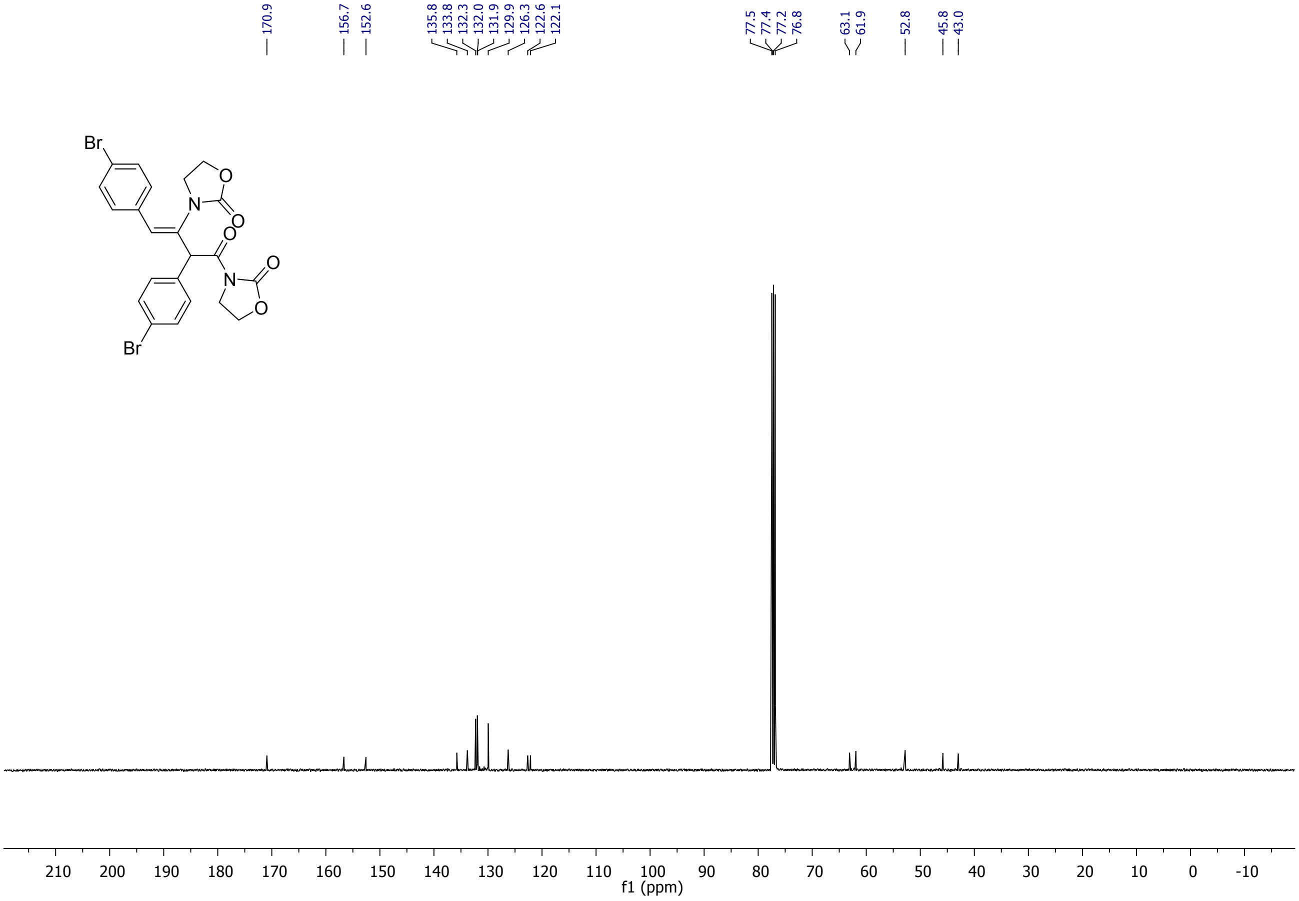
170.9
156.7
152.6
135.8
133.8
132.3
132.0
131.9
129.9
126.3
122.6
122.1
77.5
77.4
77.2
76.8
63.1
61.9
52.8
45.8
43.0
f1 (ppm)

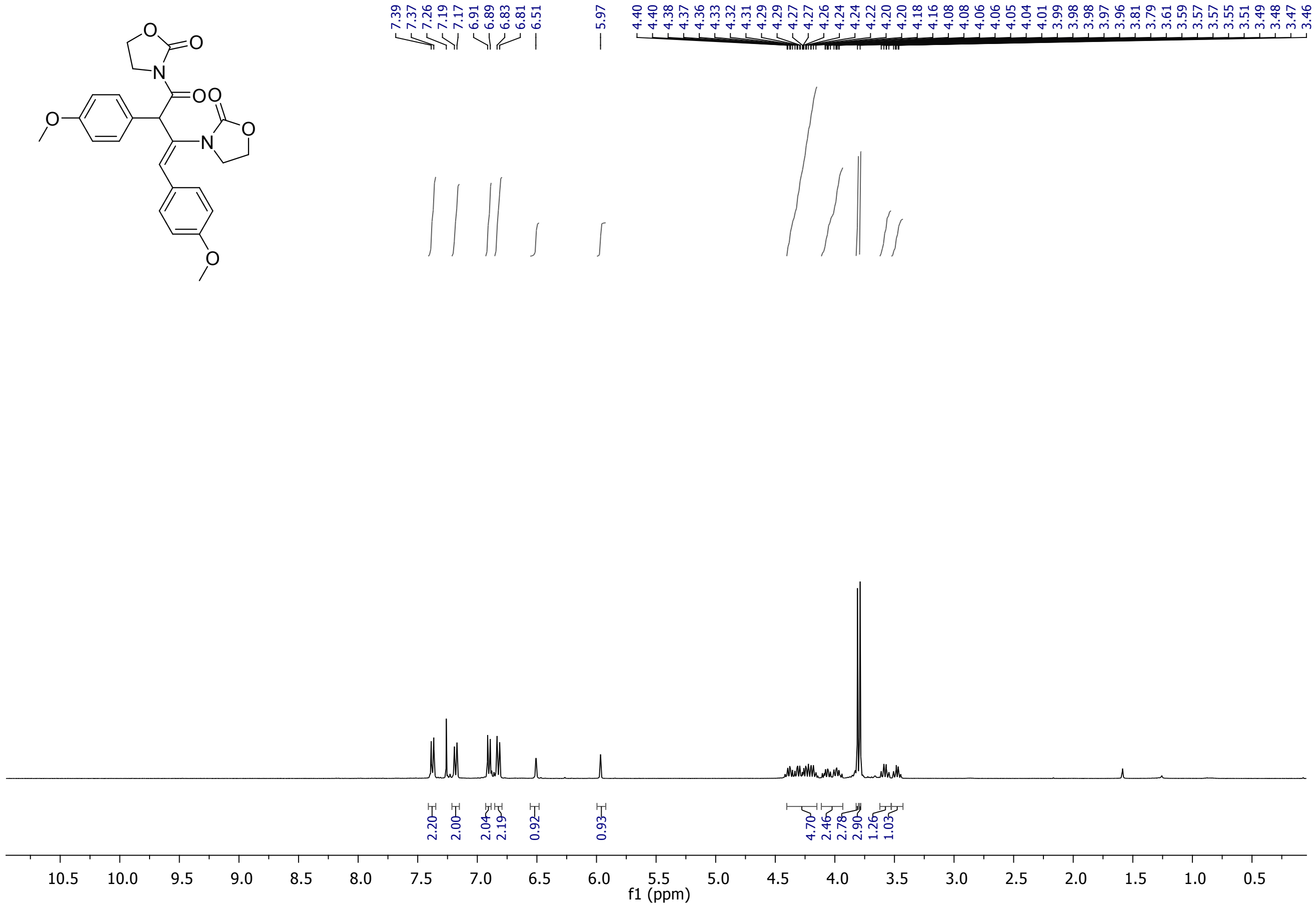
7.39
7.37
7.26
7.19
7.17
6.91
6.89
6.83
6.81
6.51
5.97
4.40
4.40
4.38
4.37
4.36
4.33
4.32
4.31
4.29
4.29
4.27
4.27
4.26
4.24
4.24
4.22
4.20
4.20
4.18
4.16
4.08
4.08
4.06
4.06
4.05
4.04
4.01
3.99
3.98
3.98
3.97
3.96
3.81
3.79
3.61
3.59
3.57
3.57
3.55
3.51
3.49
3.48
3.47
3.46
2.20
2.00
2.04
2.19
0.92
0.93
4.70
2.46
2.78
2.90
1.26
1.03
10.5 10.0 9.5 9.0 8.5 8.0 7.5 7.0 6.5 6.0 5.5 5.0 4.5 4.0 3.5 3.0 2.5 2.0 1.5 1.0 0.5
f1 (ppm)

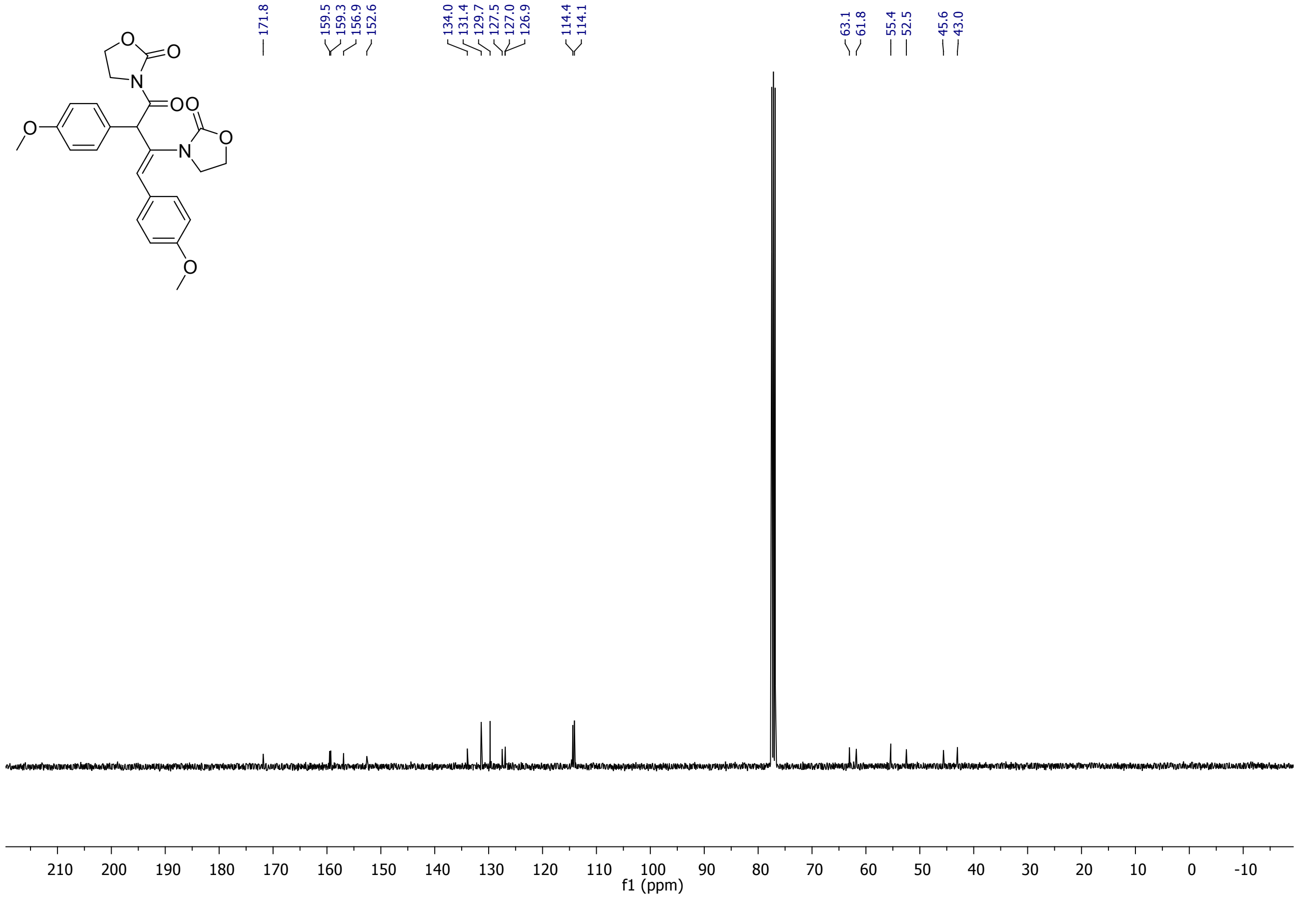
171.8
159.5
159.3
156.9
152.6
134.0
131.4
129.7
127.5
127.0
126.9
114.4
114.1
63.1
61.8
55.4
52.5
45.6
43.0
210 200 190 180 170 160 150 140 130 120 110 100 90 80 70 60 50 40 30 20 10 0 -10
f1 (ppm)

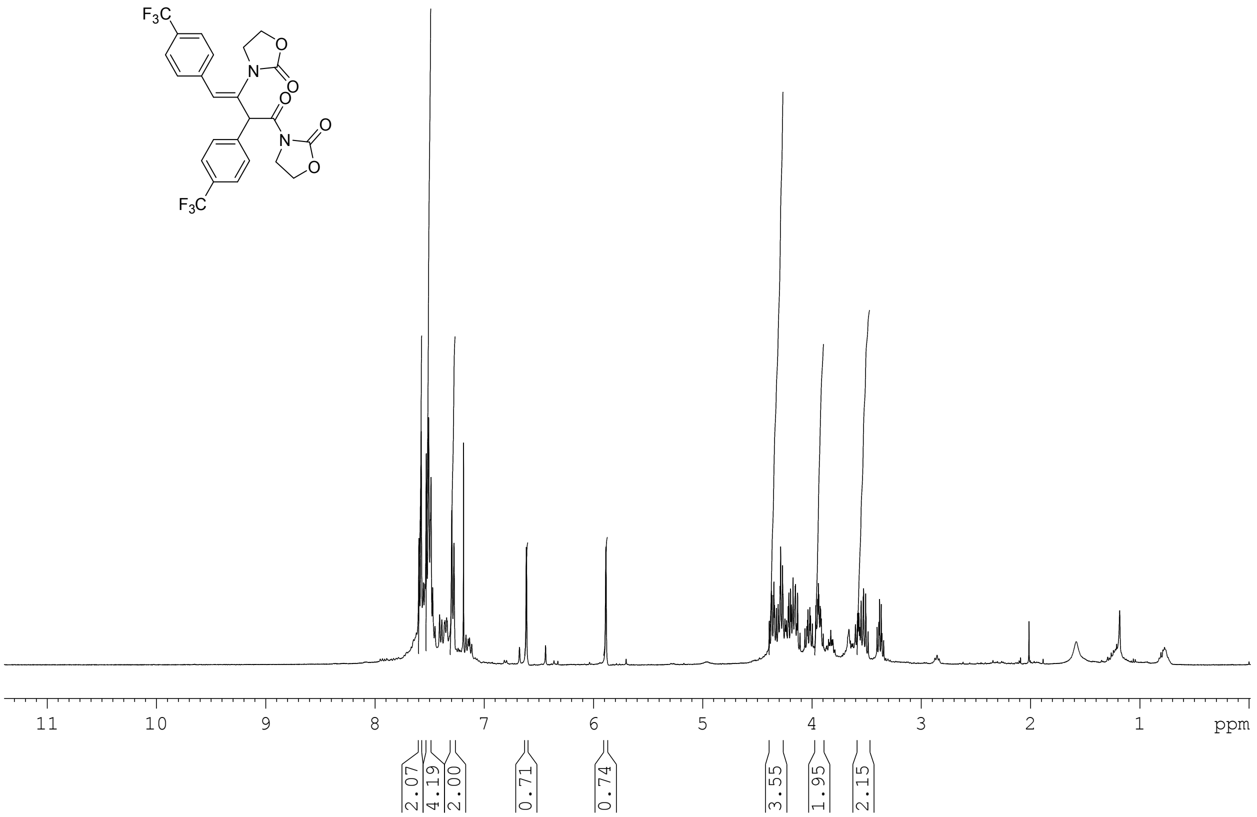
F3C
F3C
ppm
2.07
4.19
2.00
0.71
0.74
3.55
1.95
2.15

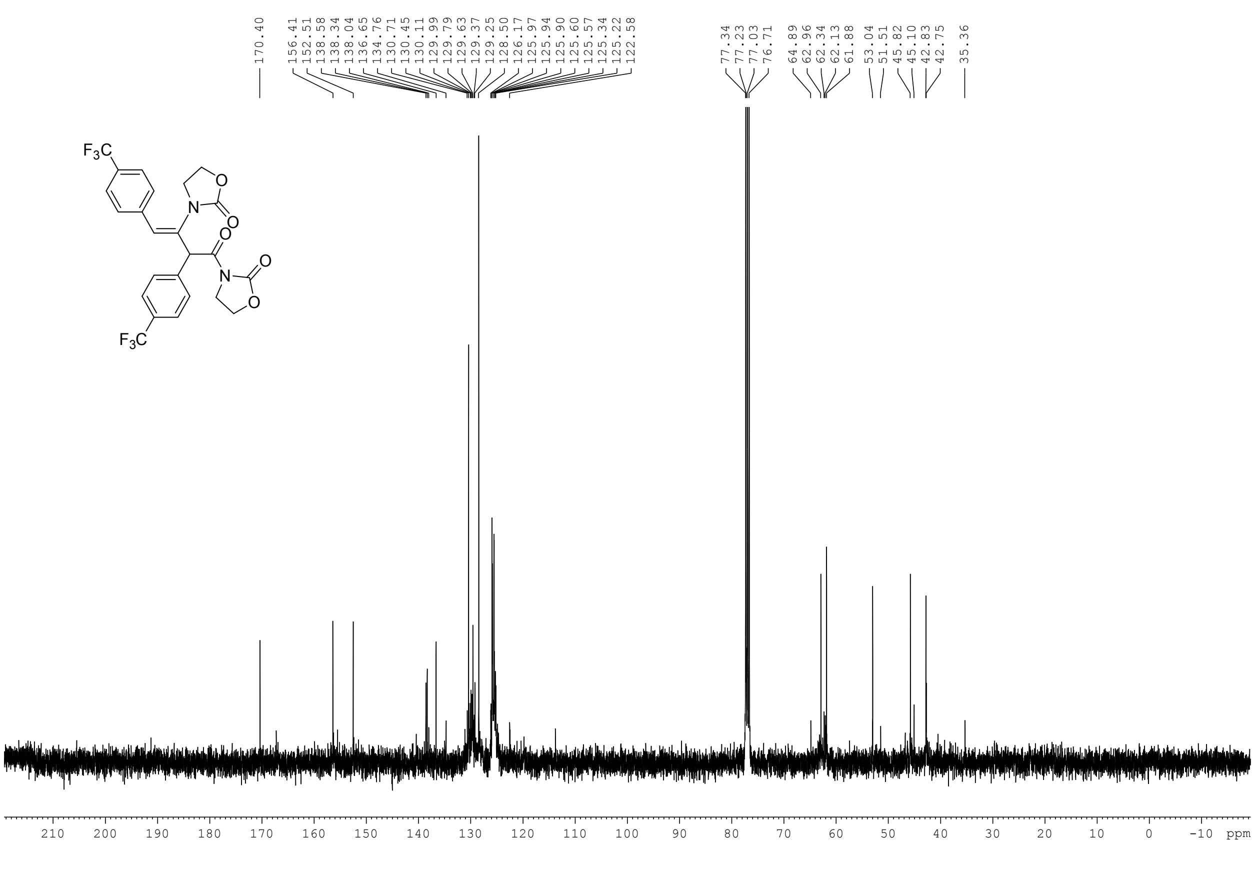
170.40
156.41
152.51
138.58
138.34
138.04
136.65
134.76
130.71
130.45
130.11
129.99
129.79
129.63
129.37
129.25
128.50
126.17
125.97
125.94
125.90
125.60
125.57
125.34
125.22
122.58
77.34
77.23
77.03
76.71
64.89
62.96
62.34
62.13
61.88
53.04
51.51
45.82
45.10
42.83
42.75
35.36
F3C
F3C
ppm

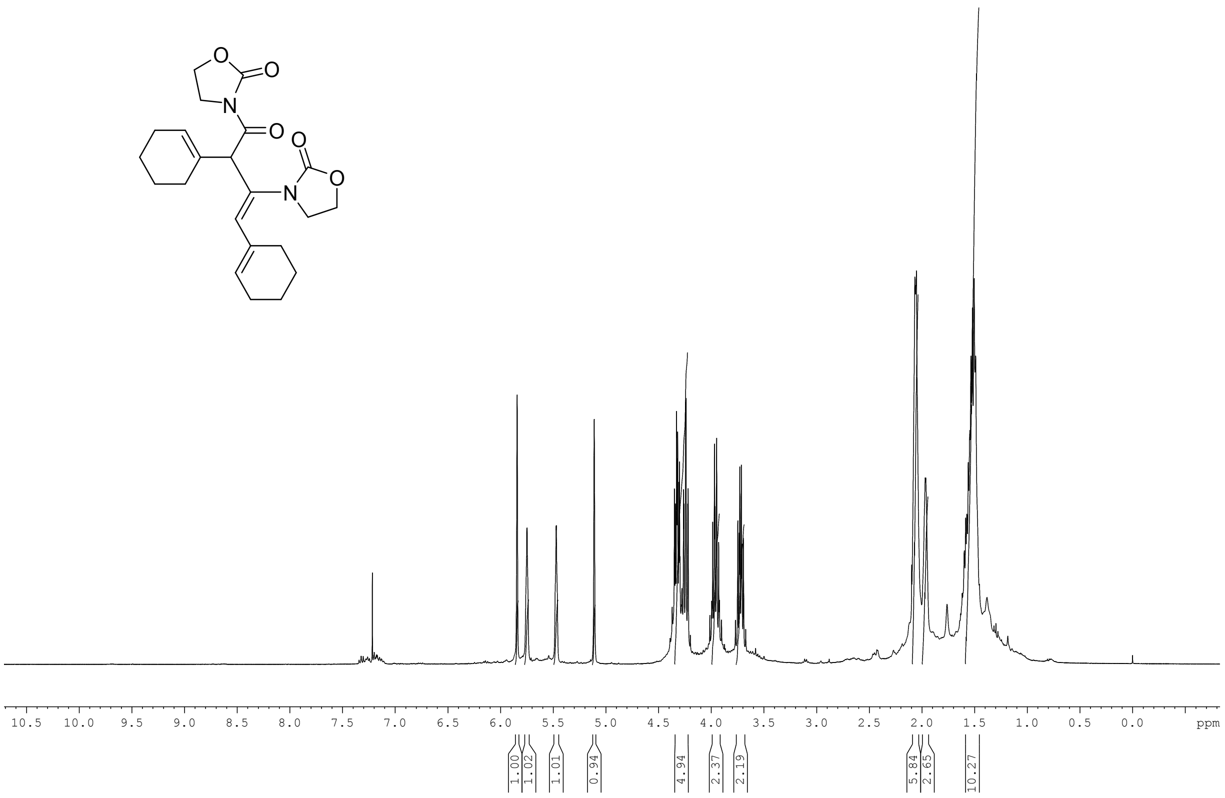
O
O
N
O
O
N
O
1.00
1.02
1.01
0.94
4.94
2.37
2.19
5.84
2.65
10.27
10.5 10.0 9.5 9.0 8.5 8.0 7.5 7.0 6.5 6.0 5.5 5.0 4.5 4.0 3.5 3.0 2.5 2.0 1.5 1.0 0.5 0.0 ppm

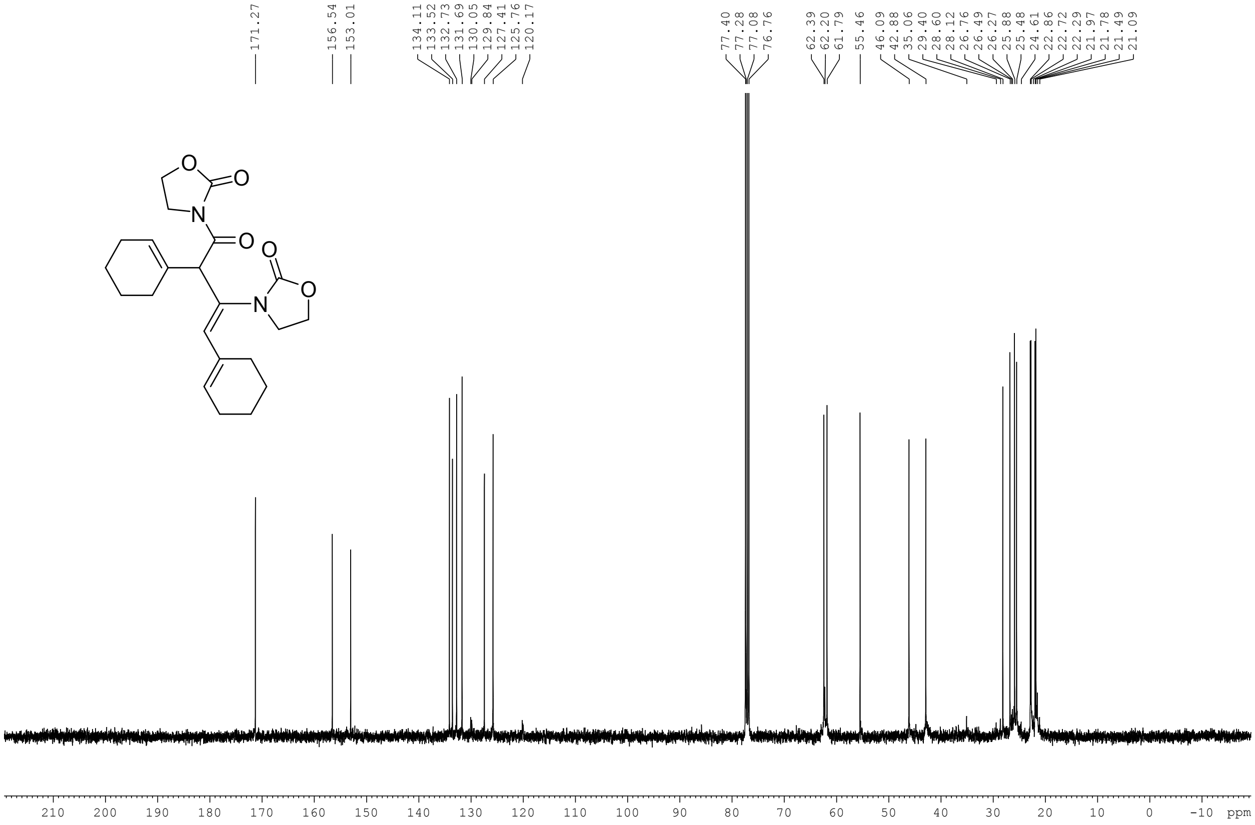
171.27
156.54
153.01
134.11
133.52
132.73
131.69
130.05
129.84
127.41
125.76
120.17
77.40
77.28
77.08
76.76
62.39
62.20
61.79
55.46
46.09
42.88
35.06
29.40
28.60
28.12
26.76
26.49
26.27
25.88
25.48
24.61
22.86
22.72
22.29
21.97
21.78
21.49
21.09
O
O
N
O
O
N
O
210 200 190 180 170 160 150 140 130 120 110 100 90 80 70 60 50 40 30 20 10 0 -10 ppm

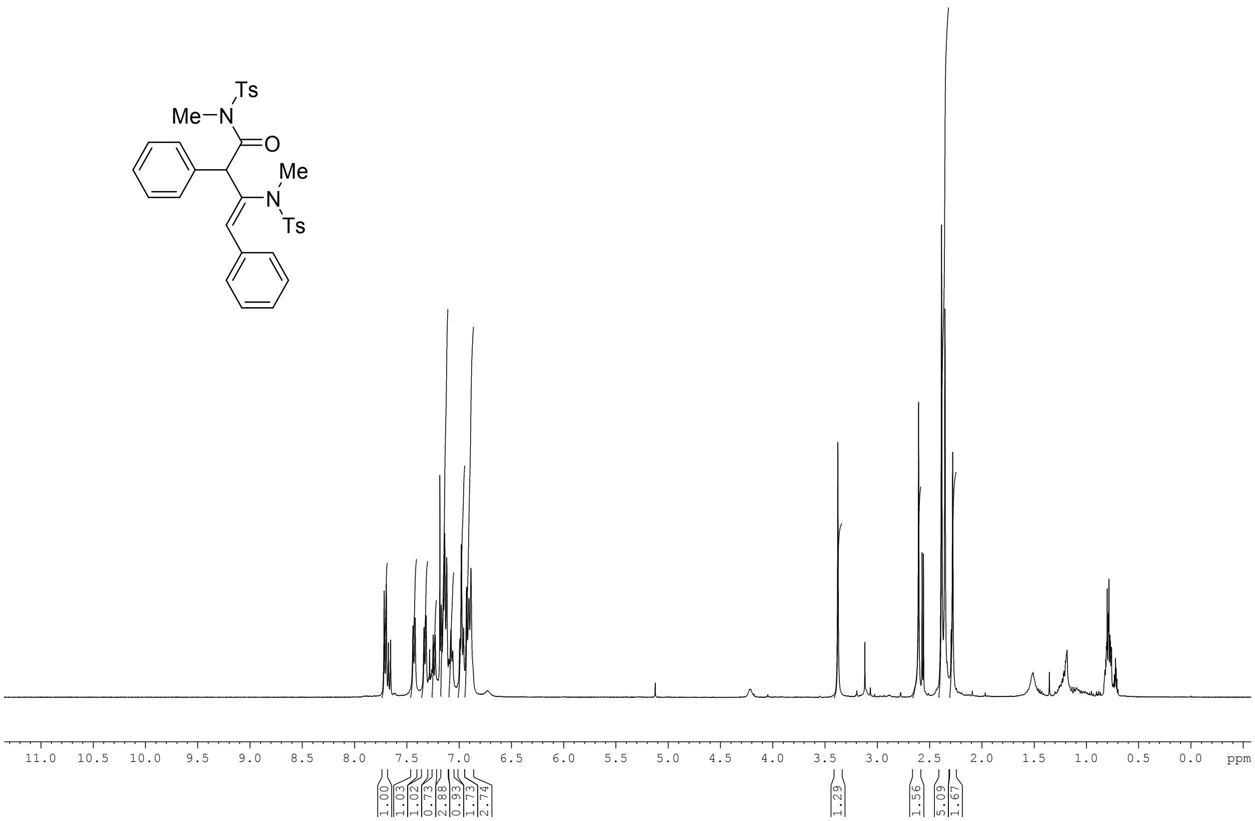
Ts
Me−N
O
Me
N
Ts
11.0 10.5 10.0 9.5 9.0 8.5 8.0 7.5 7.0 6.5 6.0 5.5 5.0 4.5 4.0 3.5 3.0 2.5 2.0 1.5 1.0 0.5 0.0 ppm
1.00
1.03
1.02
0.73
2.88
0.93
1.73
2.74
1.29
1.56
5.09
1.67

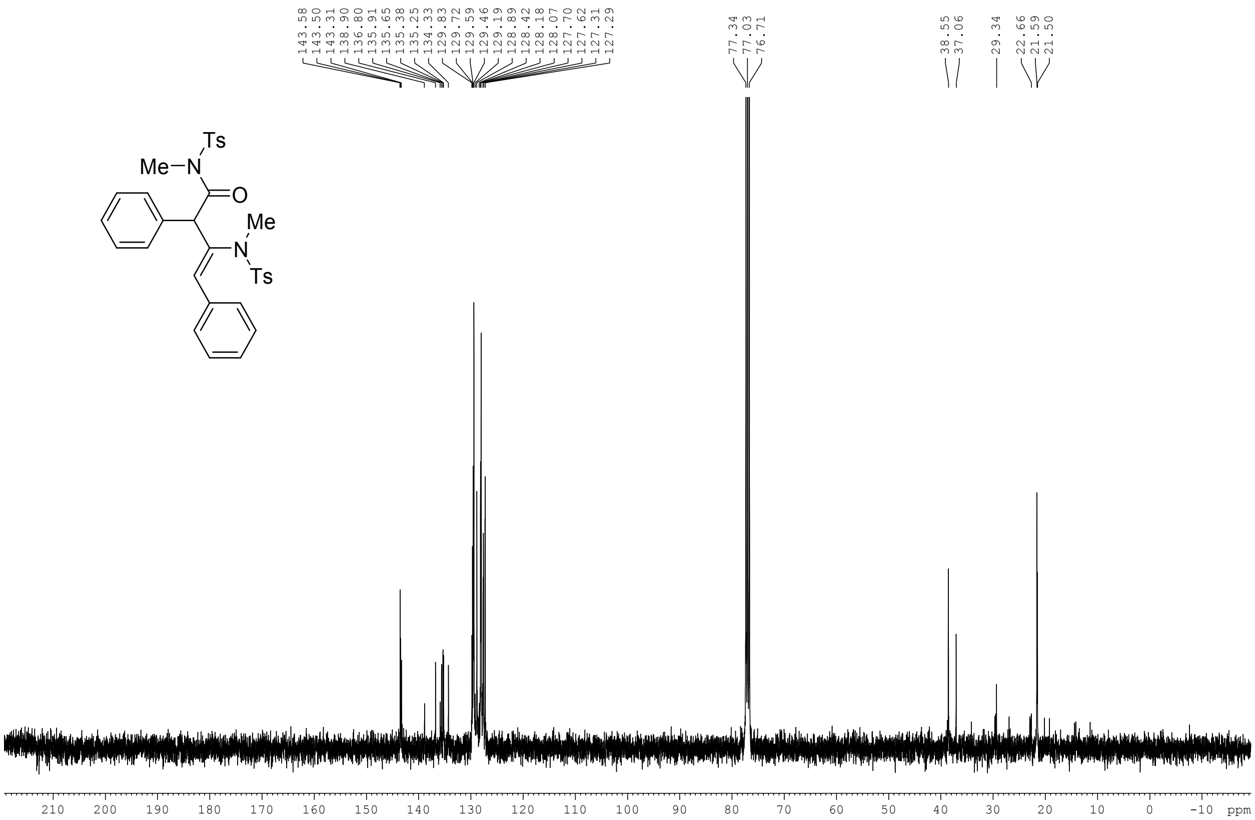
143.58
143.50
143.31
138.90
136.80
135.91
135.65
135.38
135.25
134.33
129.83
129.72
129.59
129.46
129.19
128.89
128.42
128.18
128.07
127.70
127.62
127.31
127.29
77.34
77.03
76.71
38.55
37.06
29.34
22.66
21.59
21.50
Ts
Me−N
O
Me
N
Ts
210 200 190 180 170 160 150 140 130 120 110 100 90 80 70 60 50 40 30 20 10 0 -10 ppm

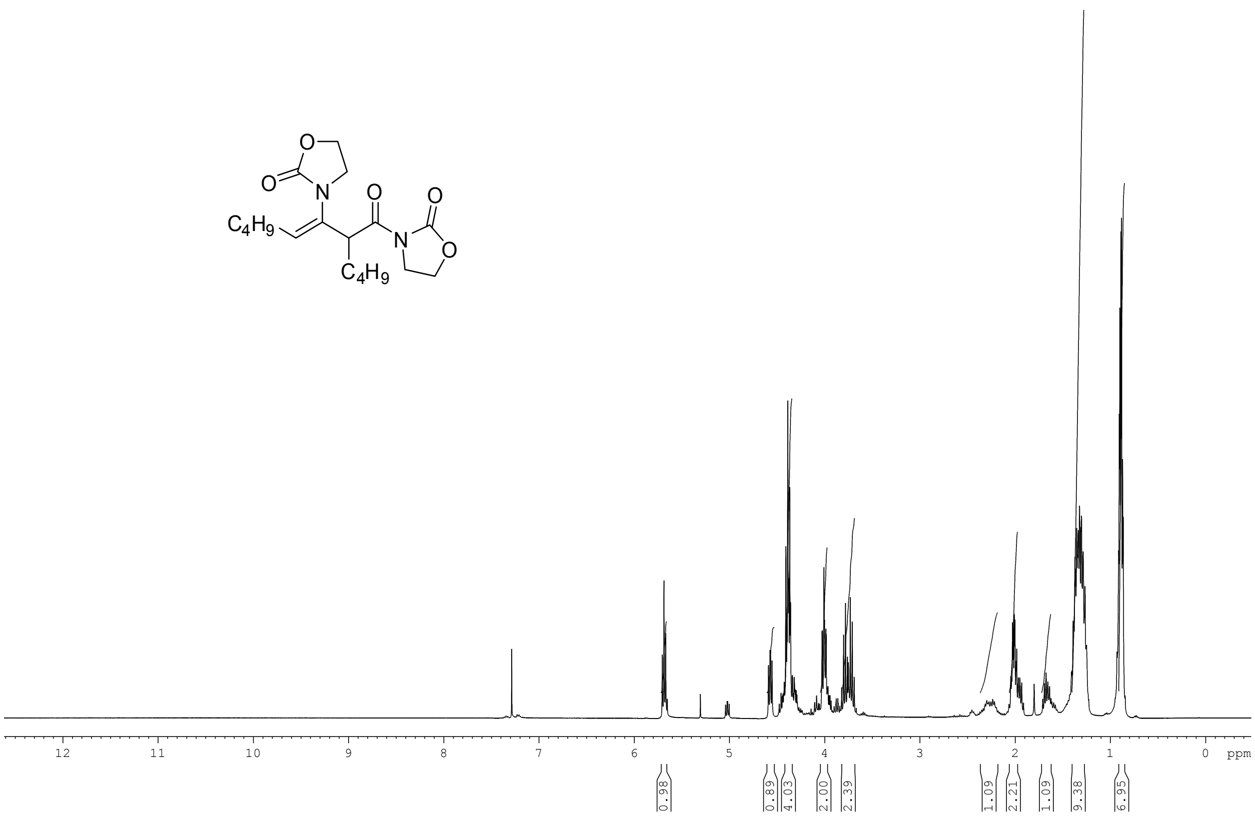
O
N
O
O
N
O
O
C_4H_9
C_4H_9
ppm
0.98
0.89
4.03
2.00
2.39
1.09
2.21
1.09
9.38
6.95

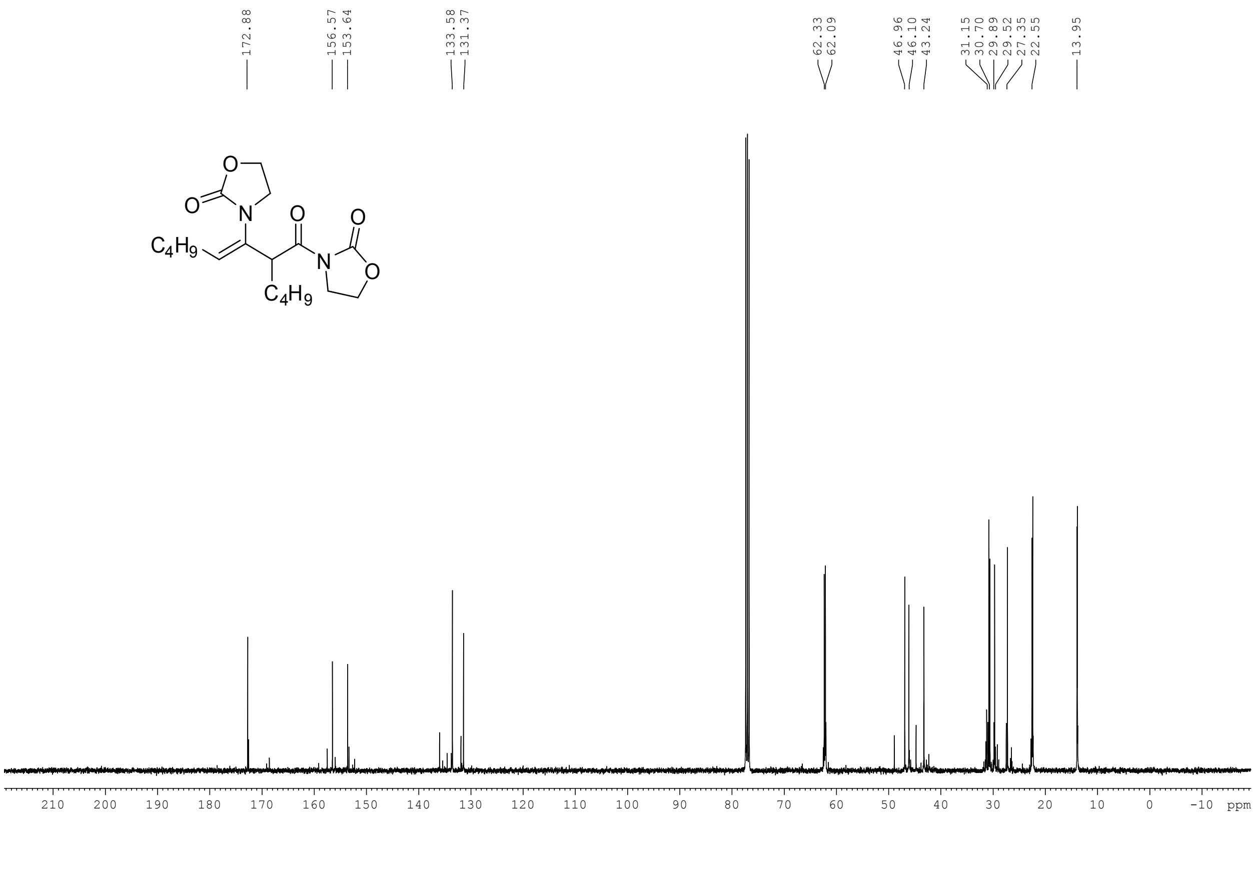
172.88
156.57
153.64
133.58
131.37
62.33
62.09
46.96
46.10
43.24
31.15
30.70
29.89
29.52
27.35
22.55
13.95
O
N
O
O
N
O
O
C_4H_9
C_4H_9
ppm

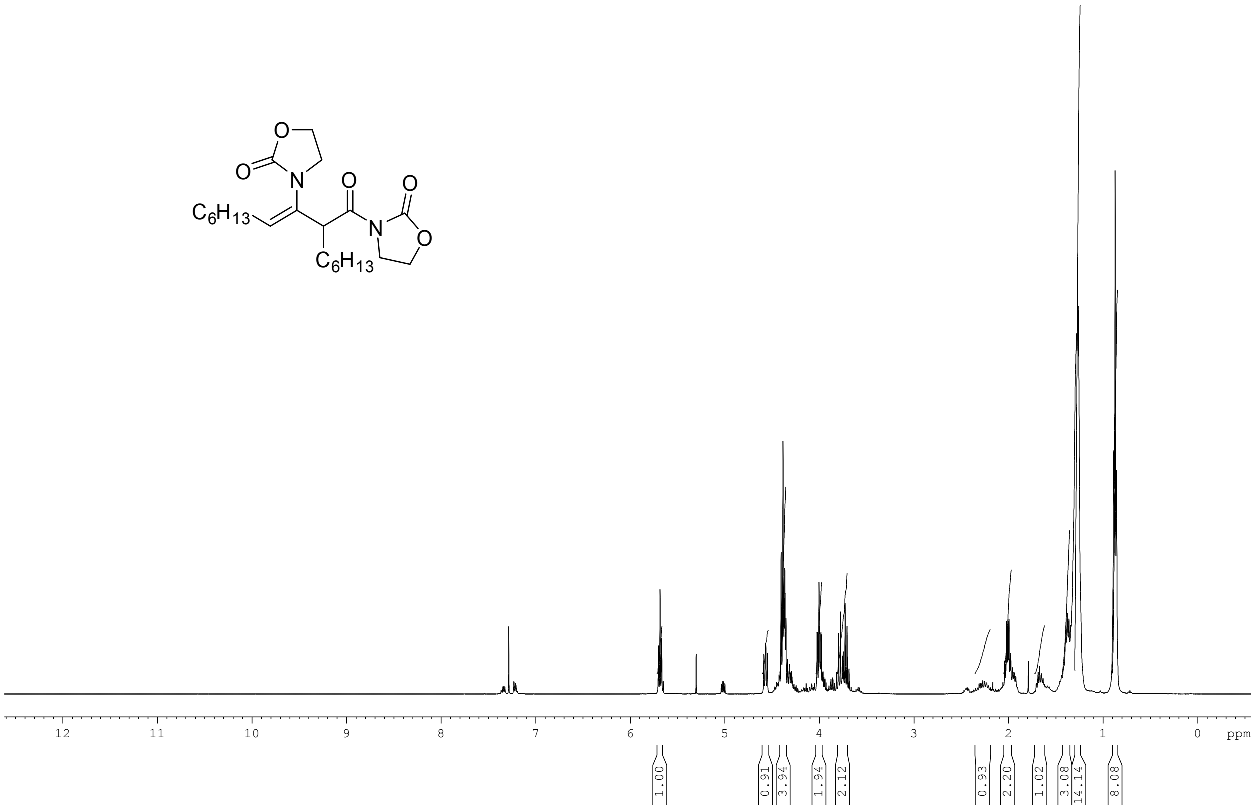

O
N
O
C6H13
O
N
O
O
C6H13
12 11 10 9 8 7 6 5 4 3 2 1 0 ppm
1.00
0.91
3.94
1.94
2.12
0.93
2.20
1.02
3.08
14.14
8.08

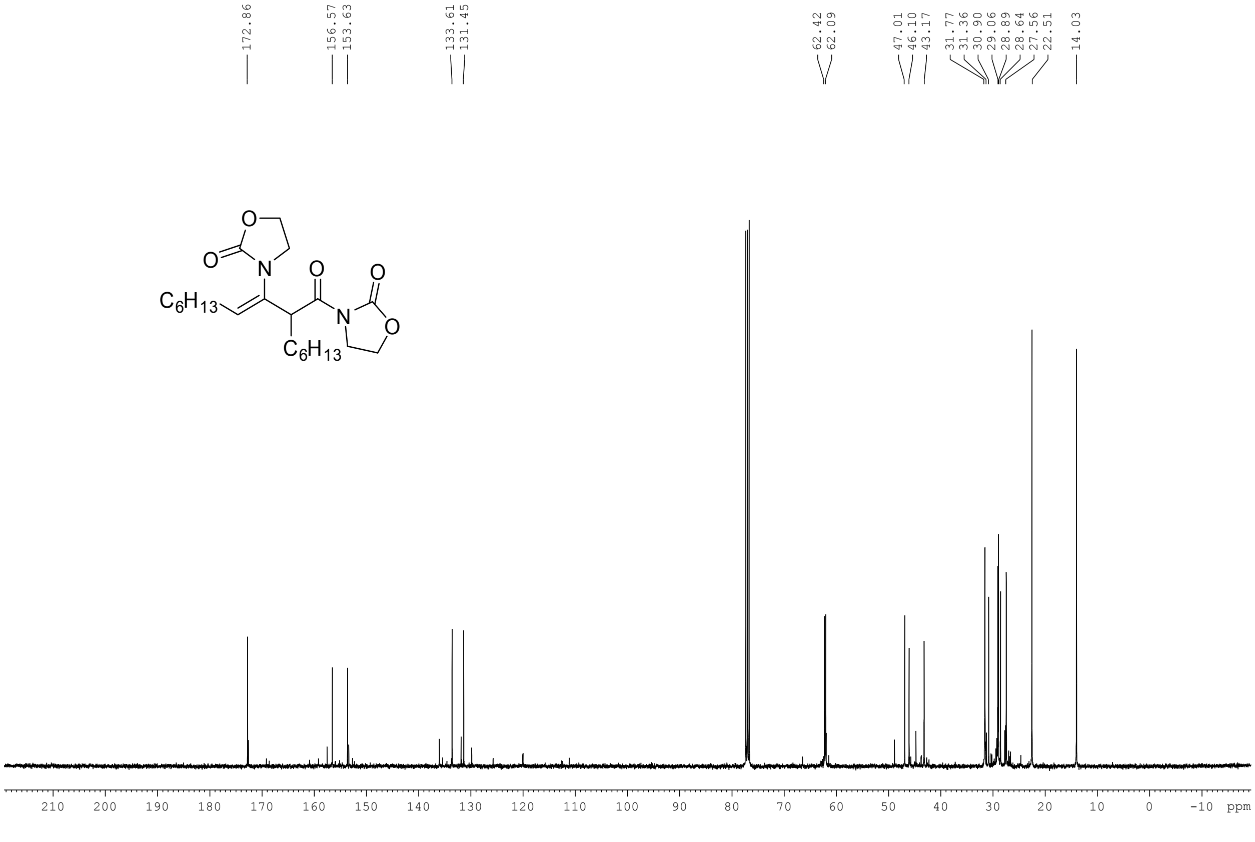

172.86
156.57
153.63
133.61
131.45
62.42
62.09
47.01
46.10
43.17
31.77
31.36
30.90
29.06
28.89
28.64
27.56
22.51
14.03
O
N
O
C6H13
O
N
O
O
C6H13
210 200 190 180 170 160 150 140 130 120 110 100 90 80 70 60 50 40 30 20 10 0 -10 ppm

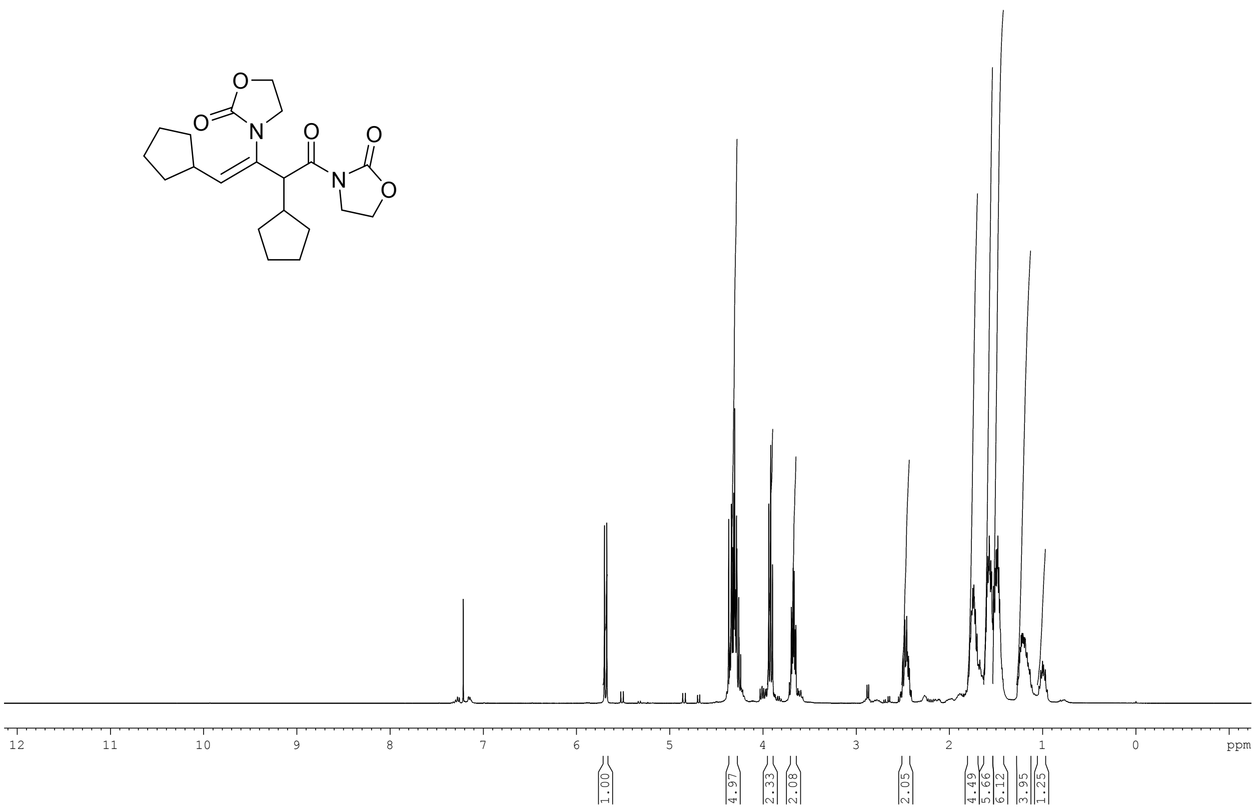

O
N
O
N
O
O
O
12 11 10 9 8 7 6 5 4 3 2 1 0 ppm
1.00
4.97
2.33
2.08
2.05
4.49
5.66
6.12
3.95
1.25

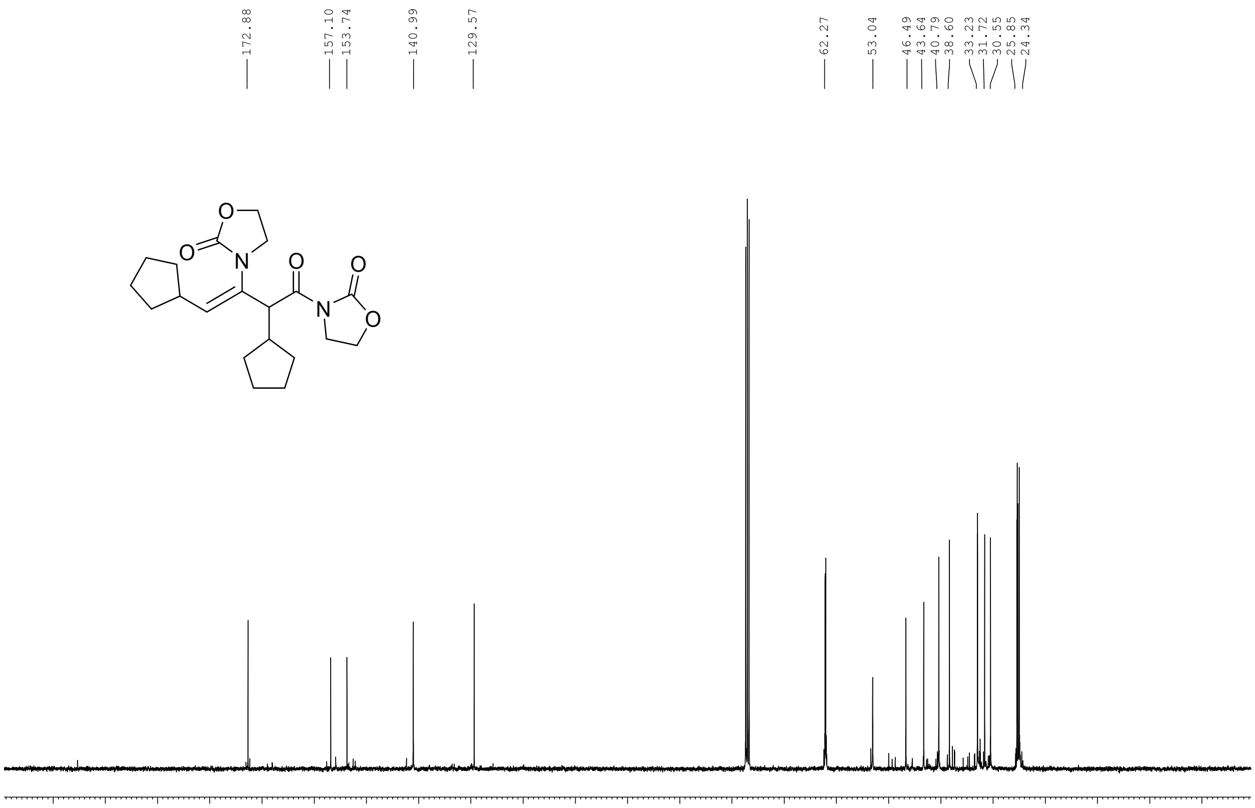

172.88
157.10
153.74
140.99
129.57
62.27
53.04
46.49
43.64
40.79
38.60
33.23
31.72
30.55
25.85
24.34
O
N
O
N
O
O
O
210 200 190 180 170 160 150 140 130 120 110 100 90 80 70 60 50 40 30 20 10 0 -10 ppm

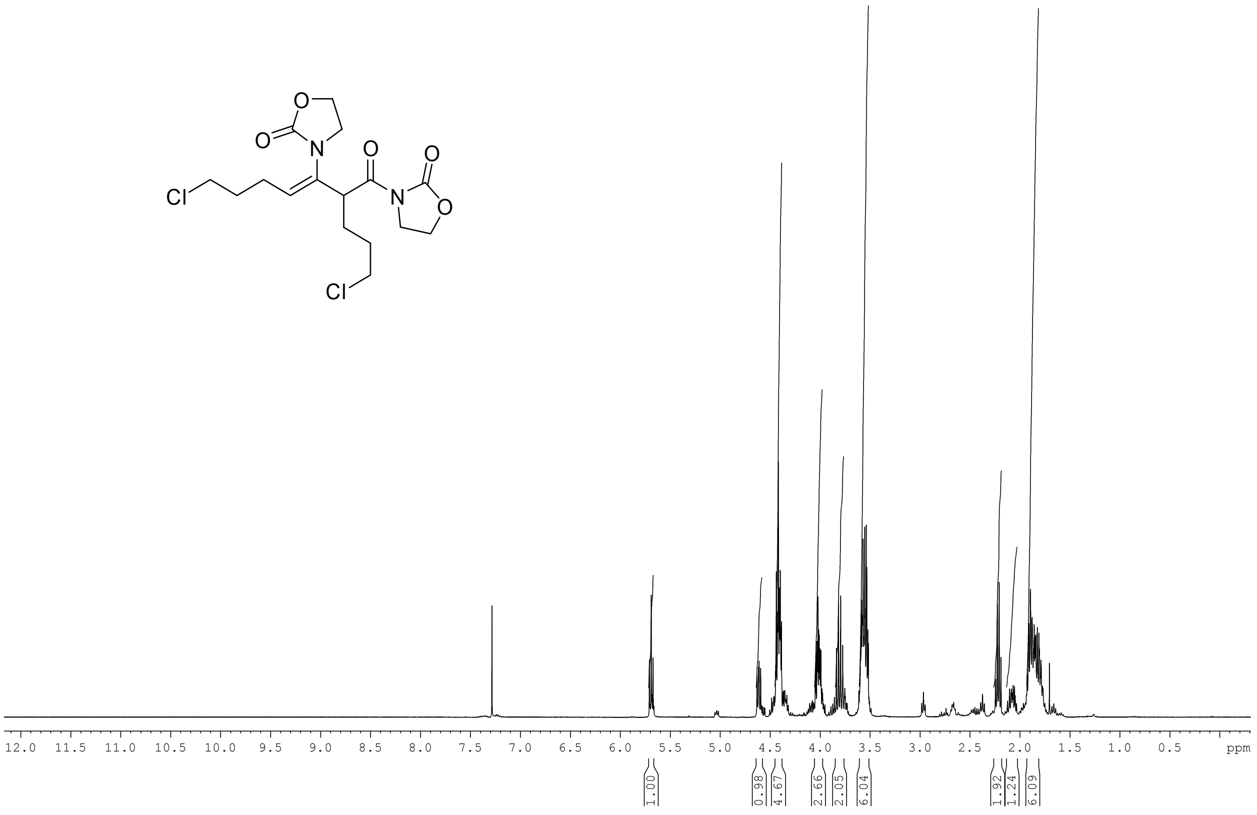
Cl
Cl
O
N
O
O
N
O
O
ppm
1.00
0.98
4.67
2.66
2.05
6.04
1.92
1.24
6.09

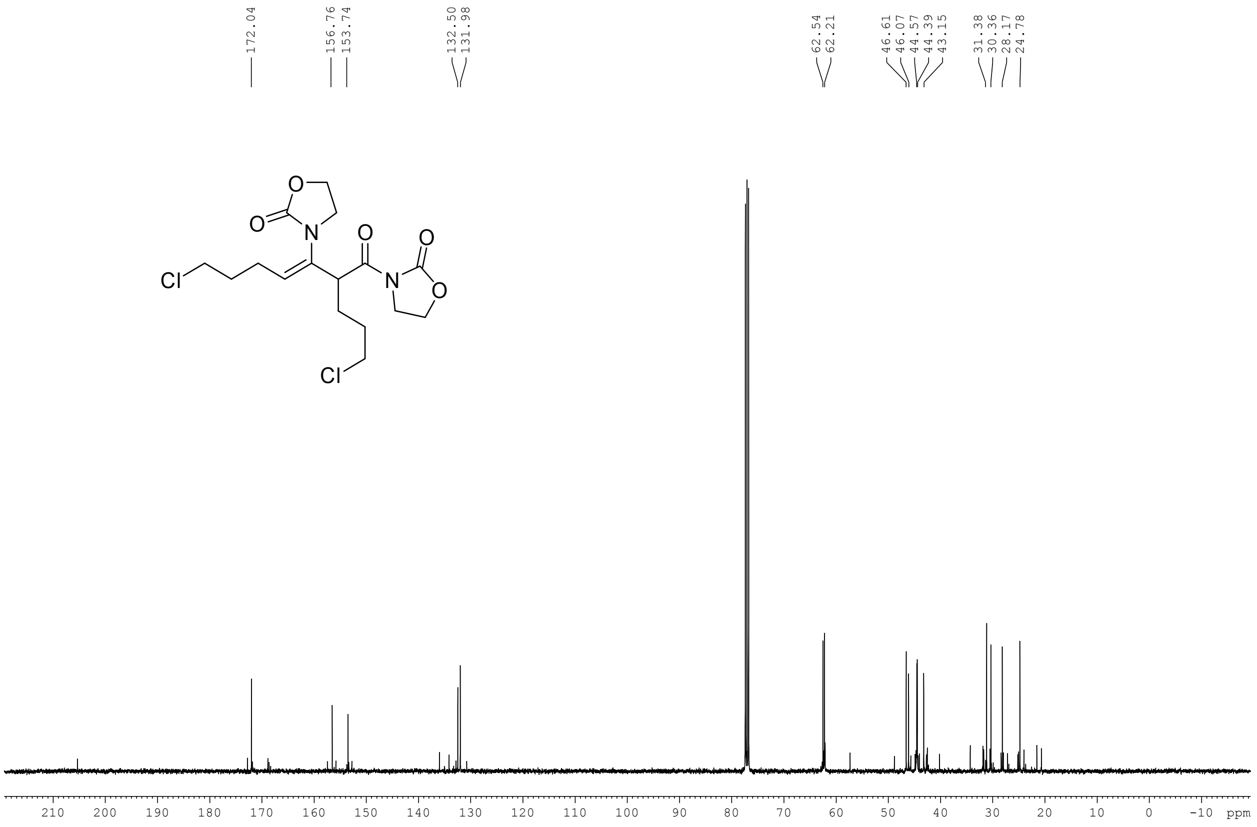
172.04
156.76
153.74
132.50
131.98
62.54
62.21
46.61
46.07
44.57
44.39
43.15
31.38
30.36
28.17
24.78
Cl
Cl
O
N
O
O
N
O
O
ppm

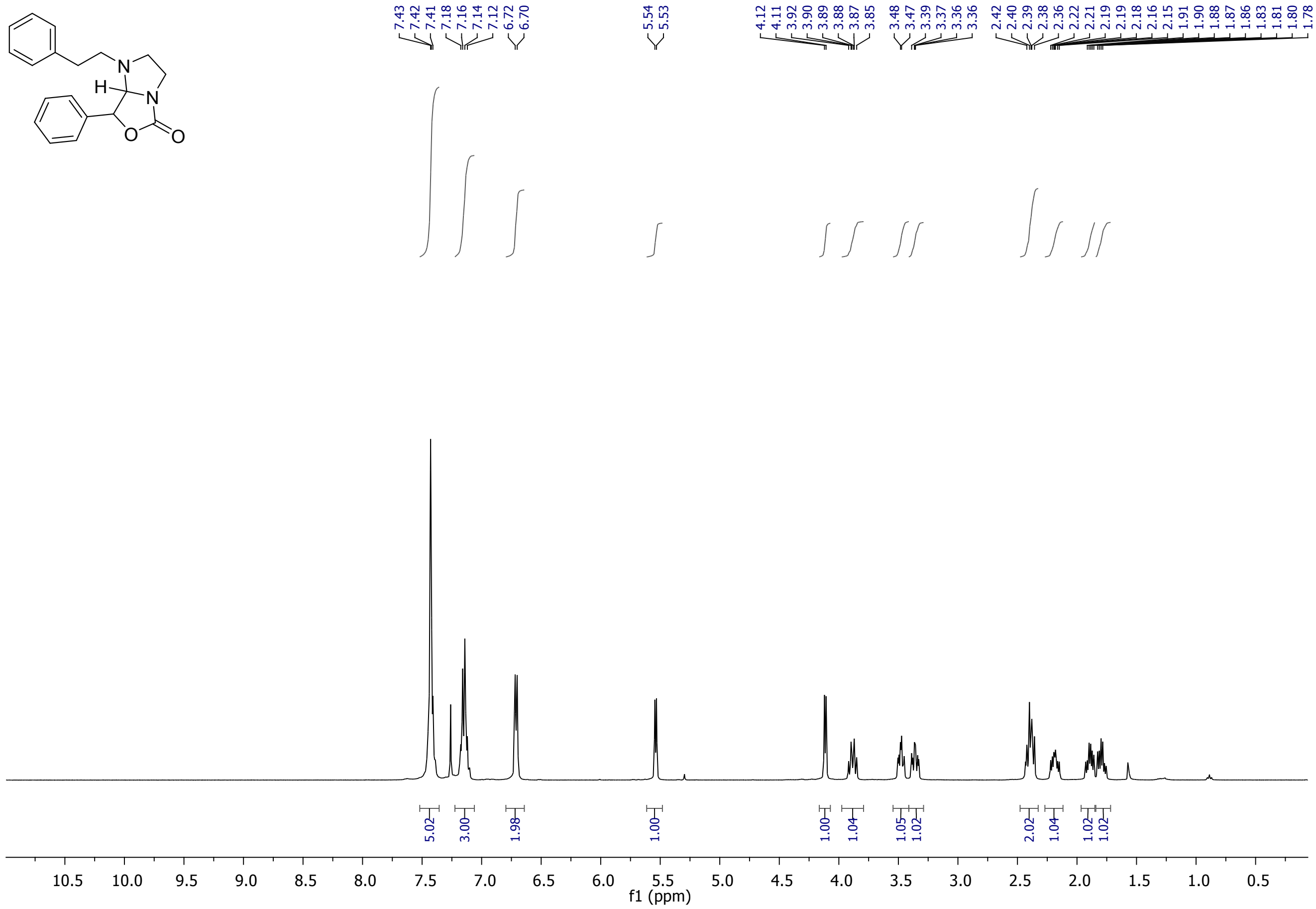
7.43 7.42 7.41 7.18 7.16 7.14 7.12 6.72 6.70
5.54 5.53
4.12 4.11 3.92 3.90 3.89 3.88 3.87 3.85
3.48 3.47 3.39 3.37 3.36 3.36
2.42 2.40 2.39 2.38 2.36 2.22 2.21 2.19 2.19 2.18 2.16 2.15 1.91 1.90 1.88 1.87 1.86 1.83 1.81 1.80 1.78
5.02 3.00 1.98 1.00 1.00 1.04 1.05 1.02 2.02 1.04 1.02 1.02
10.5 10.0 9.5 9.0 8.5 8.0 7.5 7.0 6.5 6.0 5.5 5.0 4.5 4.0 3.5 3.0 2.5 2.0 1.5 1.0 0.5
f1 (ppm)

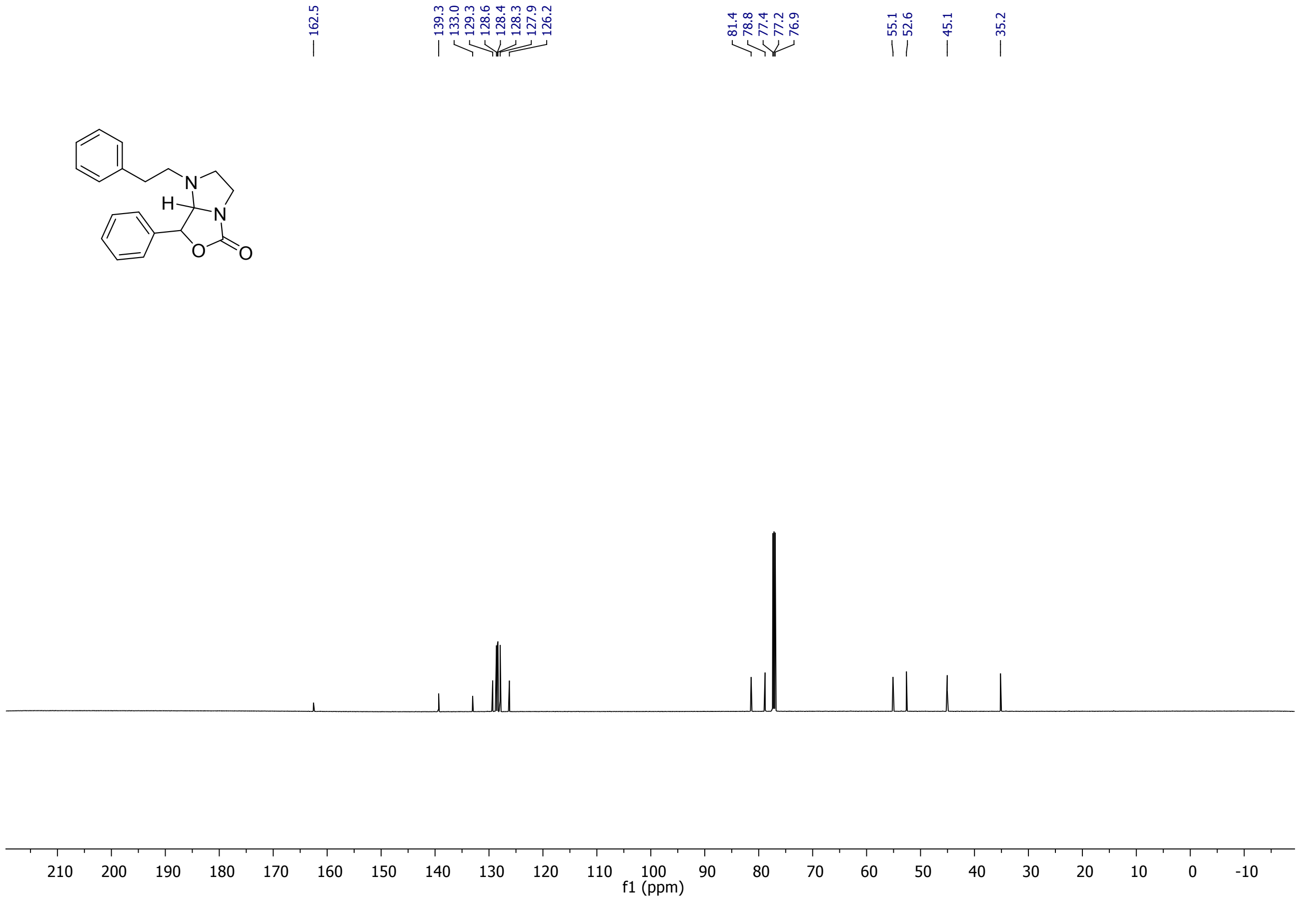
162.5
139.3 133.0 129.3 128.6 128.4 128.3 127.9 126.2
81.4 78.8 77.4 77.2 76.9
55.1 52.6 45.1 35.2
210 200 190 180 170 160 150 140 130 120 110 100 90 80 70 60 50 40 30 20 10 0 -10
f1 (ppm)

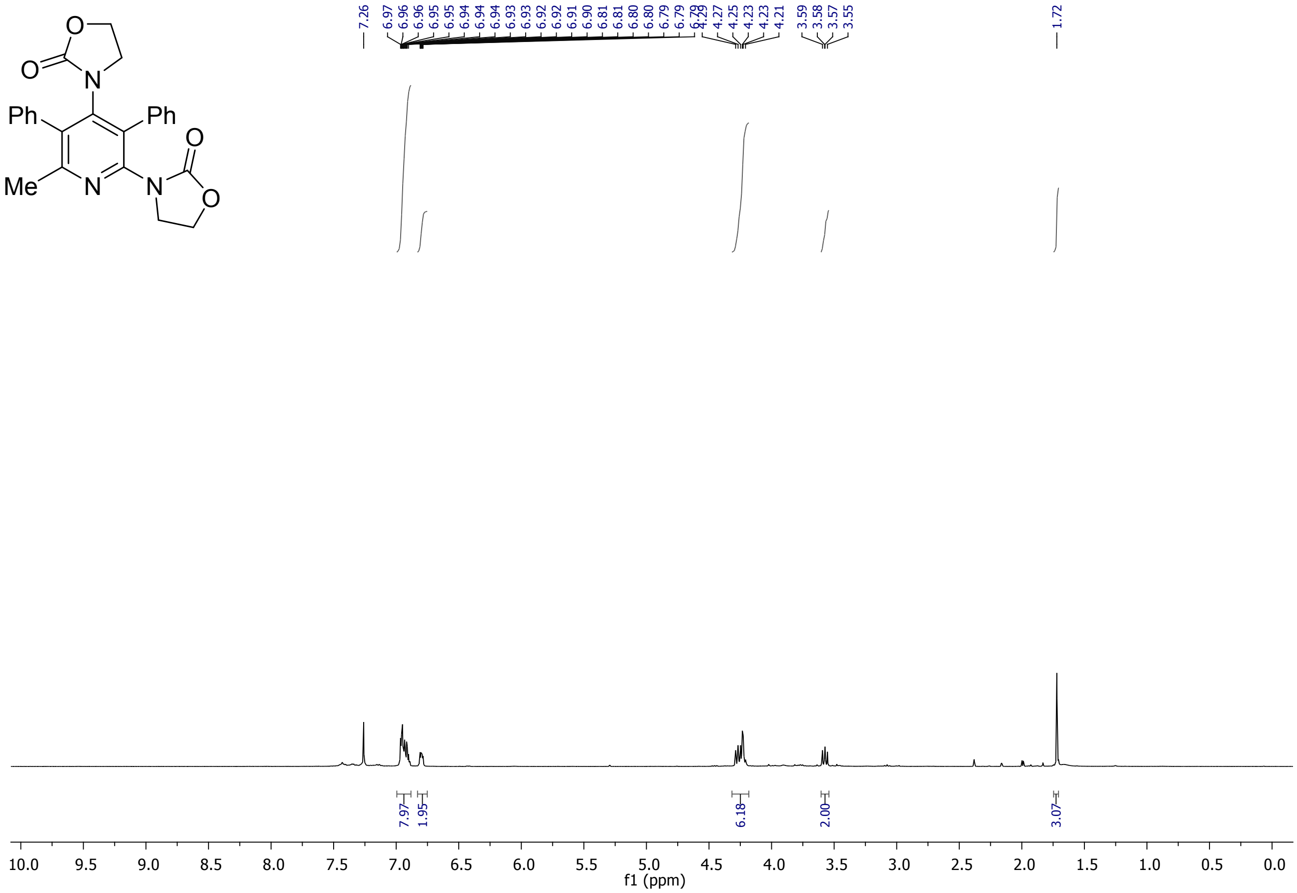
Ph
Ph
Me
N
O
O
N
O
N
O
7.26
6.97 6.96 6.96 6.95 6.94 6.94 6.94 6.93 6.93 6.92 6.92 6.91 6.90 6.81 6.81 6.80 6.80 6.79 6.79 6.79 4.29 4.27 4.25 4.23 4.21
3.59 3.58 3.57 3.55
1.72
7.97
1.95
6.18
2.00
3.07
f1 (ppm)

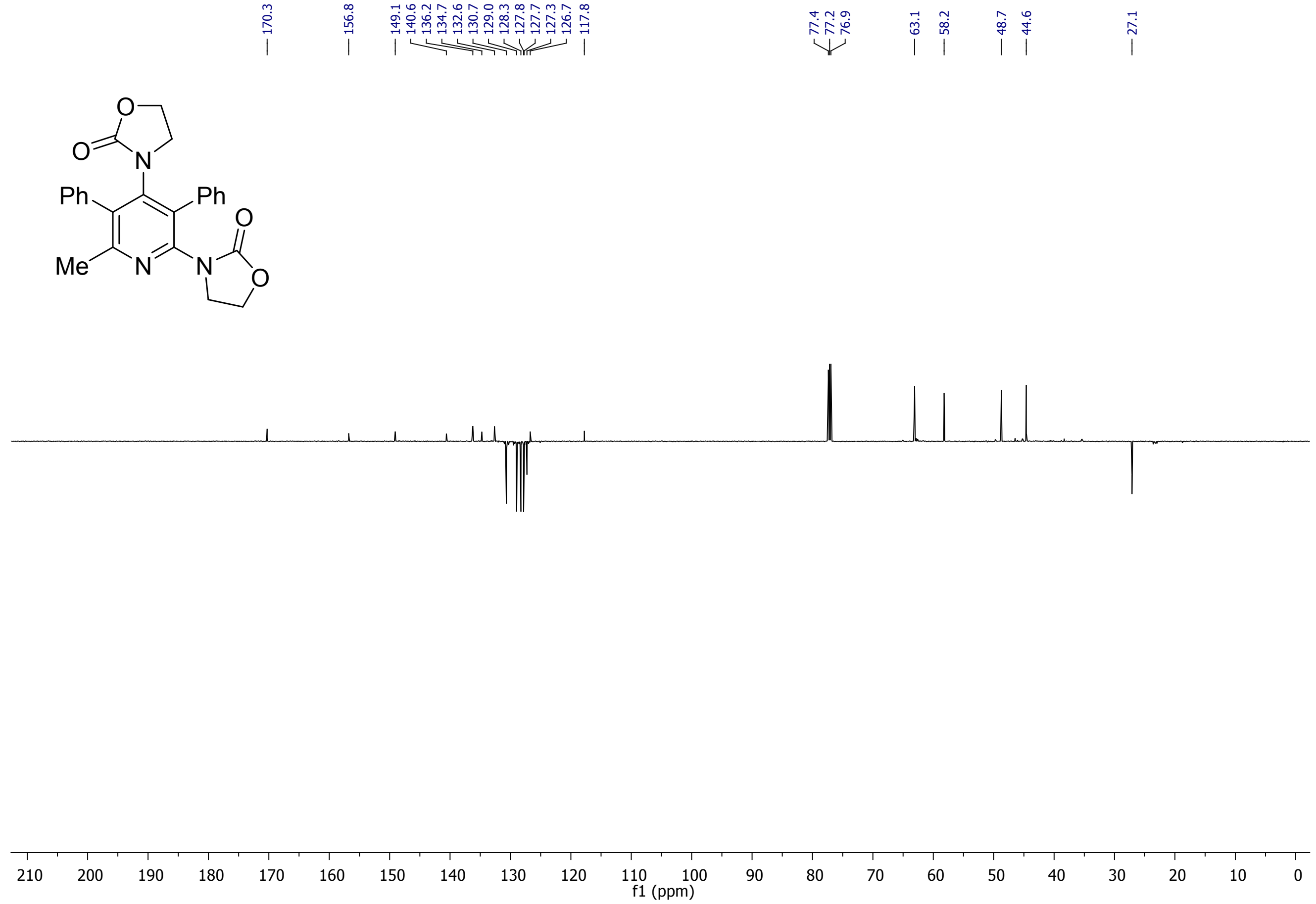
170.3
156.8
149.1 140.6 136.2 134.7 132.6 130.7 129.0 128.3 127.8 127.7 127.3 126.7 117.8
77.4 77.2 76.9
63.1
58.2
48.7
44.6
27.1
Ph
Ph
Me
N
O
O
N
O
N
O
f1 (ppm)

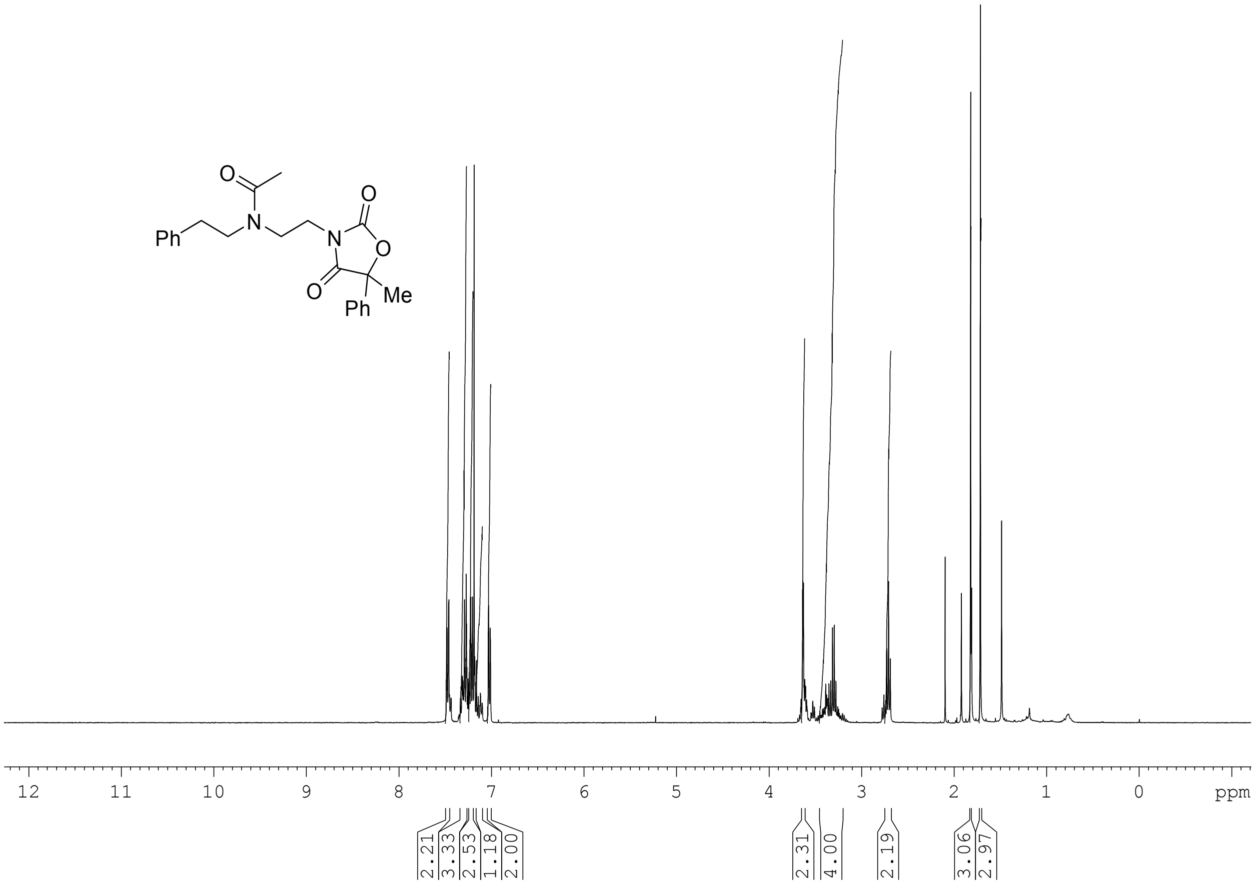

Ph
N
O
N
O
O
O
Ph
Me
12
11
10
9
8
7
6
5
4
3
2
1
0
ppm
2.21
3.33
2.53
1.18
2.00
2.31
4.00
2.19
3.06
2.97

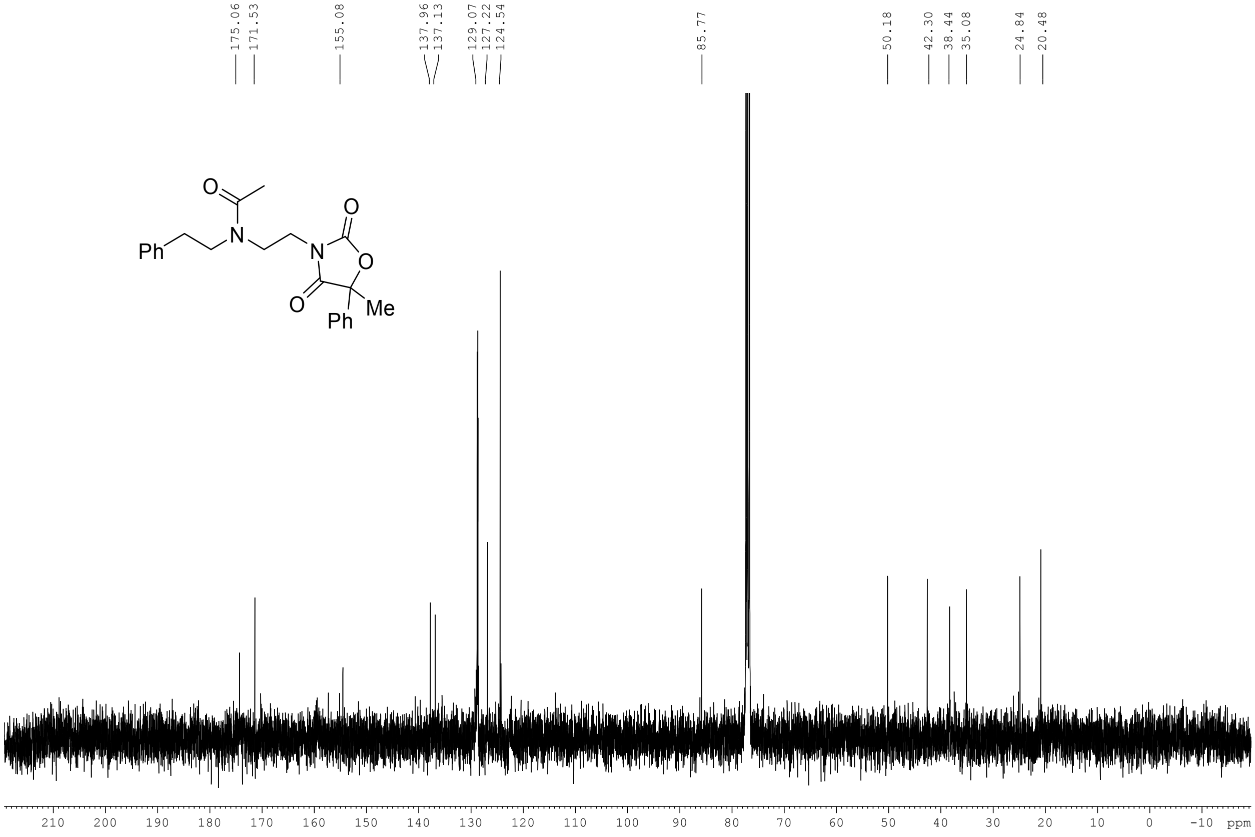

175.06
171.53
155.08
137.96
137.13
129.07
127.22
124.54
85.77
50.18
42.30
38.44
35.08
24.84
20.48
Ph
N
O
N
O
O
O
Ph
Me
210
200
190
180
170
160
150
140
130
120
110
100
90
80
70
60
50
40
30
20
10
0
-10
ppm

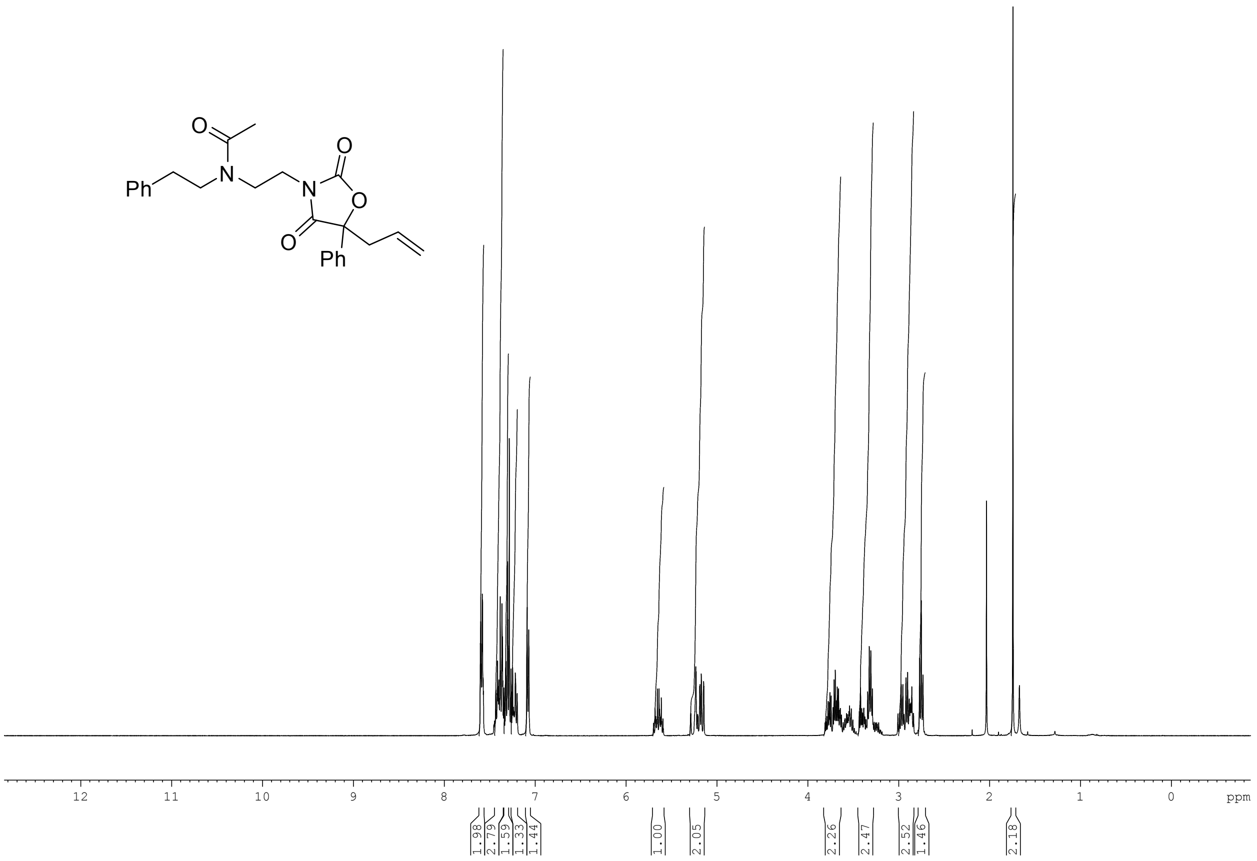
O
N
Ph
N
O
O
O
Ph
12 11 10 9 8 7 6 5 4 3 2 1 0 ppm
1.98
2.79
1.59
1.33
1.44
1.00
2.05
2.26
2.47
2.52
1.46
2.18

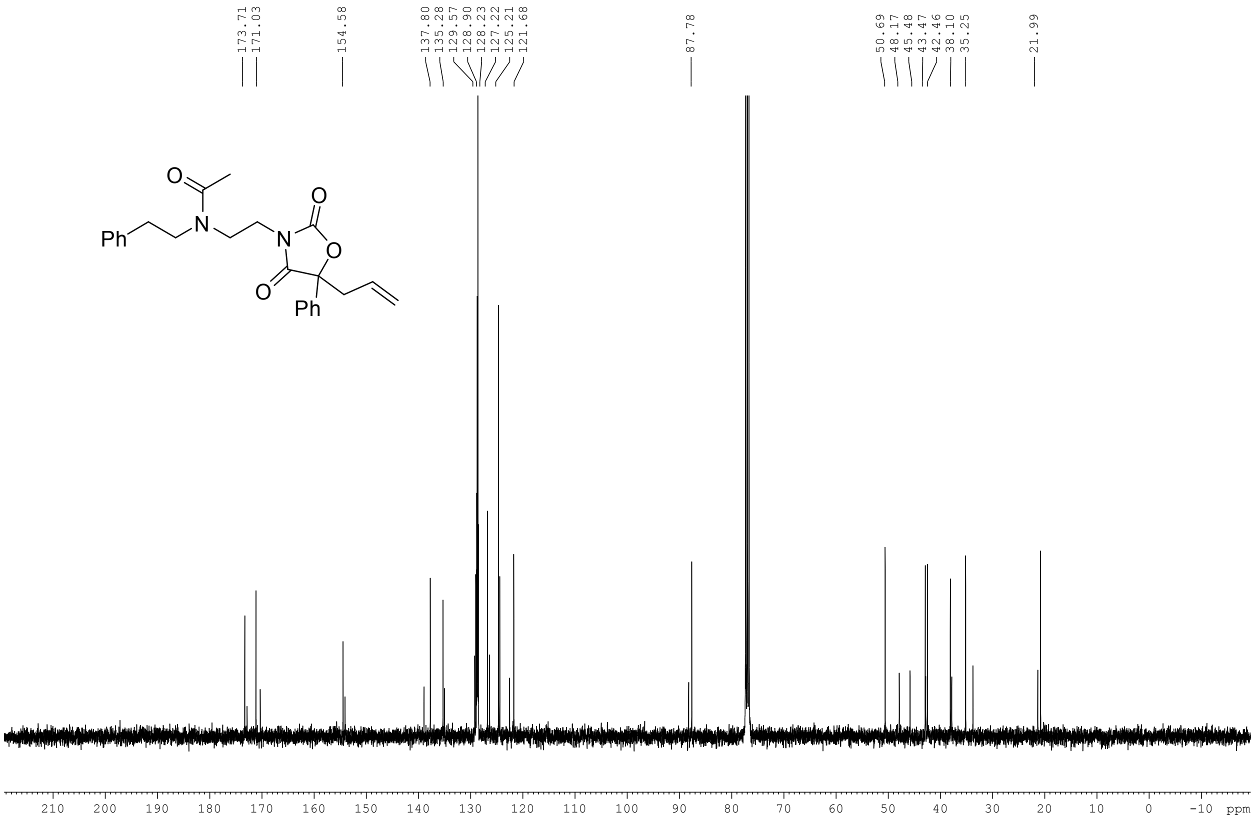
173.71
171.03
154.58
137.80
135.28
129.57
128.90
128.23
127.22
125.21
121.68
87.78
50.69
48.17
45.48
43.47
42.46
38.10
35.25
21.99
O
N
Ph
N
O
O
O
Ph
210 200 190 180 170 160 150 140 130 120 110 100 90 80 70 60 50 40 30 20 10 0 -10 ppm

10- X-rax analysis

The X-ray intensity data were measured on a Bruker X8-APEXII equipped with multilayer monochromators, Mo K/a INCOATEC micro focus sealed tube and Kryoflex II cooling device. The structures were solved by charge flipping and refined by full-matrix least-squares techniques. Non-hydrogen atoms were refined with anisotropic displacement parameters. Hydrogen atoms were inserted at calculated positions and refined with a riding model. The following software was used: Frame integration, *Bruker SAINT software package*[2] using a narrow-frame algorithm, Absorption correction, *SADABS*[3], structure solution, *SHELXL-2013*[4], refinement, *SHELXL-2013*[4], *OLEX2*[5], *SHELXLE*[6], molecular diagrams, *OLEX2*[5]. Experimental data and CCDC-Code can be found in Table 1. Crystal data, data collection parameters, and structure refinement details are given in Tables 2 to 5. Molecular Structures are displayed in Figure 1, 2 and 3.

Table 1. Experimental parameter and CCDC-Code.

Sample	Machine	Source	Temp.	Detector Distance	Time/ Frame	#Frames	Frame width	CCDC
	Bruker		[K]	[mm]	[s]		[°]	
3a.HBF4	X8	Mo	100	40	80	1007	0.4	1439068
7a	X8	Mo	100	35.1	30	1942	0.5	1439069

2-(2,4-dioxo-5-phenyloxazolidin-3-yl)-N-phenethylethan-1-aminiumtetrafluoroborate [3a.HBF4].

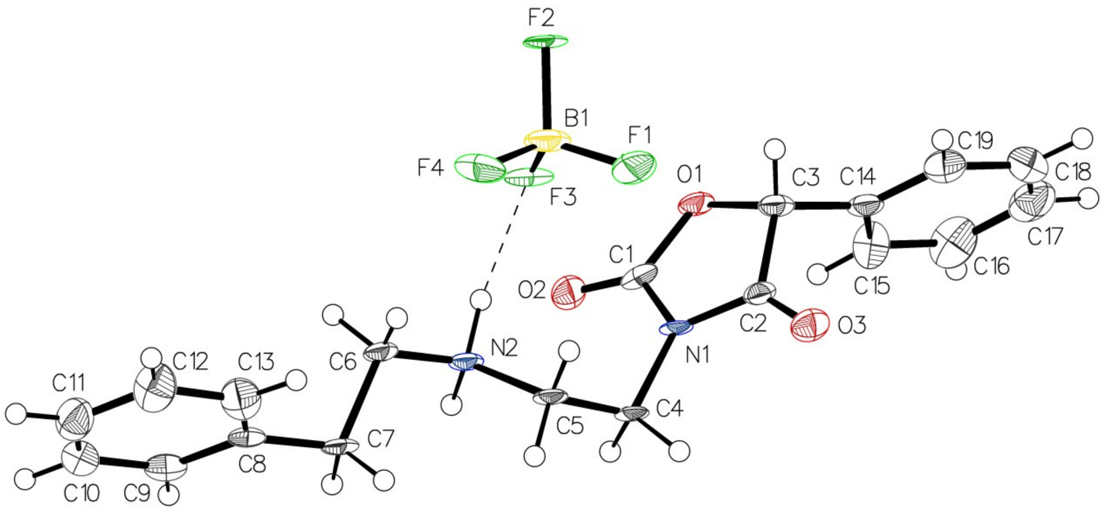

Figure 1. Asymmetric Unit of [**3a.HBF4**], drawn with 50% displacement ellipsoids. Co-crystalized solvent omitted for clarity.

Table 2. Sample and crystal data of [**3a.HBF4**].

**Chemical formula**	C20H23BCl2F4N2O3	**Crystal system**	monoclinic	
**Formula weight [g/mol]**	497.11	**Space group**	*P2₁/c*	
**Temperature [K]**	100	**Z**	4	
**Measurement method**	\Φ and \ω scans	**Volume [Å^3]**	2242.3(8)	
**Radiation (Wavelength [Å])**	MoKα (λ = 0.71073)	**Unit cell dimensions [Å] and [°]**	16.897(4)	90
**Crystal size / [mm^3]**	0.2 × 0.05 × 0.05		12.002(3)	101.315(7)
**Crystal habit**	clear colourless block		11.276(2)	90
**Density (calculated) / [g/cm^3]**	1.473	**Absorption coefficient / [mm^{-1}]**	0.348	
**Abs. correction Tmin**	0.5729	**Abs. correction Tmax**	0.7452	
**Abs. correction type**	multi-scan	**F(000) [e$^-$]**	1024	

Table 3. Data collection and structure refinement of [**3a.HBF4**].

**Index ranges**	-20 ≤ h ≤ 19, -14 ≤ k ≤ 14, -13 ≤ l ≤ 13	**Theta range for data collection [°]**	4.918 to 51.25	
**Reflections number**	14498	**Data / restraints / parameters**	4112/42/289	
**Refinement method**	Least squares	**Final R indices**	all data	R1 = 0.1367, wR2 = 0.1757
**Function minimized**	Σ w(F_o^2 - F_c^2)^2		I>2σ(I)	R1 = 0.0655, wR2 = 0.1455
**Goodness-of-fit on F^2**	0.95	**Weighting scheme**	w=1/[$\sigma^2(F_o^2)+(0.0889P)^2$]	
**Largest diff. peak and hole [e Å^{-3}]**	0.56/-0.49		where P=(F_o^2+2F_c^2)/3	

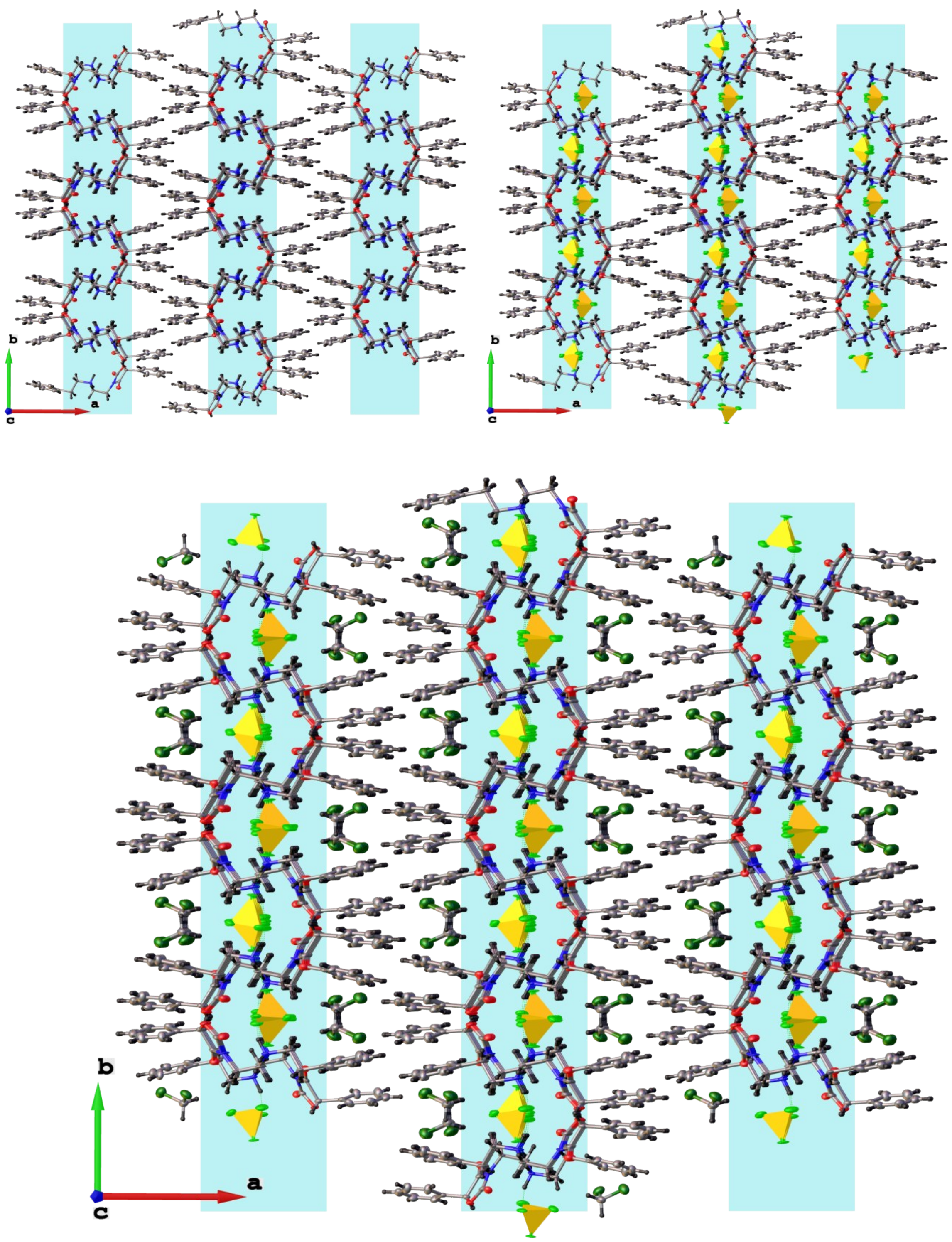

Figure 2. Packing diagram of [**3a.HBF4**]. Characterized by two layers, one polar (blue background) and one non polar. The scheme follows ABAB. Enveloped by the polar parts of [**3a.HBF4**] the BF_4^- counter ion is mainly responsible for the defined packing. The plane bc is arranged parallel to the layers. It is not surprisingly that axis a is longer than b and c and the main growth directions of the crystals should be along b and c. Non polar voids are matching with the size of the available dichloromethane.

1-Phenethyl-7-phenyltetrahydro-1H,5H-imidazo[1,2-c]oxazol-5-one [7a].

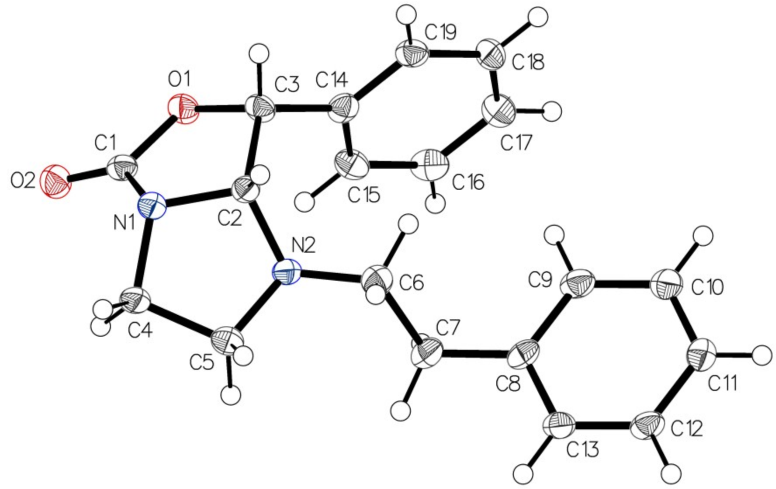

Figure 3. Asymmetric Unit of [**7a**], drawn with 50% displacement ellipsoids. Although the space group is *P2₁*, no interpretation of the two chiral centers is possible.

Table 4. Sample and crystal data of [**7a**].

**Chemical formula**	C19H20N2O2	**Crystal system**	monoclinic	
**Formula weight [g/mol]**	308.37	**Space group**	*$P2_1$*	
**Temperature [K]**	100	**Z**	2	
**Measurement method**	\Φ and \ω scans	**Volume [Å^3]**	796.21(11)	
**Radiation (Wavelength [Å])**	MoKα (λ = 0.71073)	**Unit cell dimensions [Å] and [°]**	6.0369(5)	90
**Crystal size / [mm^3]**	0.1 × 0.07 × 0.03		15.2528(11)	107.568(4)
**Crystal habit**	clear colourless block		9.0700(8)	90
**Density (calculated) / [g/cm^3]**	1.286	**Absorption coefficient / [mm^{-1}]**	0.084	
**Abs. correction Tmin**	0.6615	**Abs. correction Tmax**	0.7456	
**Abs. correction type**	multi-scan	**F(000) [e$^-$]**	328	

Table 5. Data collection and structure refinement of [**7a**].

**Index ranges**	$-7 \leq h \leq 7$, $-18 \leq k \leq 18$, $-10 \leq l \leq 10$	**Theta range for data collection [°]**	5.342 to 50.696

**Reflections number**	16944	**Data / restraints / parameters**	2911/1/208	
**Refinement method**	Least squares	**Final R indices**	all data	R1 = 0.0667, wR2 = 0.1640
**Function minimized**	Σ w(F_o^2 - F_c^2)^2		I>2σ(I)	R1 = 0.0625, wR2 = 0.1590
**Goodness-of-fit on F^2**	1.119	**Weighting scheme**	w=1/[$\sigma^2(F_o^2)+(0.1140P)^2+0.1501P$]	
**Largest diff. peak and hole [e Å^{-3}]**	0.47/-0.29		where $P=(F_o^2+2F_c^2)/3$	

11- References:

[1] a) Hamada, T.; Ye, X.; Stahl, S. *J. Am. Chem. Soc*. **2008**, *130*, 833; b) Jin, X.; Yamaguchi, K.; Mizuno, N. *Chem. Commun*., **2012**, *48*, 4974; c) Alvarez, S. G.; Alvarez, M. T. *Synthesis*, **1997**, *4*, 413; d) Peng, B.; Xueliang, H.; Xie, L.; Maulide, N.; Angew. Chem. Int. Ed. **2014**, *53*, 8718.

[2] Bruker SAINT V7.68A Copyright © 2005-2015 Bruker AXS.

[3] Sheldrick, G. M. (1996). *SHELXS.* University of Göttingen, Germany.

[4] Sheldrick, G.M. (2008). Acta Cryst. A64, 112-122

[5] Dolomanov, O.V., Bourhis, L.J., Gildea, R.J, Howard, J.A.K. & Puschmann, H. , OLEX2, (2009), J. Appl. Cryst. 42, 339-341.

[6] C. B. Huebschle, G. M. Sheldrick and B. Dittrich, ShelXle: a Qt graphical user interface for SHELXL, J. Appl. Cryst., 44, (2011) 1281-1284
